# TBP regulates transposable element expression in early mouse embryos

**DOI:** 10.1038/s44318-026-00736-w

**Published:** 2026-03-20

**Authors:** Clara Hermant, Carlos Michel Mourra-Díaz, Marlies E Oomen, Natasha Jansz, Camille Noll, Antoine Canat, Mrinmoy Pal, Tsunetoshi Nakatani, Tamas Schauer, Maria-Elena Torres-Padilla

**Affiliations:** 1 Institute of Epigenetics and Stem Cells (IES), Helmholtz Zentrum München, D-81377 München, Germany; 2 Faculty of Biology, Ludwig-Maximilians Universität, München, Germany

**Keywords:** Mouse Embryos, Retrotransposons, MaLR, TBP, ERVL, Chromatin, Transcription & Genomics, Development

## Abstract

The activation of the embryonic genome is a crucial step in development. In addition to thousands of genes, many transposable elements (TEs) are robustly transcribed during early mammalian development. However, their transcriptional regulators remain largely unexplored. Here, we set out to identify transcription factors regulating the expression of TEs from the LINE, SINE and ERVL families during mouse preimplantation development. In particular, the MaLR family are the most abundant ERVL in the mouse genome and are also the most abundant constituent of the transcriptome in early mouse embryos. We find that the general transcription factor TBP binds and activates MaLRs in mouse embryos. Loss-of-function of TBP leads to downregulation of MaLRs, specifically the ORR1A family, which is the youngest ORR subclass and contributes a significant portion of major zygotic genome activation transcripts. Our work identifies regulators of TE expression in vivo and highlights a previously unrecognised role for the general transcription factor TBP in regulating a highly specific TE transcriptional programme.

## Synopsis

Transposable elements are robustly expressed during zygotic genome activation in early mouse embryos, yet the transcription factors regulating their activity remain incompletely understood. ATAC-seq footprinting and targeted functional screening identify the TATA-binding protein TBP as a regulator of MaLR transposable elements and associated zygotic genome activation genes at the 2-cell stage.

- Footprinting analysis using ATAC-seq reads from preimplantation mouse embryos identifies 54 potential candidate TFs involved in transposable element (TE) regulation.
- A targeted gain-of-function screen in mouse embryonic stem cells (ESCs) identifies transcription factors which are expressed between the zygote and the 8-cell stage and impact TE expression.
- Acute depletion of TBP in embryos leads to extensive changes in transcription including half of the ZGA genes, which are downregulated.
- Chromatin profiling by CUT&Tag indicates that downregulated genes are bound by TBP in control embryos.
- MaLR family of TEs are bound by TBP and become significantly downregulated upon TBP loss.

## Abstract (web summary)

A targeted gain-of-function screen identifies TBP as a direct regulator of MaLR transposable elements during early mouse embryogenesis.

## Graphical abstract

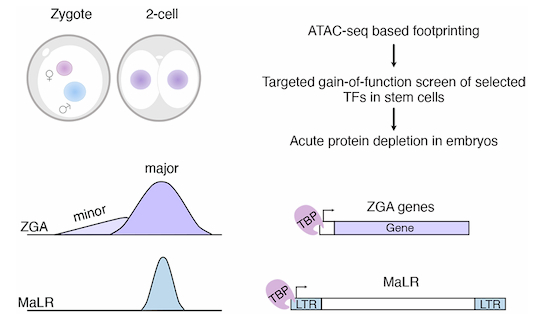

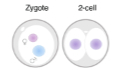

## Introduction

Transposable elements (TEs) and their remnants constitute an important and essential part of the transcriptional programme ensuing after fertilisation (Peaston et al, 2004; Evsikov et al, 2004; Fadloun et al, 2013; Franke et al, 2017; Jachowicz et al, 2017; Oomen et al, 2025). Research over the last years has shed light into the molecular mechanisms that maintain repression of TEs, but the proteins regulating their activation remain largely unknown, with only a few examples of transcription factors (TFs) characterised till now. In mouse embryos, a burst of transcriptional activation of TEs occurs after fertilisation in the zygote, peaking at the time of major zygotic genome activation (ZGA), which occurs at the late 2-cell stage (Peaston et al, 2004; Evsikov et al, 2004; Fadloun et al, 2013; Franke et al, 2017). A member of the LTR family, MERVL is highly expressed during this time and is considered both a marker of the 2-cell stage and as a proxy for successful ZGA (Svoboda et al, 2004; Macfarlan et al, 2012; Ishiuchi et al, 2015). Because of its potential role as regulatory element of ZGA genes, most work in recent years has focused on the identification of MERVL regulators, specifically on its LTR, MT2_Mm (Hendrickson et al, 2017; De Iaco et al, 2017; Whiddon et al, 2017; Choi et al, 2017; Zhang et al, 2019; Ji et al, 2023; Guo et al, 2024; Hermant et al, 2025). However, the degree of TE expression in early mouse development extends beyond MERVL and includes additional LTR families such as MaLR and also LINE and SINE elements (Peaston et al, 2004; Evsikov et al, 2004; Fadloun et al, 2013; Modzelewski et al, 2021; Oomen et al, 2025).

LINEs and SINEs are non-LTR-containing classes of retrotransposons, constituting 19.2% and 8.2% of the mouse genome, respectively (Waterston et al, 2002). Several LINE1 are expressed upon ZGA and their timely expression is important for full developmental competence (Jachowicz et al, 2017). A functional binding site for the transcription factor YY1 has been characterised in the murine LINE1 Tf monomers (DeBerardinis and Kazazian, 1999; Saha et al, 2024). YY1 binds to its LINE1 target sites in mouse embryonic stem cells (ESCs) that are devoid of DNA methylation (Cusack et al, 2020), and has been recently shown to contribute to LINE1 regulation in mouse embryos (Sakamoto and Ishiuchi, 2024). However, additional TFs involved remain to be discovered. SINE transcripts have been known to be present in mouse oocytes and early embryos for at least three decades (Taylor and Pikó, 1987), but how their transcriptional regulation is achieved is not fully understood. Overall, SINEs are transcribed from the 2-cell stage and their expression peaks later in development, around the morula stage (Bachvarova 1988; Fadloun et al, 2013; Oomen et al, 2025). The TF NR5A2 binds to SINE B1 elements in mouse pre-implantation embryos, but the extent of regulation of SINE B1 expression by NR5A2 is not fully clear (Gassler et al, 2022; Festuccia et al, 2024; Lai et al, 2023). SINE B2 elements contain the majority of the TE-derived loop anchors for 3D-genome organisation, presumably through the presence of CTCF binding sites (Schmidt et al, 2012), however, while CTCF has been shown to bind SINE B2 in early mouse embryos (Wang et al, 2024), no role in preimplantation development has been ascribed to these elements to date.

MaLR and MERVL share common ancestry (McCarthy and McDonald, 2004; Franke et al, 2017). MaLR is the major constituent of ERVL insertions in the mouse genome (85% of ERVL insertions) (Waterston et al, 2002). MaLR stands for “Mammalian apparent LTR Retrotransposon” as it contains a short non-coding internal sequence without any homology to retroviral proteins, and thus does not encode for any proteins (Smit, 1993). In rodents, MaLR are divided into two major families: ORR1 and MT. ORR1A is the youngest ORR1 representative, and together with ORR1B and ORR1C are found in both mouse and rat. These elements are evolutionary older than MT2_Mm (Franke et al, 2017). MaLRs from the MT family are highly expressed in mouse oocytes and their LTRs have been co-opted as oocyte-specific promoters (Peaston et al, 2004; Franke et al, 2017; Modzelewski et al, 2021). While co-option of ORR1s as alternative promoters in mammalian embryos has been documented (Peaston et al, 2004; Franke et al, 2017; Oomen et al, 2025), their regulation and potential benefits for the host are completely unexplored.

Here, we undertook a targeted, mid-scale gain-of-function screen informed by ATAC-seq footprinting analysis to identify potential transcriptional regulators of four major TEs highly expressed in mouse embryos: MT2_Mm, LINE L1 (Tf), SINE B2 and ERVL MaLRs. We identified 54 potential new candidate TFs involved in their transcriptional activation. We find that 10 of them induce transcription of one or several TEs in mouse ESCs. Based on their capacity to transactivate TEs when expressed in mouse ESCs, we further focus on two of them, TBP and FOXJ3. By performing loss-of-function and RNA-seq analyses, we show that TBP regulates MaLRs in mouse embryos at the time of ZGA as well as a significant portion of the ZGA transcriptome. Chromatin profiling in embryos demonstrates that TBP binds directly to its MaLR targets. Interestingly, TBP contributes to ZGA regulation independently of MT2_Mm, which we find is not regulated by TBP. Thus, our work identifies new TFs for TEs in vivo and highlights the role of a general basal transcription factor in regulating a specific part of the TE repertoire in the embryo.

## Results

### Identification of candidate TFs regulating TEs in early mouse embryos

**Figure 1:**
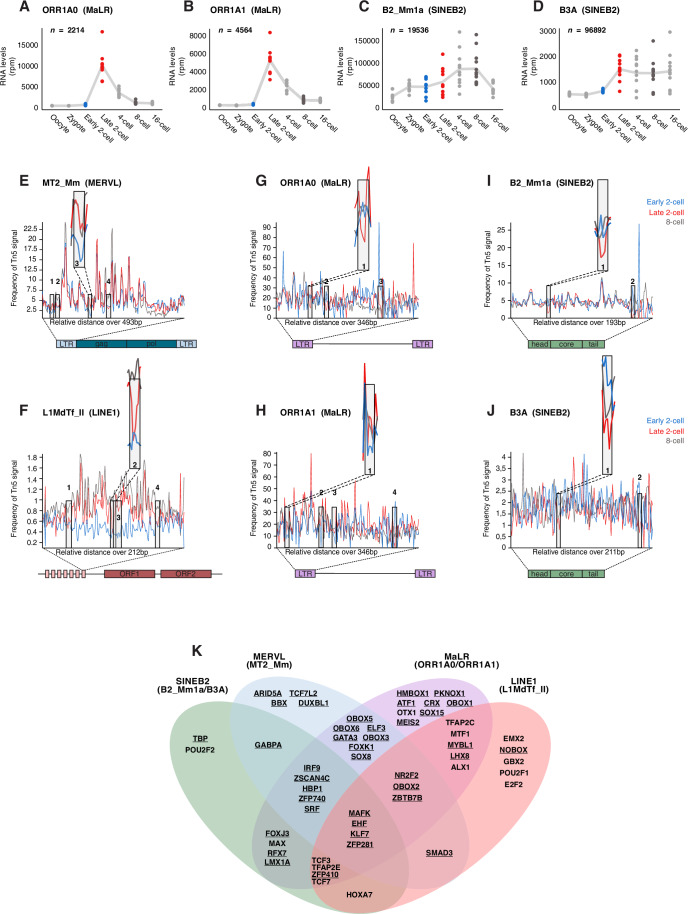
Footprinting analysis identifies 54 putative regulators of TE expression. (**A**–**D**) Expression levels of all ORR1A0 (**A**), ORR1A1 (**B**), B2_Mm1a (**C**), B3A (**D**) across preimplantation development analysed from (Oomen et al, 2025). Each dot represents the sum rpm of all insertions belonging to each family of TE per single embryo at the indicated stage. *n* is the number of insertions per group. The trend line connects the mean values across embryos for each stage. (**E**–**J**) Tn5 insertion frequency over full-length MT2_Mm LTRs (**E**), L1MdTf_II monomers (**F**), ORR1A0 LTRs (**G**), ORR1A1 LTRs (**H**), B2_Mm1a complete elements (**I**), B3A complete elements (**J**). Early 2-cell insertion frequency is displayed in blue, late 2-cell in red and 8-cell in brown. The consensus length according to Repbase is indicated for each TE. The inset shows a larger image of an example footprint for each TE. For each TE, the footprints are numbered from left to right, indicated on the graphs. The associated TFBS for each footprint are listed in Table EV1. For MT2_Mm, the highlighted footprint corresponds to the position of the binding site for DUX. (**K**) Venn diagram displaying the 54 candidate TFs obtained from footprint analysis and filtering for expression at the 2-cell stage for each TE family. The underlined TFs correspond to the 40 TFs that were chosen to be functionally tested.

**Figure EV1:**
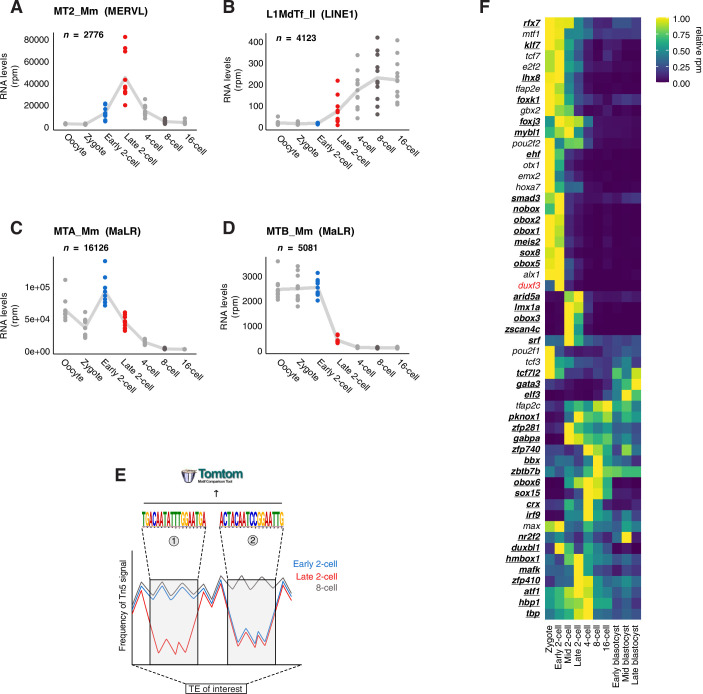
Footprinting analysis identifies 54 putative regulators of TE expression. (**A**–**D**) Expression levels of all MT2_Mm (**A**), L1MdTf_II monomers (**B**), MTA_Mm (**C**) and MTB_Mm (**D**) across preimplantation development. Each dot represents the sum rpm of all insertions belonging to each family of TE per single embryo at the indicated stage. *n* is the number of insertions per group. The trend line connects the mean values across embryos for each stage. (**E**) Schematic representation of the footprinting analysis. The coloured lines over the TE of interest represent the aggregated Tn5 insertion signal at the different stages. When a footprint is found by visual inspection to differ between stages, for example, the red line (late 2-cell stage) compared to the blue (early 2-cell stage) and grey (8-cell stage) lines at the same position of the TE, the sequence corresponding to those regions was extracted and subjected to motif search using the Tomtom tool of the MEME suite. (**F**) Heatmap showing the expression of the 54 candidate TFs (and *Duxf3*) across all the stages of preimplantation development. The values are the normalized counts centred on the row mean reanalysed from (Deng et al, 2014). TFs were ordered by hierarchical clustering. The TFs highlighted in bold and underlined correspond to the 40 TFs that were selected for functional studies.

In order to expand and characterise the repertoire of TFs regulating TEs at the beginning of mammalian development, we first searched for major TE families with abundant transcripts in early mouse embryos. The MERVL LTR *MT2_Mm* and LINE-1 ‘Tf’ (e.g. *L1MdTf_II*) family of retrotransposons are well known for their characteristic expression during ZGA and their role in early development (Peaston et al, 2004; Svoboda et al, 2004; Fadloun et al, 2013; Jachowicz et al, 2017). Thus, we first investigated the expression dynamics of additional families of TEs in mouse oocytes and embryos until the 16-cell stage, that is prior to the blastocyst stage when the first lineage allocation emerges. Specifically, we focused on MaLR and SINEs with temporal dynamics reflecting transcriptional activation concomitant with ZGA, similarly to MT2_Mm and L1MdTf_II expression dynamics (Fig. EV1A,B). We discarded TEs that are expressed in the oocyte, since maternally expressed transcripts can be inherited by the early embryo but do not necessarily reflect transcriptional activation in embryos. We used RNA-seq datasets generated with a newly developed SMART-Seq +5’ protocol that enables simultaneous quantification of gene expression and mapping of the transcription start site (TSS) of TEs in single embryos (Oomen et al, 2025). We identified ORR1A0 and ORR1A1 as two MaLR elements with similar developmental expression kinetics reflecting transcriptional activation at ZGA (Fig. 1A,B). These two MaLRs are the highest LTR-containing TEs transcribed at the 2-cell stage after MT2_Mm/MERVL (Peaston et al, 2004; Franke et al, 2017; Oomen et al, 2025). Their expression pattern is very similar to that of MERVL and differs from the MTA_Mm and MTB_Mm families of MaLR which are highly expressed in oocytes (Peaston et al, 2004; Franke et al, 2017; Yu et al, 2020) (Fig. EV1C,D). Likewise, from the SINE family, the SINE B2_Mm1a and SINE B3A families displayed a pattern of expression consistent with ZGA and high levels of expression (Fig. 1C,D). All these 4 TEs exhibited transcriptional activation at ZGA (Fig. 1A–D). Thus, for subsequent experiments, we decided to focus on the 2 MaLRs that exhibit similar expression dynamics to MT2_Mm (ORR1A0 and ORR1A1) and two additional TEs (SINEs B2_Mm1a and B3A), which are also activated during major ZGA but display different dynamics at later developmental stages.

To identify transcriptional regulators of these 4 TEs, we performed footprint analysis with ATAC-seq data from mouse embryos prior to, during and after major ZGA, specifically from early, late 2-cell stage and 8-cell embryos (Wu et al, 2016). A footprint typically emerges as a region of locally decreased sequencing read coverage, due to TF protection from cleavage by the Tn5 transposase, allowing the identification of sites bound by TFs with nucleotide resolution. As proof-of-principle, we also included MT2_Mm and L1MdTf_II in our analyses. We aligned Tn5 insertion signal to reference TE sequences that correspond to the length of the respective consensus sequence of each TE. We next performed a qualitative inspection of the footprints to identify regions with differential local depletion of signal relative to flanking regions at the early 2-cell stage, late 2-cell stage or both (Fig. EV1E). This analysis led to the identification of several footprints across all 6 TE families that were most prominent at the late 2-cell stage (Fig. 1E–J), indicative of changes in occupancy at the precise locations of the regulatory regions of these TEs at ZGA. For example, for MT2_Mm, we identified 2 footprints present at both the early and late 2-cell stages and 2 others exclusively at the early 2-cell stage, one of which corresponds to the known binding site of DUX at MT2_Mm (Hendrickson et al, 2017) (Fig. 1E, inset). Interestingly, despite their high sequence similarity (96% in their RepBase consensus sequences), ORR1A0 and ORR1A1 exhibited different footprints (Fig. 1G,H).

We next extracted the sequences corresponding to the footprints from the consensus sequences and subjected them to a motif analysis search using the Tomtom tool from the MEME suite. Tomtom identifies potential TF motifs from existing databases by ranking them based on estimated statistical significance of the match between query and target sequences (Gupta et al, 2007). We filtered this list of candidate TFs based on whether they are expressed at the 2-cell stage, using a publicly available RNA-seq dataset (Deng et al, 2014). This analysis led to the identification of 15 and 42 TF motifs in MaLRs footprints (ORR1A0 and ORR1A1, respectively); 16 and 9 in SINE B2 elements (B3A and B2_Mm1a, respectively); 33 in LINE L1 L1MdTf_II and 33 in MT2_Mm (Dataset EV1 and Table EV1). Some TFs have a primary and a secondary motif, their motif was present on either the forward or the reverse strand and they were present in different footprints within the same or different TEs. Thus, after grouping motifs across all footprints and strands, we obtained a list of 54 TFs (Fig. 1K), for which we further analysed their mRNA expression kinetics throughout pre-implantation development (Fig. EV1F). We also included DUX in this analysis, which is not included in the UniPROBE database that we used with the Tomtom tool, but is known to directly bind and activate MT2_Mm (Hendrickson et al, 2017; De Iaco et al, 2017; Whiddon et al, 2017). Transcripts from more than half of these 55 TFs (39) are present as early as the zygote stage (Fig. EV1F), suggesting that they are inherited as maternal transcripts. The remaining TFs (excepting ELF3), display mRNA level kinetics consistent with transcriptional activation at the early or mid 2-cell stage (Fig. EV1F). Perhaps unexpectedly, we find that many of the identified TF motifs show significant overlap between the 6 TE families, even amongst those that are evolutionary distinct families such as LINE1 and MaLRs (Fig. 1K). Importantly, our search revealed known characterised motifs, for example MT2_Mm presented binding sites for the OBOX family of transcription factors and for GATA3, which has the same binding motifs as GATA2 (Fig. 1K) (Ji et al, 2023; Ko and Engel 1993; Choi et al, 2017). Interestingly, some of these TFs, including for example ARID5A, CRX, OTX1 and OBOX2 were also identified in a similar footprinting analysis but focused on ZGA genes exclusively, instead of TEs (Bentsen et al, 2020). Thus, footprinting analysis of 6 different TE families expressed in early mouse embryos identified 54 potential regulators of TEs during preimplantation development.

### A selection of transcription factors expressed in early mouse embryos activates transcription of specific TE families in mouse embryonic stem cells

**Figure 2:**
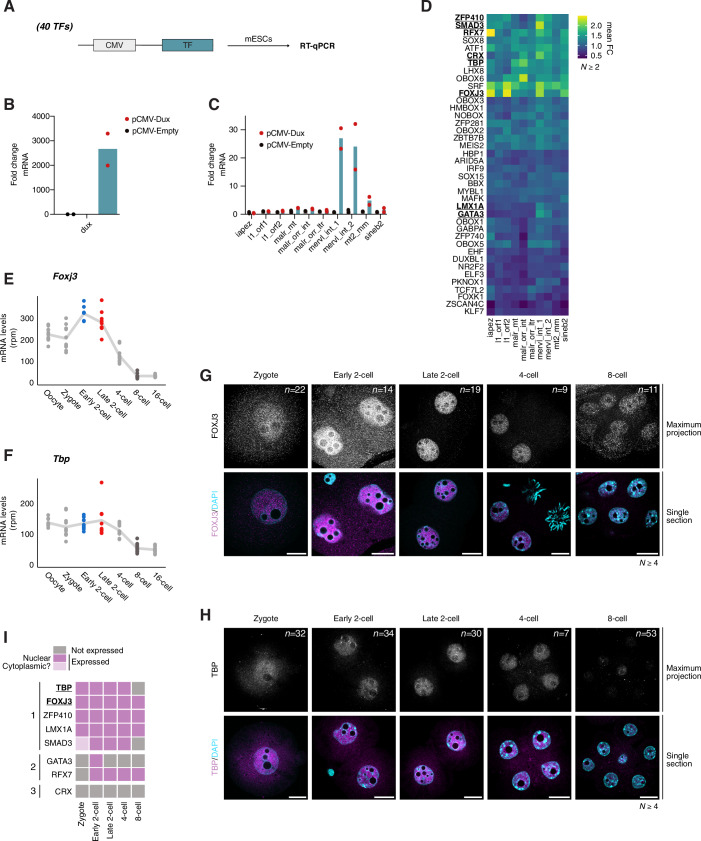
A selection of TFs is expressed during preimplantation development and activates specific TE families upon overexpression in ESCs. (**A**) Schematic representation of the experimental design used to test the capacity of 40 TFs to transcriptionally activate TEs in ESCs. (**B**, **C**) Fold change of *Dux* mRNA levels (**B**) and indicated TE families RNA levels (**C**) over no vector control by RT-qPCR. The bar represents the mean over two biological replicates, the black and red dots represent the mean over three technical replicates for each biological replicate of cell transfected with pCMV-Empty and pCMV-Dux, respectively. (**D**) Heatmap depicting the average fold change of at least two biological replicates of three technical replicates of the indicated TE families upon overexpression of the indicated TF by RT-qPCR. TFs were ordered by hierarchical clustering. The TFs highlighted in bold and underlined correspond to the 8 TFs that were selected for subsequent experiments. (**E**,** F**) Expression of *Foxj3* (**E**) and *Tbp* (**F**) during mouse preimplantation development. Each dot represents *Foxj3* or *Tbp* mRNA levels (rpm) in individual embryos at the indicated stages reanalysed from (Oomen et al, 2025). The trend line connects the mean values across embryos for each stage. (**G**,** H**) Representative images of FOXJ3 (**G**) and TBP (**H**) immunostainings at the indicated developmental stages. The intensities of the fluorescent signal are comparable as all embryos within each replicate were processed and acquired using the same conditions. Top images are maximum intensity projections, bottom are merged images with DAPI staining shown as single confocal sections. *n* is the total number of embryos analysed per stage. *N*, the number of independent replicates. Scale bars, 20 µm. (**I**). Heatmap showing a qualitative summary of the expression profiles of 8 candidate TFs at the protein level. Grey represents not expressed, pink nuclear localization, and light pink presumed cytoplasmic localization. TFs are ordered according to the three patterns of expression observed: 1-maternally inherited protein, 2-zygotic expression, and 3-virtually absent. The TFs highlighted in bold and underlined correspond to the 2 TFs that were selected for functional experiments in vivo. Source data are available online for this figure.

**Figure EV2:**
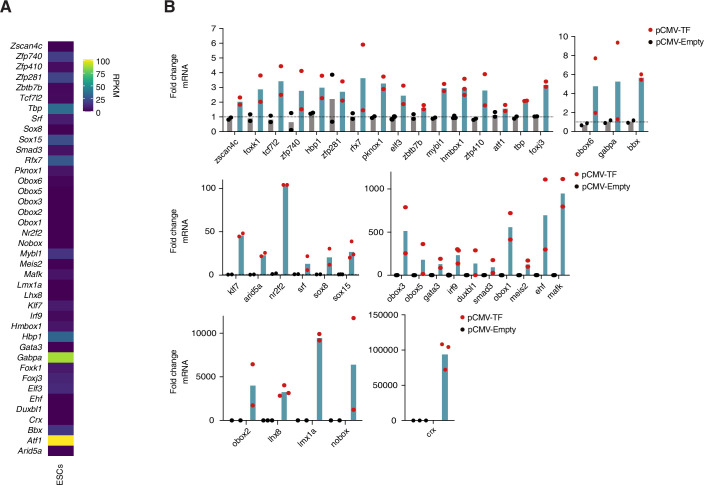
A selection of TFs activates specific TE families upon overexpression in ESCs. (**A**) Heatmap showing the expression of the 40 candidate TFs chosen for overexpression experiments in ESCs. The values are rpkm and derive from (Ishiuchi et al, 2015). (**B**) Fold change of the mRNA levels of the 40 TFs upon transfection of each of them over the corresponding empty-vector control by RT-qPCR. The bar represents the mean from two or three biological replicates, as indicated by the number of black and red dots, which represent the mean of three technical replicates for each biological replicate of cells transfected with pCMV-Empty and pCMV-TF, respectively. Source data are available online for this figure.

To directly address whether the above TFs can drive transcription of the selected TEs we first used mouse ESCs. This allowed us to screen a large group of TFs for their capacity to activate endogenous TEs, which is not feasible with the same throughput in mouse embryos. We decided to focus on 40 of the 54 potential candidates, based on expression profiles, quality of the motif match and novelty. In addition, most of these 40 transcription factors were expressed in ESCs, albeit at low levels (Fig. EV2A), except for ATF1 and GABPA. We cloned all 40 candidate TFs in a mammalian expression vector and transfected them individually in ESCs after which we assessed TE expression by RT-qPCR in at least 2 biological replicates with 3 technical replicates each (Fig. 2A). We verified that all TFs were expressed upon transfection by RT-qPCR (Fig. EV2B). For all TFs, we analysed the expression of the main TEs expressed in early mouse embryos that belong to the three major retrotransposon families (LTR, LINE1 and SINEB2) (Peaston et al, 2004; Oomen et al, 2025). Specifically, we investigated expression of IAPEZ, L1_orf1, L1_orf2, MaLR_MT, MaLR_ORR_int, MaLR_ORR_LTR, MERVL_int, MT2_Mm and SINEB2 using specific RT-qPCR primers. We verified that our experimental design is amenable for detection of potential changes in endogenous TE expression by using DUX as a positive control, which is known to activate MT2_Mm and MERVL upon ectopic expression in ESCs (Hendrickson et al, 2017), and which our experiments confirmed (Fig. 2B,C). Globally, we find several TFs that increased the transcription of TEs upon ectopic expression in ESCs (Fig. 2D). Interestingly, several of them activated more than one class of TEs (Fig. 2D). This is in line with our TFBS prediction in Fig. 1K. The transcriptional activation of TEs was largely consistent with the footprinting analysis, but we noted that some TFs activated TEs without having a predicted TFBS. This may be due to the fact that the ATAC-seq data used for the analysis is from mouse embryos and not from ESCs. Overall, 10 TFs provided interesting patterns of TE activation. FOXJ3, RFX7, TBP and SRF were amongst the TFs that activated several TEs most robustly (Fig. 2D). For example, FOXJ3 led to increased expression of IAPEZ, LINE1 ORF2, MERVL and SINE B2, which belong to very distinct TE families. Interestingly, SRF and TBP, which are general, ubiquitously expressed TFs also resulted in increased expression of most TEs (Fig. 2D), although we note that we cannot formally rule out indirect effects in these experiments. TBP activity was particularly clear on MaLRs, which was not predicted from the footprinting analysis. A possible explanation to this apparent discrepancy may be that binding motifs for some TFs in embryos may differ from those in the UniPROBE database. This is supported by data indicating that some TFs, such as OBOX are known to bind an extended motif in embryos, which is different from the motif sequence documented in TFBS databases (Ji et al, 2023). SRF, in contrast, led to stronger activation of MERVL, MT2_Mm, LINE1 ORF2 and IAPEZ (Fig. 2D). This is in line with recent work indicating that SRF regulates MERVL both in luciferase assays and in mouse embryos (Hermant et al, 2025). In contrast, other TFs were more restricted to specific TEs, for example SMAD3 and CRX led to activation of MERVL and OBOX6 of MaLR_ORR_int (Fig. 2D). LMX1A and GATA3 also led to activation of MERVL, albeit to a lesser extent compared to SMAD3 and CRX (Fig. 2D). Expression of the zinc finger protein ZFP410 resulted in the rather promiscuous activation of TEs, albeit to a lesser extent (Fig. 2D). Because OBOX6 and SRF have been studied elsewhere (Cheng et al, 2007; Ji et al, 2023; Hermant et al, 2025), we focused on the 8 remaining TFs for subsequent experiments.

**Figure EV3:**
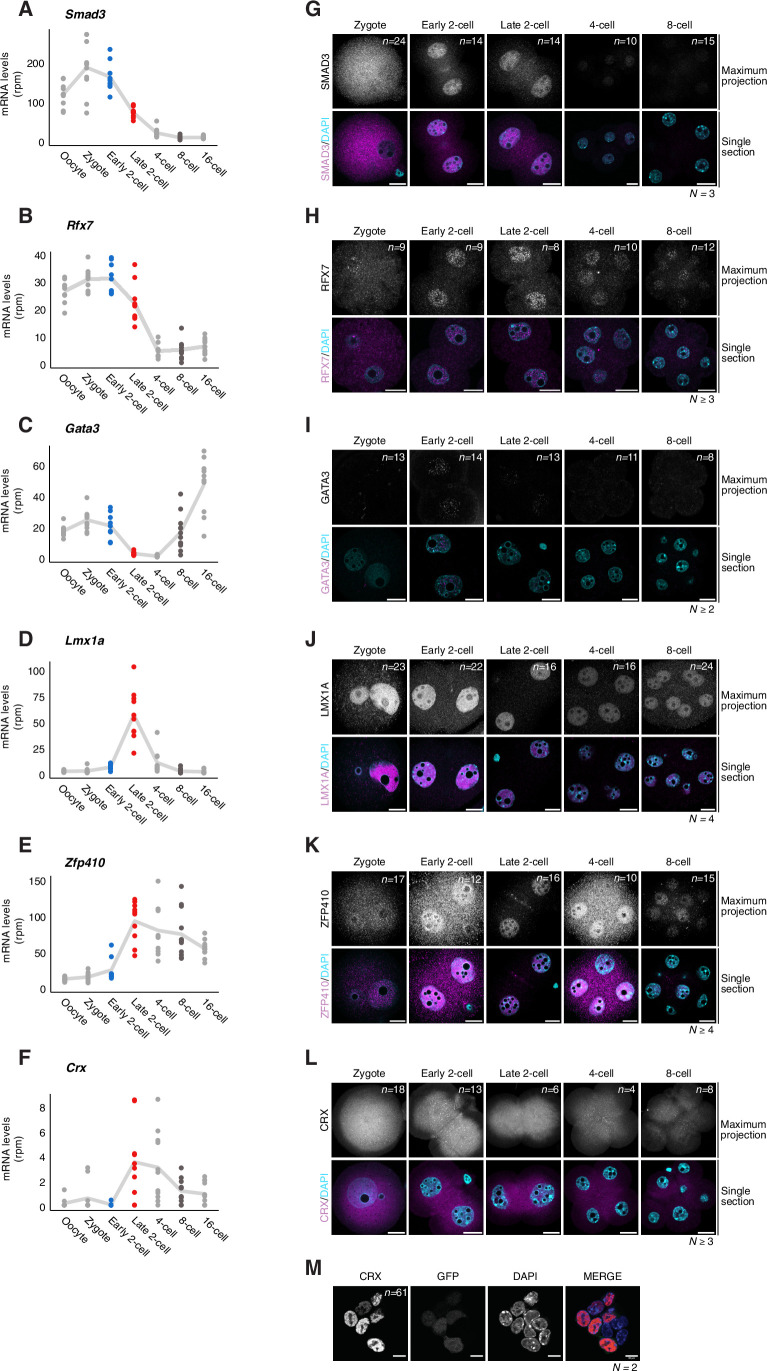
A selection of TFs is expressed during preimplantation development. (**A**–**F**) Expression of *Smad3* (**A**), *Rfx7* (**B**), *Gata3* (**C**), *Lmx1a* (**D**), *Zfp410* (**E**) and *Crx* (**F**) during mouse preimplantation development. Each dot represents the TF mRNA levels (rpm) in individual embryos at the indicated stages analysed from (Oomen et al, 2025). The trend line connects the mean values across embryos for each stage. (**G**–**L**) Representative images of SMAD3 (**G**), RFX7 (**H**), GATA3 (**I**), LMX1A (**J**), ZFP410 (**K**) and CRX (**L**) immunostainings at the indicated developmental stage. All embryos within each replicate were processed and acquired using the same conditions, hence the intensity of the fluorescent signal is comparable between all embryos. Top images are maximum intensity projection, bottom are merged images with DAPI staining shown as single confocal sections. *n* is the total number of embryos analysed per stage. *N*, the number of independent replicates. Scale bars, 20 µm. (**M**) Representative image of CRX immunostaining and GFP fluorescence (as a positive control for successful transfection) upon CRX overexpression in ESCs. *n* is the number of cells that were displaying both GFP and CRX signal. *N*, the number of independent replicates. Scale bars, 10 µm. Source data are available online for this figure.

First, we examined mRNA expression levels of these 8 TFs during mouse pre-implantation development using public datasets (Oomen et al, 2025). We find that all 8 TFs are expressed in early embryos, although with different developmental dynamics. Five of them (*Foxj3*, *Tbp*, *Smad3*, *Rfx7* and *Gata3*) are present as maternal transcripts, with high levels already in unfertilised oocytes (Figs. 2E,F and EV3A–C). The abundance of mRNAs for most of these maternal TFs, excepting for *Gata3*, decreases after fertilisation, albeit at different times: *Smad3* and *Rfx7* are practically undetectable at the 4-cell stage, *Foxj3* at the 8-cell stage and *Tbp* mRNA levels persist throughout pre-implantation stages (Figs. 2E,F and EV3A–C). While most of these TFs have not been studied in the oocyte and early embryonic development, the pattern of expression of TBP is consistent with previous literature in oocytes (Gazdag et al, 2007, 2009). The other three TFs, *Lmx1a*, *Zfp410* and *Crx* show a typical major ZGA profile with highest mRNA abundance at the late 2-cell stage (Fig. EV3D–F). Because mRNA and protein abundance are often uncoupled in early embryogenesis, we examined protein levels of each of these 8 TFs by immunofluorescence (Figs. 2G,H and EV3G–L). Indeed, we find that CRX is not detectable in embryos between the zygote and the 8-cell stage (Fig. EV3L). This was not due to a technical issue of the antibody, since we detected nuclear CRX enrichment upon overexpression in ESCs (Fig. EV3M). We also detected only low levels of GATA3, which displayed nuclear localisation in early and late 2-cell stage embryos but was undetectable at all other stages analysed (Fig. EV3I). Similarly, SMAD3 localised transiently in nuclei of early and late 2-cell stage embryos and the levels of the protein decreased thereafter (Fig. EV3G). All other TFs were expressed throughout all the stages analysed (Figs. 2G,H and EV3H,J,K). RFX7 and ZFP410 showed a strong, clear nuclear localisation from the early 2-cell stage (Fig. EV3H,K). Lastly, FOXJ3 and TBP localised to the nuclei of mouse embryos at all stages analysed, from the late zygote to the 8-cell stage (Fig. 2G,H). Thus, CRX aside, all our candidate TFs are expressed at the protein level in early mouse embryos, albeit with slightly different temporal dynamics in their nuclear localisation (Fig. 2I).

Considering that FOXJ3 led to increase SINEB2 and LINE L1 expression in ESCs and that TBP expression led to very clear induction of MaLR transcripts (Fig. 2D), we focused on these two TFs for subsequent functional experiments in vivo. This is both because these TFs have not previously been associated with TE regulation, and because the regulation of these TEs is less well understood.

### Loss of TBP impairs transcription during major ZGA

**Figure 3:**
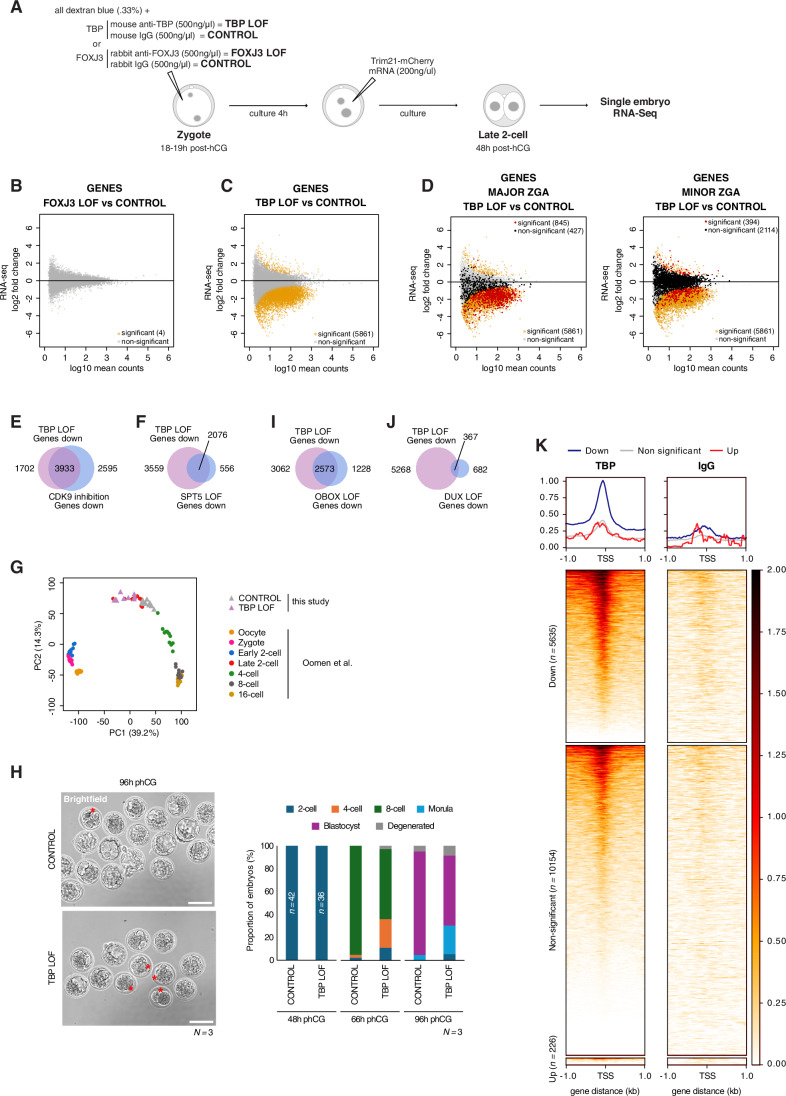
Loss of TBP impairs ZGA and delays developmental progression. (**A**) Schematic representation of the experimental design used to acutely deplete TBP (TBP LOF) and FOXJ3 (FOXJ3 LOF) in embryos followed by single embryo RNA-sequencing. (**B**,** C**) MA plots comparing log2 fold change in FOXJ3 LOF versus CONTROL (**B**) and TBP LOF versus CONTROL (**C**) embryos against log10 RNA-seq mean counts. Differentially expressed genes are labelled in orange (padj < 0.05) and non-differentially expressed genes are displayed in grey. (**D**) Same MA plot as in (**C**) but with major ZGA (left) or minor ZGA (right) from the DBTMEE database (Park et al, 2015) labelled in red when significant, non-significant in black. (**E**,** F**) Venn diagram displaying the overlap between down-regulated genes (padj < 0.05) upon TBP LOF relative to CONTROL embryos and upon CDK9 inhibition relative to control (**E**) or upon SPT5 Trim-Away relative to control (**F**) (Abe et al, 2022). (**G**) Principal component analysis (PCA) of the transcriptional profiles of TBP LOF and CONTROL embryos in comparison with wild-type non-manipulated embryos from oocytes to 16-cell stage (Oomen et al, 2025). Each point corresponds to an individual embryo, the conditions are displayed by the indicated colour code. Circles are wild-type embryos (Oomen et al, 2025). Triangles are embryos from this study. The variance explained (percentage) is indicated along the PC1 and PC2 axes. (**H**) Left: brightfield representative images at 96 h post-hCG of TBP LOF and CONTROL embryos. *N* is the number of biological replicates. Red asterisks highlight delayed embryos. Scale bars, 100 µm. Right: Developmental progression (in percentage) of CONTROL and TBP LOF embryos at 48 h, 66 h and 96 h post-hCG. The numbers in the bar plot at 48 h are the total number of embryos for each condition. *N* is the number of biological replicates. (**I**,** J**) Venn diagram displaying the overlap between down-regulated genes (padj < 0.05) upon TBP LOF relative to CONTROL embryos and upon OBOX knock-out relative to control (**I**) (Ji et al, 2023) or upon DUX knock-down relative to control (**J**) (Hermant et al, 2025). (**K**) Signal aggregate plots and heatmaps of TBP enrichment (left) or IgG control (right) from pooled CUT&Tag replicates (at the late 2-cell stage) over the TSS of down-regulated (padj < 0.05), non-significant and up-regulated (padj < 0.05) genes upon TBP LOF relative to CONTROL embryos. *n* is the number of genes per category. Source data are available online for this figure.

**Figure EV4:**
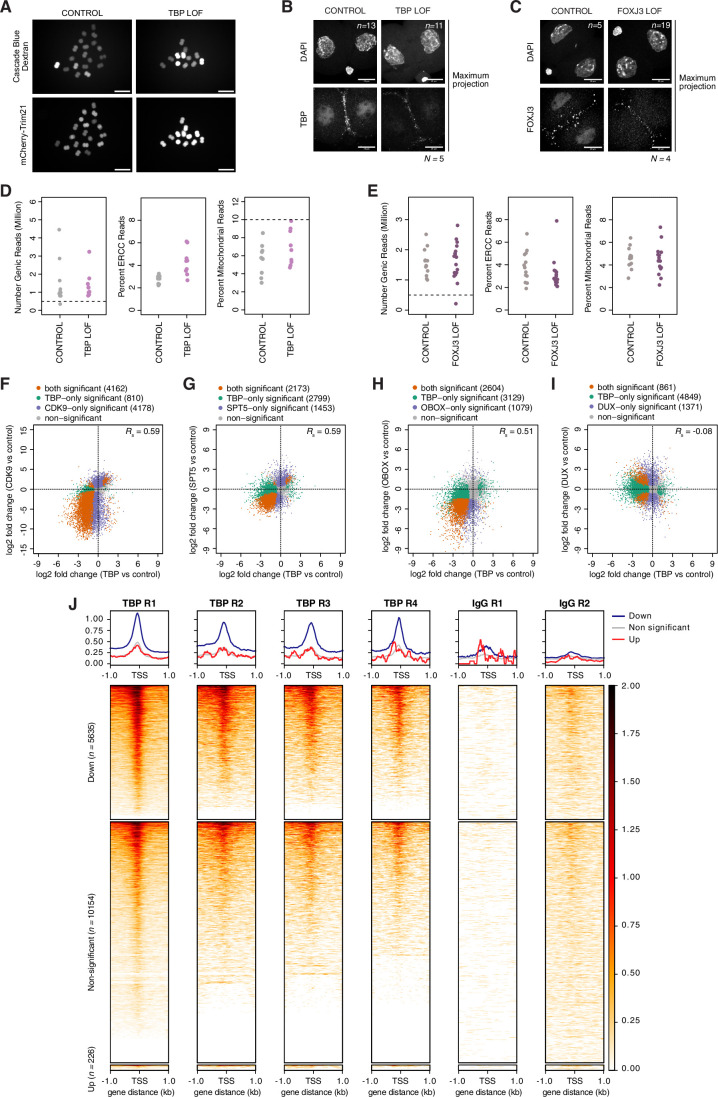
Loss of TBP impairs ZGA and delays developmental progression. (**A**) Representative images of late 2-cell stage embryos after sequential microinjection with TBP antibody or IgG antibody together with cascade blue-coupled dextran and mCherry-Trim21. All embryos were examined for cascade blue and mCherry fluorescence in all the experiments performed throughout the manuscript. Scale bar, 200 µm. (**B**,** C**) Representative images of TBP (**B**) and FOXJ3 (**C**) immunostainings following acute protein depletion by Trim-Away. Left images are from either TBP or FOXJ3 LOF, right images correspond to the respective CONTROL embryos (see Fig. 3A). All images shown are maximum intensity projections. Top is DAPI signal, bottom is either TBP or FOXJ3 staining. *n* is the total number of embryos analysed per condition. *N*, the number of independent replicates. Scale bars, 20 µm. (**D**,** E**) Dot plots showing single embryo RNA-sequencing quality controls of TBP LOF and CONTROL embryos (**D**), and FOXJ3 LOF and CONTROL embryos (**E**). The number of genic reads, the percentage of ERCC reads and the percentage of reads mapping to mitochondrial genes in embryos are shown. Each dot is a single embryo, the dashed lines represent the thresholds applied. (**F**–**I**) Scatterplots of RNA-seq log2 fold change differences between CDK9 inhibition versus control (**F**) (Abe et al, 2022), SPT5 Trim-Away versus control (**G**) (Abe et al, 2022), OBOX knock-out versus control (**H**) (Ji et al, 2023) and DUX knock-down versus control (**I**) (Hermant et al, 2025) against TBP LOF relative to CONTROL. Genes significantly differentially expressed in TBP LOF only are depicted in green, those in CDK9, SPT5 or DUX LOF only are displayed in purple, and differentially expressed genes in both conditions are displayed in orange. Spearman’s correlations (*R*_s_) are indicated. (**J**) Signal aggregate plots and heatmaps of TBP enrichment at the late 2-cell stage from the four individual CUT&Tag replicates or IgG control from two individual replicates over the TSS of down-regulated (padj < 0.05), non-significant and up-regulated (padj < 0.05) genes upon TBP LOF relative to CONTROL embryos. *n* is the number of genes per category indicated. Source data are available online for this figure.

To address whether TBP and/or FOXJ3 regulate TEs in vivo in mouse embryos, we performed loss-of-function (LOF) experiments followed by single embryo RNA-seq using Smart-seq+5’ (Oomen et al, 2025). Because both TBP and FOXJ3 are maternally inherited, we used an established acute protein depletion method for mouse embryos, Trim-Away (Clift et al, 2017). Trim-Away triggers protein degradation by coupling antibodies against the protein to be targeted to the TRIM21 degradation pathway (Clift et al, 2017). We therefore microinjected anti-TBP or anti-FOXJ3 antibodies together with mRNA for mCherry-Trim21 into mouse zygotes or the corresponding IgG antibodies as negative controls (Clift et al, 2017) (Fig. 3A). Cascade blue-coupled dextran was used as microinjection control for the antibodies and mCherry fluorescence confirmed expression of TRIM21 in microinjected embryos (Fig. EV4A). This approach led to depletion of TBP and FOXJ3 at the 2-cell stage, compared to their corresponding IgG controls (Fig. EV4B,C). We collected 49 embryos in total for all 4 conditions for single embryo RNA-seq at the late 2-cell stage. Following standard quality control metrics (Fig. EV4D,E), we retained and analysed 47 embryos in total (9 TBP LOF and 9 corresponding controls; 16 FOXJ3 LOF and 13 corresponding controls). Differential gene expression analysis revealed minimum changes in gene expression upon FOXJ3 depletion, with only 4 genes significantly down-regulated (*Letm1*, *Ino80b*, *Gm20274, 2310039H08Rik*) (Fig. 3B and Dataset EV2). In contrast, depletion of TBP led to a major change in the transcriptome of 2-cell stage embryos, with 5861 genes significantly affected, of which most (96%; 5635/5861) were down-regulated (Fig. 3C and Dataset EV2). These findings are in line with the known role of TBP as a general transcription factor (Butler and Kadonaga 2002) and indicate a major role for TBP in regulating transcription in mouse embryos at the 2-cell stage. TBP had a considerable effect on major ZGA genes: of the 1272 genes categorised as ‘major ZGA genes’ (Park et al, 2015) and captured in our differential expression analysis, ~66% (*n* = 840 genes) were significantly down-regulated upon TBP depletion (5 were significantly up-regulated), but only ~13% of ‘minor ZGA genes’ (Park et al, 2015) (332 out of 2508) were down-regulated (62 were significantly up-regulated) (Fig. 3D and Dataset EV2). Of note, some well-known ‘2C’ markers of ZGA such as *Zscan4a-f*, *Eif4e3* or *Zfp352* were not affected (Dataset EV2).

In addition, consistently with a major role for TBP in mediating RNA Polymerase II transcription, there was a large overlap between the down-regulated genes in TBP LOF and genes which depend on CDK9 activity, a component of p-TEFB and a major regulator of NELF activity (Chen et al, 2018). CDK9 plays additional roles in RNA Polymerase II elongation through the phosphorylation of the SPT5 subunit of DSIF and the Ser 2 residue of the CTD (Kwak and Lis, 2013). Of the 5635 down-regulated genes in TBP LOF embryos, 3933 (~70%) are also dependent on CDK9 function (Figs. 3E and EV4F) (Abe et al, 2022), and out of the 6528 genes affected upon CDK9 inhibition (Abe et al, 2022), 60% are dependent on TBP (Figs. 3E and EV4F). Likewise, most genes (79%) whose transcription depends upon SPT5 at the 2-cell stage (Abe et al, 2022) were also down-regulated upon TBP LOF (2076 genes out of 2632) (Figs. 3F and EV4G). These data are consistent with earlier work indicating that CDK9 and SPT5 are key regulators of global transcription during ZGA in mouse embryos (Abe et al, 2022) and highlight the role of TBP in this process. Principal component analysis (PCA) of TBP LOF and control embryos together with non-manipulated, wild-type embryos (Oomen et al, 2025), indicated that embryos depleted of TBP were shifted along the axis of PC1 towards the earlier developmental stages relative to control embryos (Fig. 3G). This suggests that the transcriptional changes elicited upon TBP depletion were accompanied by a developmental delay. Accordingly, while TBP depletion did not completely prevent development till the blastocyst stage, we observed a delay in developmental progression upon Trim-Away for TBP (Fig. 3H). Namely, the division from the 4- to the 8-cell stage was delayed, as was blastocyst formation, but we did not observe an arrest at the 2-cell stage (Fig. 3H). While these observations are in line with the phenotype of TBP knock-out mice, which survive until the blastocyst stage (Martianov et al, 2002), we cannot rule out the presence of residual TBP protein and/or a transient effect of protein depletion by Trim-Away in our experimental conditions. However, the extent of the overlap of genes downregulated in SPT5 loss of function, which display a 2-cell arrest (Abe et al, 2022), suggests that specific genes, rather than a global transcriptional defect may be involved in the 2-cell arrest phenotype. To address this possibility, we compared transcriptome changes upon TBP loss with those emerging upon depletion of TFs known to regulate ZGA in mouse embryos: OBOX and DUX (Hendrickson et al, 2017; De Iaco et al, 2017, 2020; Whiddon et al, 2017; Hermant et al, 2025; Ji et al, 2023). A similar analysis of public datasets indicated that loss of OBOX led to the down-regulation of 3801 genes, out of which 2573 are down-regulated upon TBP-loss (Figs. 3I and EV4H). Likewise, depletion of DUX resulted in 1049 down-regulated genes of which 367 were also down-regulated in TBP LOF embryos (Figs. 3J and EV4I). Thus, while the extent of the transcriptional changes elicited upon TBP loss is larger compared to DUX and OBOX, probably due to its nature as general transcription factor, these changes do not prevent division to the 4-cell stage. In comparison, depletion of single OBOX TFs or DUX does not prevent developmental progression. Instead, only the depletion of a combination of TFs, or of maternal and zygotic *Obox* genes (*Obox1/2/3/4/5/7*) do (De Iaco et al, 2020; Ji et al, 2023; Guo et al, 2024). Altogether, this suggests that rather than a global transcriptional defect, it is specific genes that are important for the progression beyond the 2-cell stage. In agreement with this interpretation, we find that well-known ZGA markers are unaffected upon TBP loss.

We next addressed if TBP regulates its target genes by performing CUT&Tag for TBP in wild-type late 2-cell stage embryos. We performed 4 replicates for TBP and 2 for the IgG controls, which gave consistent results (Fig. EV4J) and thus we pooled the replicates for subsequent analyses. Amongst genes, which are expressed, we find that the most down-regulated ones are bound by TBP ± 1 kb of their TSSs (Fig. 3K), indicating that TBP regulates these genes in mouse embryos. However, TBP also binds a subset of genes, which, while expressed, are not significantly affected upon TBP loss (Fig. 3K). Importantly, we did not detect TBP binding to the up-regulated genes (*n* = 226), suggesting that the effect on these genes may be indirect (Fig. 3K). We conclude that TBP, in contrast to FOXJ3, contributes to the transcriptional programme of RNA Polymerase II at ZGA by binding to and regulating gene expression of about half of major ZGA genes and that this transcriptional defect does not prevent embryos from further development, but only causes a delay.

### TBP binds to and regulates MaLRs in early mouse embryos

**Figure 4:**
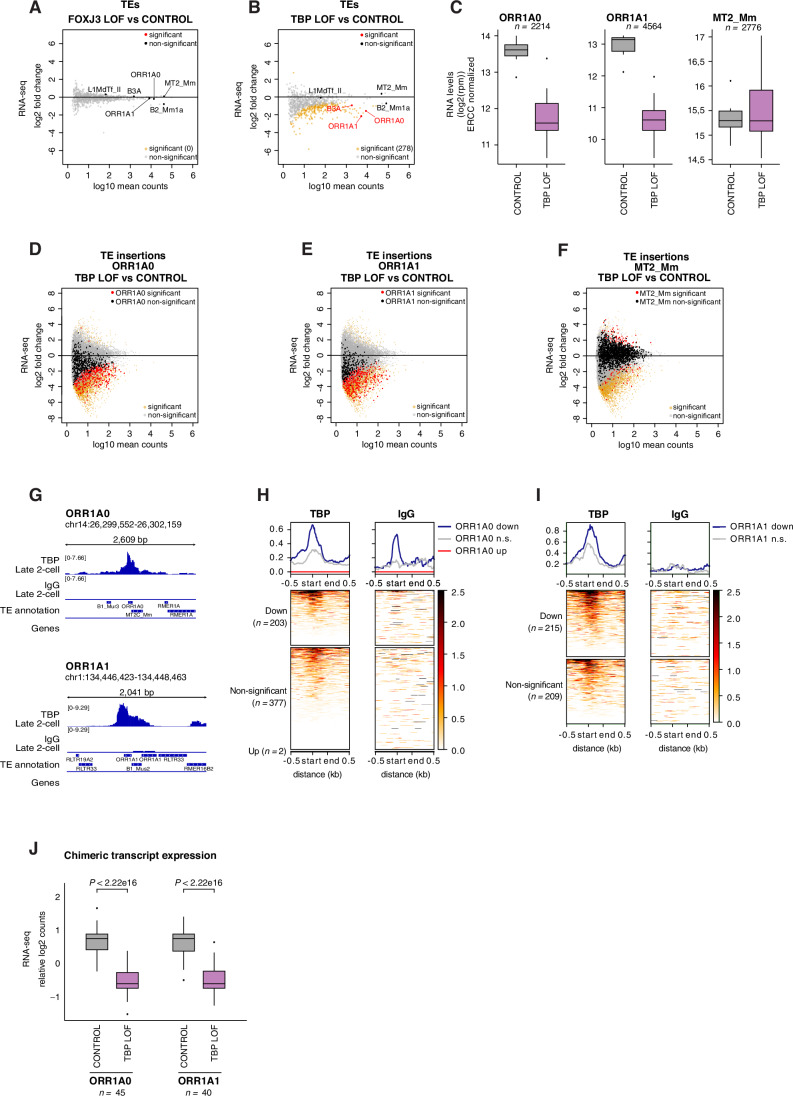
TBP binds to and regulates MaLR during preimplantation development. (**A**,** B**) MA plots comparing log2 fold change in FOXJ3 LOF and CONTROL (**A**) and TBP LOF and CONTROL (**B**) embryos against log10 RNA-seq mean counts. Differentially expressed TEs are labelled in orange (padj < 0.05) and non-differentially expressed TEs are displayed in grey. Non-significant TEs of interest are labelled in black, significantly differentially expressed TEs of interest are labelled in red. (**C**) Expression of ORR1A0 (left), ORR1A1 (middle) and MT2_Mm (right) in CONTROL and TBP LOF embryos. Boxplots show the median and the interquartile range (IQR) of ORR1A0, ORR1A1 and MT2_Mm-derived rpm normalized by ERCC (External RNA Controls Consortium) across all embryos. Whiskers display the highest and lowest value within 1.5 times of the interquartile range. *n* is the number of insertions per group. (**D**–**F**) MA plots comparing log2 fold change in TBP LOF and CONTROL embryos against log10 RNA-seq mean counts. Differentially expressed individual TE insertions are displayed in orange (padj < 0.05) and non-differentially expressed individual TE insertions are displayed in grey. Non-significant individual ORR1A0 insertions (**D**), ORR1A1 insertions (**E**) and MT2_Mm insertions (**F**) are labelled in black, significantly differentially expressed individual insertions of the same family are labelled in red. (**G**) Example genomic regions on the IGV browser showing merged replicates CUT&Tag signal on individual inserts of ORR1A0 (top) and ORR1A1 (bottom). (**H**,** I**) Signal aggregate plots and heatmaps of TBP enrichment (left) or IgG control (right) from pooled CUT&Tag replicates (at the late 2-cell stage) over the downregulated (padj < 0.05), non-significant and upregulated (padj < 0.05) ORR1A0 (**H**), ORR1A1 (**I**) LTRs upon TBP LOF relative to CONTROL embryos. Start and end refer to the position of the LTR (ORR1A0, ORR1A1). *n* is the number of insertions per category indicated. (**J**) Boxplot showing the median and interquartile ranges of the expression of chimeric TE-genes driven by ORR1A0 and ORR1A1 in CONTROL and TBP LOF embryos. Values are log2-normalized counts for each condition centred on chimeric TE mean. Whiskers are the highest and lowest value within 1.5 times the IQR. *n* is the number of chimeric TE-gene transcripts initiated in ORR1A0 or ORR1A1. Statistical significance was computed using t-tests. Source data are available online for this figure.

Our results above suggest a potential role for TBP and FOXJ3 in regulating TE expression and thus we next asked if TEs are de-regulated in TBP or FOXJ3 LOF embryos. Similar to its effect on the expression of genes, FOXJ3 depletion had no detectable effect on TE expression, with no TE significantly down-regulated in embryos after FOXJ3 Trim-Away (Fig. 4A and Dataset EV3). In contrast, depletion of TBP resulted in 278 differentially expressed TE families (Fig. 4B and Dataset EV3). Strikingly, except for one DNA transposon family (Eulor6D) (Dataset EV3), all affected TEs were down-regulated, including some members of the LINE1 family such as L1MEa, L1MCb or L1M8 (Dataset EV3). Notably, these LINE1 subfamilies are old eutherian TEs, older than the mouse specific L1MdTf_II, which was not affected. In addition, several rodent-specific SINEB1 elements, such as B1Mus1, B1F, B1_Mur1 or B1_Mm were significantly downregulated in the absence of TBP, as well as SINEB2 B3A family, but not B2_Mm1a (Fig. 4B and Dataset EV3). Importantly, many of the down-regulated TEs belong to the MaLR subclass, specifically from the ORR1A0, ORR1A1, ORR1A2, ORR1A3 families (Dataset EV3). Amongst them, ORR1A0 and ORR1A1 were activated upon TBP overexpression in mouse ESC (Fig. 2D) and were down-regulated 3- and 5-fold, respectively, in 2-cell embryos depleted of TBP (Fig. 4B,C). The effect on these MaLRs was specific, since TBP depletion did not lead to down-regulation of non-MaLR ERVLs such as MT2_Mm and MERVL (Fig. 4B,C and Dataset EV3), and oocyte-specific MaLRs such as MTA_Mm and MTB_Mm were also unaffected (Dataset EV3).

To address the extent to which TBP regulates these two families of MaLRs, we performed an analysis at the insertion level using TElocal (Jin et al, 2015). ORR1A0 and ORR1A1 are present in the mouse genome in 2214 and 4565 insertions, respectively. However, we find that only 582 ORR1A0 and 424 ORR1A1 insertions are expressed. Differential expression analyses indicated that 35% of the expressed ORR1A0 insertions at the 2-cell stage (203 of 582) were significantly downregulated upon TBP LOF (Fig. 4D). Two insertions were significantly up-regulated (Fig. 4D). Likewise, half of the expressed ORR1A1 insertions (215 of 424) were significantly downregulated in TBP LOF embryos (Fig. 4E). In contrast, a similar analysis for MT2_Mm indicated that only ~3% of the expressed MT2_Mm (58 of 1841 insertions) were downregulated (Fig. 4F). A slightly higher number (76 out of 1841) were up-regulated (Fig. 4F), potentially reflecting either indirect effects or a slightly delayed developmental stage in TBP LOF embryos. Thus, these results indicate that TBP regulates the expression of specific TE families in vivo.

**Figure EV5:**
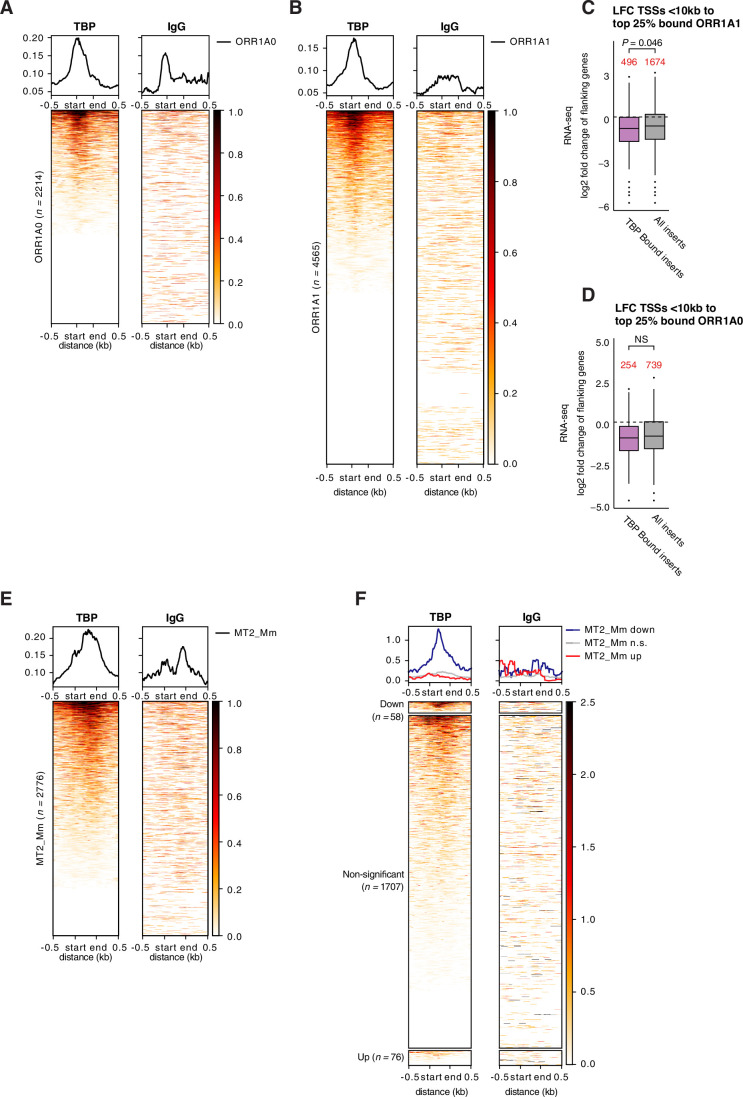
TBP binds to and regulate MaLR during preimplantation development. (**A**,** B**) Signal aggregate plots and heatmaps of TBP enrichment (left) or IgG control (right) from pooled CUT&Tag replicates (at the late 2-cell stage) over all ORR1A0 (**A**), ORR1A1 (**B**) insertions. Start and end refer to the position of the LTR (ORR1A0, ORR1A1). *n* is the number of insertions per TE family indicated. (**C**,** D**) Boxplots showing the median and interquartile ranges of the log2 fold change (LFC) of genes with their TSS positioned within 10 kb of bound inserts of ORR1A1 (**C**) and ORR1A0 (**D**). Inserts are qualified as “bound” inserts when they belong to the top 25% inserts based on CUT&Tag heatmap signal shown in (**A**) and (**B**). Whiskers are the highest and lowest value within 1.5 times the IQR. The numbers in red correspond to the number of genes per category as indicated. Statistical significance was computed using t-test. NS, non-significant. (**E**) Signal aggregate plots and heatmap of TBP enrichment (left) or IgG control (right) from pooled CUT&Tag replicates (at the late 2-cell stage) over all MT2_Mm insertions. Start and end refer to the position of the LTR. *n* is the number of insertions per TE family indicated. (**F**) Signal aggregate plot and heatmap of TBP enrichment (left) or IgG control (right) from pooled CUT&Tag replicates (at the late 2-cell stage) over the downregulated (padj < 0.05), non-significant and upregulated (padj < 0.05) MT2_Mm LTRs upon TBP LOF relative to CONTROL embryos. Start and end refer to the position of the LTR. *n* is the number of insertions per category indicated.

Next, we addressed whether TBP regulates TE expression through direct binding at their LTRs. For this, we analysed our TBP CUT&Tag 2-cell stage datasets and asked whether ORR1A0 and ORR1A1 insertions are bound by TBP. Indeed, we observed binding of TBP to a subset of ORR1A0 and ORR1A1 insertions compared to an IgG control (Fig. EV5A,B), which was also visible in representative IGV browser snapshots (Fig. 4G). We next asked whether the differentially expressed ORR1A0 and ORR1A1 TE insertions are bound by TBP. This analysis revealed that TBP indeed binds to a fraction of down-regulated ORR1A0 and ORR1A1 insertions (Fig. 4H,I). However, we note that some ORR1A0 and ORR1A1 insertions that are not transcriptionally affected by loss of TBP are also bound by TBP (Fig. 4H,I). Thus, a subset of down-regulated MaLR insertions is bound by TBP, indicating that TBP binds to and regulates these TEs.

Having shown that TBP binds to and regulates the transcription of a subset of MaLR insertions, we then asked whether MaLR transcription at the onset of development is relevant to the biology of the host. First, we explored whether ORR1A0 and ORR1A1 may regulate neighbouring genes. For this, we asked whether the expression of genes in proximity to TBP-bound ORR1A0/A1 insertions is affected upon TBP loss. Changes in the expression of genes whose TSS is within 10 kb of a TBP-bound ORR1A1 insertion are significantly larger than those genes in proximity to any ORR1A1 insertion upon TBP LOF (Fig. EV5C). We observed a similar tendency for TBP-bound ORR1A0 insertions, albeit not-statistically significant at the 5% significance level (Fig. EV5D). These data together suggest that TBP-bound ORR1A could affect the expression levels of nearby genes. Second, to further explore a function for ORR1A, we asked whether ORR1A1 and ORR1A0 can initiate genic transcription in embryos. We find that both ORR1A1 and ORR1A0 form chimeric transcripts at the 2-cell stage (Dataset EV4), indicating that they function as alternative promoters of host genes during ZGA. Importantly, TBP depletion led to a significant down-regulation of ORR1A0 and ORR1A1-driven chimeric transcripts (Fig. 4J). Altogether, these data imply that ORR1A0 and ORR1A1 regulate gene expression in mouse embryos and this role is at least in part dependent on TBP.

We showed that TBP LOF does not affect MT2_Mm expression despite the large number of mis-regulated ZGA genes upon TBP loss, suggesting that TBP may regulate ZGA independently of MT2_Mm. Considering the suggested prominent role of MT2_Mm in regulating ZGA, this prompted us to investigate these findings further. Our CUT&Tag analysis indicate that TBP is enriched at a subset of MT2_Mm insertions at the late 2-cell stage (Fig. EV5E), including some that are down-regulated upon TBP loss (Fig. EV5F). This suggests that TBP binds to and regulates a small fraction of MT2_Mm insertions, and that TBP regulation of ZGA for the most part acts independently of MT2_Mm.

## Discussion

Here, through in silico analyses using ATAC-seq footprinting and TFBS information from mouse embryos, we have identified 54 potential activators of TEs, which we verified through overexpression in stem cells. While further work is required for characterisation of many of these TFs, our work expands the repertoire of TFs involved in TE regulation. Our work focuses on TE regulators, given the increasingly recognised role of TE transcription after fertilisation, strengthening earlier work on the identification of TFs bound to the promoters of ZGA genes in mouse and human pre-implantation embryos (Bentsen et al, 2020).

Our targeted TF activity screening based on ectopic expression of TF candidates in mouse ESCs reveals potentially interesting biology. For example, none of the TFs studied elicited a transcriptional effect of the magnitude of DUX. This may be due to the pioneer transcriptional activity of DUX (Choi et al, 2016; Eidahl et al, 2016) which could suggest that none of the other TFs tested have pioneer activity. Additionally, most TFs increased transcription of several families of TEs. While we cannot rule out that this is due to potential indirect effects of TF expression, these observations suggest that single TFs can regulate several classes of TEs and that some TEs may be co-regulated. This interpretation is in line with our prediction of TFBS based on footprinting data in embryos, for which we find many TFs shared across evolutionary distant TE families.

Interestingly, even though the effect of TBP expression on MaLR in ESCs is mild, removal of TBP in embryos resulted in a very strong reduction in MaLRs transcription. The limited action of TBP in ESCs may be due to low overexpression efficiency compared to endogenous TBP levels in these cells, and/or reflect a more compacted chromatin state that is not permissive for TE induction, compared to early embryos. Acute depletion of TBP in mouse ESCs has no effect on gene expression and potential effects on TE expression have not been studied (Kwan et al, 2023). In future studies, an analysis of TBP binding to TEs could clarify the potential role for TBP in mESCs. In contrast, we observed a large transcriptional phenotype in embryos at the 2-cell stage, suggesting that TBP may be particularly important for the massive gene activation occurring during ZGA. This is in line with earlier work showing that zygotic depletion of TBP leads to an almost complete reduction of UTP analogue incorporation by the blastocyst stage (Martianov et al, 2002). In contrast to TBP, depletion of FOXJ3, which we also predicted as a TF regulating MaLRs, resulted in practically no changes in gene or TE expression in mouse embryos. It is possible that other factors compensate for FOXJ3 loss in embryos or that specific FOXJ3 interactors at work in ESCs are lacking in embryos.

The transcriptional regulation of TEs and in particular that of MaLRs remains poorly understood. This is despite the fact that MaLRs constitute the largest proportion of the mouse embryonic transcriptome (Peaston et al, 2004; Franke et al, 2017; Oomen et al, 2025) and are also highly expressed in mammalian oocytes, specifically those from the MT family (Peaston et al, 2004; Franke et al, 2017; Yu et al, 2020). Their regulatory potential is also much less explored compared to e.g. MERVL and MERVL LTRs, but members of the MT MaLRs have been shown to be co-opted as oocyte-specific promoters in mice (Peaston et al, 2004; Franke et al, 2017). In addition, we recently showed that MaLRs function as alternative promoters in embryos from mouse and several other mammalian species (Oomen et al, 2025), leading to the formation of chimeric TE and host transcripts. Our data indicates that the expression of these chimeric TE-host transcripts is at least in part dependent on TBP.

Our data indicates that TBP binds to and regulates transcription of specific MaLRs, those from the ORR1A families, at the 2-cell stage. In oocytes, depletion of the oocyte-specific TBP paralogue, TBP2, leads to down-regulation of some members of the MT family of MaLR, such as MTA_Mm, MTB_Mm or MT-int, which were found to contain a high quality TATA-box motif (Yu et al, 2020). However, we did not identify such a motif within MaLR, suggesting that TBP regulation of ORR1A may occur without a strong TATA-box consensus. In fact, while TBP-sensitive genes show slightly higher ratio of TATA-box motif compared to all genes (*n* = 54,838; 86.4%), this ratio is similar in down (96.4%) and up-regulated genes (96.9%). It is interesting to note that TEs can be specifically regulated by such a general transcription factor, and that the two types of MaLRs with very distinct expression patterns appear specifically regulated by cell-type-specific TBP paralogues. Additional factors such as chromatin context and phylogenetic properties of specific insertions may also contribute to specific TE regulation (Franke et al, 2017; Carter et al, 2022; Oomen et al, 2025; Hermant et al, 2025).

While our CUT&Tag and gene expression data upon TBP loss suggest direct regulation of target TEs and a subset of ZGA genes, we cannot exclude indirect effects. General transcription factors (GTFs) from the basal machinery are known to make the first contact with the DNA for the pre-initiation complex (PIC) assembly, which includes binding of RNA Pol II and additional TFII factors (Reinberg et al, 1987; Buratowski et al, 1989, 1991). In particular, TBP alone can bend the DNA to direct RNA Polymerase II binding (Kim et al, 1993a, 1993b). However, additional TFs upstream of TBP may participate in this regulation, whereby a combinatorial action of several TFs is at play (Lee and Young, 1998; Li et al, 1999; Zhong et al, 2007). Regardless, whether TBP works together with additional factors, our data clearly shows that removing TBP induces downregulation of genes and TEs that are bound by TBP.

We also find that a minor fraction of specific MT2_Mm insertions is selectively regulated by TBP at ZGA. This could suggest that most insertions may have lost a dependency on TBP for their transcription or that they acquired additional TFBS, such as SRF, OBOX and DUX (Ji et al, 2023; Guo et al, 2024; Hermant et al, 2025) to maintain robust transcription at this stage. In line with these interpretations, our findings indicate that TBP binds to and regulates MaLR ORR1A1 and ORR1A0 during preimplantation development which are older than MT2_Mm (Franke et al, 2017). Overall, our data suggest that TBP binds and regulates the expression of TEs that are evolutionary older than MT2_Mm in mouse early embryos.

Despite the large transcriptional phenotype, TBP loss did not compromise development to blastocyst, but only led to a developmental delay. This contrasts with e.g. SPT5, which is also a global transcriptional regulator (Aoi et al, 2021; Hu et al, 2021), and its depletion in embryos using the same Trim-Away approach resulted in a fully penetrant 2-cell arrest (Abe et al, 2022). While we cannot exclude incomplete depletion of TBP in our experiments, and/or that the depletion is transient, these observations suggest that such a large transcription defect may not by itself compromise development per se and that instead specific genes may be more important. Indeed, while many ZGA genes are affected upon TBP loss, the classic “2C” markers genes such as the *Zscan4* family, *Zfp352*, *Pramef6* were not affected. Thus, TBP largely regulates a distinct part of ZGA not involving MT2_Mm. However, whether the developmental delay emerges due to downregulated TEs, genes, or a combination of both is unclear. We speculate that the combination of the two may contribute to the developmental delay. In addition, whether changes in gene expression upon TBP loss persist beyond the 2-cell stage remains to be determined.

Overall, our work highlights transcription factors governing the TE repertoire in early mouse embryos, shedding light into the molecular regulation of ZGA and TE usage at these early stages.

## Methods

**Table:** Reagents and tools table

Reagent/Resource	Reference or Source	Identifier or Catalog Number
**Experimental models**		
Mouse E14 ESC (2C:tbGFP-PEST)	Nakatani et al, 2022	
CD1 mouse preimplantation embryos		
C57BL/6 J × CBA/H (F1) mouse preimplantation embryos		
**Recombinant DNA**		
pCMV-Zscan4c	(Zhang et al, 2019a)	
pCMV6-Tbp	This study	
pCMV-MYC-Irf9	(Platanitis et al, 2019)	
pCMV-MYC-Srf	This study	
pCMV-MYC-Dux	This study	
pCMV-MYC-Zbtb7b	This study	
pCMV-MYC-Nobox	This study	
pCMV-MYC-Ehf	This study	
pCMV-MYC-Gabpa	This study	
pCMV-MYC-Foxj3	This study	
pCMV-MYC-Elf3	This study	
pCMV-MYC-Rfx7	This study	
pCMV-MYC-Smad3	This study	
pCMV-MYC-Meis2	This study	
pCMV5-Sox8	(Schmidt et al, 2003)	
pCMV-SPORT6-Zfp410	Dharmacon	
pIRES-NR2F2	This study	
pcDNA3-mA-MYB	(Trauth et al, 1994)	
pCAGIP-FLAG-MAFK	RIKEN	
pCIG-LMX1A	Addgene	
pCMV-SPORT6-HMBOX1	Dharmacon	Cat#: MMM1013-202769453
pCMV-GATA3	Addgene	Cat#: 83818
pIRES-DUXBL1	(Tagliaferri et al, 2019)	
pCAG-CRX-IRES-GFPd2	Addgene	Cat#: 73997
pcDNA3-ARID5A	(Amano et al, 2011)	
pCMV6-ZFP740	OriGene	Cat#: MR200524
pCMV6-ZFP281	OriGene	Cat#: MR215383
pCMV6-TCF7L2	OriGene	Cat#: MR224182
pCMV6-SOX15	OriGene	Cat#: MR225149
pCMV6-PKNOX1	OriGene	Cat#: MR222826
pCMV6-OBOX1	OriGene	Cat#: MR223867
pCMV6-OBOX2	OriGene	Cat#: MR219600
pCMV6-OBOX3	OriGene	Cat#: MR224473
pCMV6-OBOX5	OriGene	Cat#: MR202205
pCMV6-OBOX6	OriGene	Cat#: MR215428
pCMV6-LHX8	OriGene	Cat#: MR226908
pCMV6-KLF7	OriGene	Cat#: MR204201
pCMV6-HPB1	OriGene	Cat#: MR208277
pCMV6-FOXK1	OriGene	Cat#: MR222304
pCMV6-BBX	OriGene	Cat#: MR224547
pCMV6-ATF1	OriGene	Cat#: MR223254
pCMV-EMPTY	This study	
pGEMHE-mCherry-mTrim21	Addgene	Cat#: 105522
**Antibodies**		
CRX	Santa Cruz	sc-377138
GATA3	Cell Signaling	5852
SMAD3	Cell Signaling	9523
ZFP410	Proteintech	14529-1-AP
RFX7	Novus Biologicals	NBP1-71819
LMX1A	Invitrogen	PA5-115517
FOXJ3	Affinity Biosciences	af0602
TBP	(Gazdag et al, 2007)	N/A
TBP	From Laszlo Tora	N/A
Normal Mouse IgG	Merck Millipore	12-371
Normal Rabbit IgG	Cell Signaling	2729S
TBP	Abcam	Ab28175
Rabbit IgG Isotype Control	Invitrogen	10500C
Rabbit	ThermoFisher	A16110
Mouse	ThermoFisher	A16078
Rabbit	Invitrogen	A21429
Rabbit	Invitrogen	A21245
Mouse	Invitrogen	A11017
Rabbit	Antibodies-Online	ABIN101961
**Oligonucleotides and other sequence-based reagents**		
qPCR primers	This study or study referred to in Table EV3	Table EV3
**Chemicals, Enzymes and other reagents**		
AccuPrime Pfx DNA polymerase	Invitrogen	10472482
DNA ligation mix “Mighty Mix”	Takara	6023
GoScript Reverse Transcription System	Promega	A5000
GoTaq qPCR Master Mix	Promega	A6002
HiFi HotStart ReadyMix	KAPA	KM2605
High Sensitivity DNA Kit	Agilent	5067-4626
mMESSAGE mMACHINE T7 ULTRA Transcription Kit	Ambion	AM1345
NEBNext High-Fidelity 2X PCR Master Mix	NEB	M0541
Nextera XT DNA library Preparation Kit	Illumina	15032354
NucleoBond Xtra MidiKit	MACHEREY-NAGEL	740410.50
NucleoSpin Mini Kit	MACHEREY-NAGEL	740490.250
pA-Tn5 Adaptor Complex	Diagenode	C01090001-30
RNase-free DNase Set	Qiagen	79254
RNeasy MinElute Cleanup Kit	Qiagen	74204
RNeasy Mini Kit	Qiagen	74104
SuperScript II reverse transcriptase	ThermoFisher	18064014
Taq DNA polymerase	Thermo Scientific	EP0401
Trypsin-EDTA (0.25%), phenol red	Gibco	25200056
TURBO DNA-free Kit	Invitrogen	AM1907
10X lysis buffer	Clontech	635013
2-mercaptoethanol	Gibco	31350010
Agar	BD DIFCO	214530
Ammonium chloride	Carl Roth	P726.1
Ampicillin	Fisher BioReagents	10193433
AMPure XP beads	Beckman Coulter	A63881
Betaine	Sigma-Aldrich	B03001VL
BSA	Roche	10735078001
Cascade blue dextran	Invitrogen	D1976
CHIR99021	Cayman	13122-25
COmplete, EDTA-free Protease Inhibitor Cocktail (PIC)	Sigma-Aldrich	11873580001
DMEM with GlutaMAX	Gibco	31966047
dNTP mix	Thermo Scientific	R0192
DPBS	Gibco	14190144
EDTA	Carl Roth	8043.2
FCS	PAN-Biotech	P30-3302
Gelatin Solution (0.1% in PBS)	PAN-Biotech	P06-20410
GeneRuler 1 kb plus DNA ladder	Thermo Scientific	SM1331
Glycerol	Sigma-Aldrich	G5516-100ML
hCG	MSD Animal Health	
HEPES Buffer Solution (1 M)	Gibco	15630-056
Hyaluronidase	Sigma-Aldrich	H4272-30MG
Kanamycin	Thermo Fisher	BP906-S
KCl	Sigma-Aldrich	P5405
LIF	IGBMC	n/a
Lipofectamine 2000 Transfection Reagent	Invitrogen	11668-019
M2 medium	Sigma-Aldrich	M7167-100ML
MEM Amino Acids Solution	Gibco	11130077
MEM NEAA Solution	Gibco	11140-035
MgCl_2_	Sigma-Aldrich	M1028
NaCl (5 M), RNase-free	Invitrogen	10609823
Opti-MEM	Gibco	31985062
PageRuler Prestained Protein ladder	Thermo Scientific	26616
Paraffin Oil	Sigma-Aldrich	18512-1L
PD0325901	Miltenyi	130-106-541
PEG-8000	Sigma-Aldrich	P1458
Penicillin–streptomycin	Gibco	15070063
PFA	Sigma-Aldrich	158127
PFA (solution 16%)	Thermo Scientific Chemicals	043368.9M
PMSG	Ceva	
Poly-L-Lysine	Sigma-Aldrich	P4707-100ML
RNA Clean XP	Beckman Coulter	A66514
RNAse inhibitor	Takara	2313A
Spermidine	Sigma-Aldrich	S2501-1G
Sucrose	Sigma-Aldrich	S9378-500G
TAPS	Sigma-Aldrich	T5130
Tris Base	Millipore	1083820100
Triton X-100	Sigma-Aldrich	X100-100ML
Triton X-100 solution (~10% in H_2_O)	Sigma-Aldrich	93443-100 ML
Tween-20	Sigma-Aldrich	P6585
Tyrode’s solution, acidic	Sigma-Aldrich	T1788
UltraPure SDS Solution, 10%	Invitrogen	15553027
Vectashield with DAPI	Vector Laboratories	H-1200-10
**Software**		
Bowtie v2.2.9	Langmead and Salzberg, 2012	https://bowtie-bio.sourceforge.net/bowtie2/index.shtml
Samtools	Li et al, 2009	https://www.htslib.org/doc/samtools.html
NucleoATAC package v0.2.1	Schep et al, 2015	https://github.com/GreenleafLab/NucleoATAC
Picard	Broad Institute	https://broadinstitute.github.io/picard/
SeqMonk v.1.42.1	RRID:SCR_001913	https://www.bioinformatics.babraham.ac.uk/projects/seqmonk/
MEME suite v5.1.1	Bailey et al, 2015	https://meme-suite.org/meme/
R	R Core Team	https://www.r-project.org
STAR (v2.7.11a)	Dobin et al, 2013	https://anaconda.org/bioconda/star
Trimmomatic	Bolger et al, 2014	https://anaconda.org/bioconda/trimmomatic
TEtranscript	Jin et al, 2015	https://hammelllab.labsites.cshl.edu/software/
DESeq2	Love et al, 2014	https://bioconductor.org/packages/release/bioc/html/DESeq2.html
BEDtools	Quinlan and Hall	https://bedtools.readthedocs.io/en/latest/
Deeptools	Ramírez et al, 2016	https://anaconda.org/bioconda/deeptools
ChimeraTE	Oliveira et al, 2023	https://github.com/OliveiraDS-hub/ChimeraTE
**Other**		
Smart-seq+5 libraries processing tools	Oomen et al, 2025	https://github.com/meoomen/Smartseq5
Mouse reference genome GRCm38	ENSEMBL	http://ftp.ensembl.org/pub/release-101/fasta/mus_musculus/dna/Mus_musculus.GRCm38.dna.primary_assembly.fa.gz
ERCC spike-in sequences and annotations	Thermo Fisher	https://assets.thermofisher.com/TFS-Assets/LSG/manuals/ERCC92.zip
Gene annotations (gencode)	ENSEMBL	http://ftp.ebi.ac.uk/pub/databases/gencode/Gencode_mouse/release_M20/gencode.vM20.primary_assembly.annotation.gtf.gz
DBTMEE gene classification	Park et al, 2015	https://dbtmee.hgc.jp/download/data/tables.tar.gz
Illumina NovaSeq 6000 platform	Illumina	
Bioanalyzer with the High Sensitivity DNA kit	Agilent	
5200 Fragment Analyser System	Agilent	

### Footprinting analysis

ATAC sequencing reads were obtained from GEO accession GSE66390 (Wu et al, 2016). Reads were aligned to mm10 with bowtie2 v2.2.9 with the parameter -X 2000. Mitochondrial reads were removed with Samtools v1.9 and duplicate reads removed with Picard MarkDuplicates. Tn5 insertion sites were obtained genome wide using the pyatac ins function in the NucleoATAC package v0.2.1. Tn5 insertion sites were quantified and normalized for library size using SeqMonk v.1.42.1. Meta-repeat analyses were performed in Seqmonk by quantifying Tn5 insertion sites that mapped within intact regulatory elements of each repeat (LTR for MT2_Mm and ORR1A1/0; complete elements for SINEs; monomers for L1) annotated with RepeatMasker. Regulatory elements were considered intact if they corresponded to the length of the consensus sequence in Repbase. In the case of L1MdTf_II, only individual monomers of the 5’UTR were considered. The monomers coordinates were obtained by mapping the monomer consensus sequence using bowtie2 v2.2.9 with parameters -a -x, which annotation was then used to map Tn5 insertion sites as described above. Transcription factor footprints were identified qualitatively, by identification of a local depletion of signal, relative to a higher signal in the flanking regions. The sequence underlying each footprint was extracted from the Repbase consensus sequences, and subject to motif analysis using the Tomtom tool from the MEME suite v5.1.1 against the UniPROBE Mouse database using Euclidean distance and a significance threshold of E < 30. Using publicly available RNA-seq data (Deng et al, 2014), candidate transcription factors obtained from the footprints were filtered based on expression: two or more reads in all cells of the mid 2-cell and late 2-cell stages. The expression matrix from GEO accession GSE45719 (Deng et al, 2014) was downloaded from a GitHub repository (“jhsiao999/singleCellRNASeqMouseDengESC”). The expression matrix was normalized by library size by dividing the counts by the sum of expression across detected genes in each sample. The mean normalized relative RNA levels for TFs were plotted using ggplot2 with hierarchical clustering applied to rows. Expression analysis of TFs in ESCs was done from ArrayExpress accession E-MTAB-2684 (Ishiuchi et al, 2015). Reads were aligned to mm10 with STAR using parameter –quantMode GeneCounts to count reads. The expression matrix was normalized by number of exonic kb and library size (RPKM). The normalized RNA levels obtained for each TFs were plotted using ggplot2.

### Plasmids

The pCMV-*Zscan4c* plasmid was obtained from Liu Lin (Zhang et al, 2019). The pMRx-*ThPOK*-IREs-GFP was received from Rémy Bosselut and the *ThPOK* (*Zbtb7b*) CDS was cloned to pCMV vector (Wildt et al, 2007). *Tbp* CDS was cloned from pRN3p (Gazdag et al, 2009) to pCMV6. *Dux*, *Srf* and *Gabpa* cloning of CDS to pCMV were previously described (Hermant et al, 2025). pCMV5-*Sox8* was gifted from Michael Wegner (Schmidt et al, 2003). pCMV-SPORT6-*Zfp410* was purchased from Dharmacon (CAT#: MMM1013-202769453) and cloned to pCMV6-*Zfp410*. pZhC-*Nr2f2*-his-flag was received from Minoru Ko (Nishiyama et al, 2009) and cloned to pIRES. pCR4-TOPO-*Nobox* was purchased from Dharmacon (CAT#: MMM1013-211691656) and cloned to pCMV. pcDNA3-*mA-MYB* (*Mybl1*) was obtained from Karl-Heinz Klaupner (Trauth et al, 1994). pCAGIP-FLAG-*Mafk* was ordered from RIKEN (RDB15412). pCIG-*Lmx1a* was purchased from Addgene (Kathleen Millen; # 45070). pCMV-*Irf9* was obtained from Thomas Decker (Platanitis et al, 2019). pCMV-SPORT6-*Hmbox1* was purchased from Dharmacon (CAT#: MMM1013-202702395). pCMV-*Gata3* was purchased from Addgene (Douglas Engel, # 83818). *Foxj3, Elf3* and *Rfx7* CDS were amplified from mESCs cDNA and cloned to pCMV vector. The *Smad3* and *Meis2* CDS were amplified from MEFs cDNA and cloned to pCMV vector. pMXs-IRES-GFP containing flag tagged Mouse *Ehf* cDNA was obtained from Nobuhiro Nakano (Yamazaki et al, 2015) and cloned to pCMV. pIRES-*Duxbl1* was obtained from Geppino Falco (Tagliaferri et al, 2019). CAG-*Crx*-IRES-GFPd2 was purchased from Addgene (Connie Cepko; # 73997) (Wang et al, 2014). pcDNA3-*Arid5a* was received from Riko Nishimura (Amano et al, 2011). The pCMV6-*Zfp740* (CAT#: MR200524), pCMV6-*Zfp281* (CAT#: MR215383), pCMV6-*Tcf7l2* (CAT#: MR224182), pCMV6-*Sox15* (CAT#: MR225149), pCMV6-*Pknox1* (CAT#: MR222826), pCMV6-*Obox1* (CAT#: MR223867), pCMV6-*Obox2* (CAT#: MR219600), pCMV6-*Obox3* (CAT#: MR224473), pCMV6-*Obox5* (CAT#: MR202205), pCMV6-*Obox6* (CAT#: MR215428), pCMV6-*Lhx8* (CAT#: MR226908), pCMV6-*Klf7* (CAT#: MR204201), pCMV6-*Hbp1* (CAT#: MR208277), pCMV6-*Foxk1* (CAT#: MR222304), pCMV6-*Bbx* (CAT#: MR224547), pCMV6-*Atf1* (CAT#: MR223254) were all purchased from OriGene.

### ESC culture

Mouse E14 ESC lines carrying the 2C:tbGFP-PEST (Nakatani et al, 2022) were cultured in Dulbecco’s modified Eagle’s medium (DMEM) with GlutaMAX (Gibco, 31966047) containing 15% fetal calf serum, NEAA (ThermoScientific, 11140-035), 2X leukemia inhibitory factor (LIF), penicillin–streptomycin (Gibco, 15070063), 0.1 mM 2-mercaptoethanol (ThermoScientific, 31350010), 3 μM CHIR99021 (Cayman, 13122-25) and 1 μM PD0325901 (Miltenyi, 130-106-541) on gelatin-coated plates at 37 °C in 5% CO_2_. Micoplasm tests are performed yearly.

### Transfections

Transfections were performed using Lipofectamine 2000 (Invitrogen, 11668-019). 250,000 cells were transfected per condition in 6-well gelatin-coated plates. 2.5 μg of plasmid encoding for the CDS of each of the 40 TFs were transfected in duplicates (except for IRF9, CRX, HMBOX1, SOX15 and LHX8 which were performed in triplicates), alongside an empty CMV plasmid and no plasmid control. Forty-eight hours after transfection, cells were harvested and ½ of the cell pellets were kept at −80 °C, the other half was resuspended in SDS lysis buffer (2% SDS, 50 mM Tris-HCl ph 7.5, 10% glycerol) and kept at −20 °C.

### RNA extraction, RT and real-time RT-qPCR

RNA was extracted using RNeasy Kit (Qiagen, 74104) following manufacturer’s instructions. RNAs were treated with DNase in two steps with two different enzymes, first the RNase-free DNase I (Qiagen, 79254) for 30’ on RNA extraction column. 5 μg of RNA was used for 1H TURBO DNase treatment with the TURBO DNA-free kit (Invitrogen, AM1907) following manufacturer’s instructions. The RNA was subsequently purified again using the RNeasy MinElute Cleanup Kit (Qiagen, 74204). For each sample, 1 μg of RNA was used to generate cDNA using the GoScript Reverse Transcription System (Promega, A5000) with random hexamers. For each RT reaction, identical reaction was conducted without RT enzyme (no RT control). Real-time PCR was done using the GoTaq qPCR Master Mix (Promega, A6002) and a Roche Applied Science Lightcycler (LightCycler96). Cycling conditions were as follows: 600 s at 95 °C, 10 s at 95 °C, 10 s at 55 °C and 10 s at 72 °C, for a total of 45 cycles. The sequences for the primers used are provided in Table EV2. Primer sequences for TEs were either obtained from previous publications or PCR products were verified by Sanger sequencing. PCR were performed in triplicates, melting curve analysis was performed for each sample to control for proper amplification, and the mean Ct was calculated. In addition, no RT control PCR were conducted for each sample in triplicate and analysed to control for DNA contamination. Ct values were normalized using Actin primer set, and fold change over no vector control was calculated. Empty CMV control vector fold change over no vector control was also calculated and always analyzed aside every sample. The mean fold change over the 2 (or three) biological replicates for all TFs were plotted using ggplot2 with hierarchical clustering applied to rows. Since these experiments were performed in biological duplicates, no statistics were applied to these results.

### Embryo collection

All animal experiments were in compliance of the legislation from the Government of Upper Bavaria. For immunostainings, CD1 females (6–10 weeks old) were mated with CD1 males (2–8 months old). Zygotes, early 2-cell, late 2-cell, 4-cell and 8-cell were collected at ~16 h, ~32–33 h, ~41–42 h, ~48 h, ~56–57 h post coitum, respectively. For microinjections and CUT&Tag, F1 (C57BL/6J × CBA/H) females (<10 weeks old) were mated with F1 males (3–6 months old). Ovulation was induced by injection of pregnant mare serum gonadrotropin (PMSG, Ceva) followed by human chorionic gonadotropin (hCG, MSD Animal Health) 48 h later. Mice were maintained under a 12 h light cycle at 20–24 °C and 45–65% humidity and constant access to food and water.

### Immunostainings

Immunostainings were performed as described previously (Torres-Padilla et al, 2006). The zona pellucida was removed with acid Tyrode (Sigma-Aldrich). After PBS wash, the embryos were fixed in 4% PFA, 0.04% Triton, 0.3% Tween-20, 0.2% sucrose on a glass-bottom dish at 37 °C for 20 min. Embryos were washed 3X in PBS and permeabilized with 0.5% Triton X-100 for 20 min at RT. After three washes in PBST (0.1% Tween 20 in PBS), embryos were incubated in 2.6 mg/ml ammonium chloride (in PBS) solution, washed again twice in PBST and blocked for 4–5 h in 3% BSA in PBST at 4 °C. Overnight incubation with primary antibody in 3% BSA was performed. The list of primary antibodies used as well as the dilutions are described in Table EV3. The day after, embryos were washed three times in PBST, briefly blocked (20 min in 3% BSA), and incubated with the secondary antibody for 3–4 h at RT in 3% BSA. Secondary antibodies and dilutions are described in Table EV3. Finally, embryos were washed twice in PBST, once in PBS for 20 min and mounted in vectashield containing DAPI (Vector Laboratories, H-2000). For anti-CRX antibody positive control in ESCs, cells were transfected as described above and seeded directly onto coverslips. 24 h later, cells were washed twice with PBS and fixed for 15 min in 3% PFA in PBS. Cells were subsequently washed three times with PBS and permeabilized 5 min in ice-cold 0.5% triton in PBS. Cells were then washed once and incubated for 15 min in blocking buffer (3% BSA, 0.1% triton in PBS). Primary antibody incubation was performed in washing buffer (1% BSA, 0.05% Triton) for 45 min with primary antibody (Table EV3). Cells were washed three times in washing buffer, and incubated for 30–40 min in washing buffer with the secondary antibody (Table EV3). Cells were finally washed three times in washing buffer and mounted in vectashield containing DAPI (Vector Laboratories, H-2000). Confocal microscopy was done using 63x oil objective of SP8 microscope (Leica).

### In vitro transcription and antibody purification

The mCherry-Trim21 plasmid was obtained from Addgene (# 105522) (Clift et al, 2017). mRNA was transcribed in vitro from the T7 promoter using the mMESSAGE mMACHINE T7 Ultra kit (Ambion #AM1345). 50 μg of each antibody used for Trim-Away were purified using Amicon Ultra 100 K Centrifugal Filter (Merck Millipore #UFC510024), following manufacturer’s instruction without addition of NP-40.

### Trim-away

Zygotes were collected between 17 and 18 h post-hCG from the oviducts of the females. Brief incubation with M2 containing hyaluronidase (Sigma-Aldrich) was performed to remove cumulus cells. Zygotes were microinjected with 0.33% cascade-blue-coupled dextran (Invitrogen, D1976) and 500 ng/μl of purified antibody in PBS. Antibodies used were the following: the mouse monoclonal 3G3 anti-TBP (Gazdag et al, 2007; Brou et al, 1993) and the normal mouse IgG (mIgG) (Merck Millipore, 12-371) for the TBP loss of function. For the FOXJ3 loss of function, the rabbit polyclonal anti-FOXJ3 (Affinity Biosciences, af0602) and the normal rabbit IgG (rIgG) (Cell Signaling, 2729S) were used. Zygotes were cultured for 4 h in K-modified simplex optimized medium (KSOM) microdrops under paraffin oil (Sigma) at 37 °C and 5% CO_2_. The zygotes were then injected a second time with 200 ng/μl Trim21-mCherry mRNA, and kept in culture until collection at 48 h post-hCG for single embryo RNA-seq. 10 TBP LOF with 9 CONTROL (mIgG), 17 FOXJ3 LOF and 13 CONTROL (rIgG) embryos from at least 3 independent experiments were collected for Smart-seq+5’ and 1 TBP LOF CONTROL embryo as well as 1 FOXJ3 LOF embryo were removed after quality control analysis. For every collection, a few embryos were kept for immunostainings to control for proper protein depletion, performed as described above using antibodies described in Table EV3. For TBP LOF development experiments, TBP LOF and CONTROL embryos were injected as indicated above, and development was monitored at 48 h, 66 h, 72 h, 96 post-hCG. Sample size was chosen in order to ensure that the data was consistent and reproducible based on previously published work and preliminary studies as standard for this field of research.

### SMART-seq + 5’

SMART-seq+5’ was modified from Smart-seq2 protocol (Picelli et al, 2013, 2014) as previously described (Oomen et al, 2025; Hermant et al, 2025). For TBP LOF all samples were collected in the same lysis buffer, which was stored at −80 °C until use (Clontech 10X lysis buffer (635015) diluted to 1X in H_2_O supplemented with ERCC (External RNA Controls Consortium) RNA spike-ins (diluted to 1:581,000) and aliquoted in PCR tubes (5.8 μl/tube)). For FOXJ3 LOF, a first batch of library preparation was performed with all samples collected in the same buffer aliquot (10 FOXJ3 LOF and 7 CONTROL embryos) and processed together. A second batch of library preparation was done from embryos collected using the same buffer, but from a different aliquot (7 FOXJ3 LOF and 6 CONTROL embryos) and thus processed as a different batch. At the time of collection (48 h post-hCG), embryos were washed 3X in PBS, transferred to tubes containing lysis buffer, snap-frozen in liquid nitrogen and kept at −80 °C until further processing. RNA was extracted (using RNA beads) and resuspended in 1 μL of dNTP mix (ThermoFisher, R0192), 1 µL of oligo-dT30V (10 μM, Sigma, 5’-AAGCAGTGGTATCAACGCAGAGTACT30V-3’) and 1 µl of nuclease-free H_2_O containing 5% RNAse inhibitor (Takara, 2313 A). The samples were first incubated for 3 min at 72 °C and kept on ice until further processing. In the meantime, the reverse transcription solution was prepared, for one sample: 2 µl of Superscript II 5x RT buffer (ThermoFisher, 18064014), 1.6 µl PEG-8000 40% (Sigma, P1458), 0.5 µl DTT, 0.25 RNAse inhibitors (Takara, 2313A), 0.1 µl 100 µM TSO (TIB MolBiol, 5’-AAGCAGTGGTATCAACGCAGAGTACATrGrG+G-3’), 0.06 µl MgCl_2_ 1 M (Sigma, M1028), 2 µl Betaine 5 M (Sigma, B0300-1VL) and 0.5 µl Superscript II RT (ThermoFisher, 18064014). 7 µl of the reverse transcription mix was added to the 3 µl of annealed RNA mix and incubated for 90 min at 42 °C followed by 15 min at 70 °C. Preamplification of the obtained cDNA was performed using KAPA HiFi ReadyMix (KM2605) for 14 cycles with ISPCR primers (10 µM, Sigma, 5′-AAGCAGTGGTATCAACGCAGAGT-3′), and purified using with Agencourt Ampure XP beads (Beckman Coulter, A63881). 2.5 µl of 120 pg/µl cDNA for each sample was used for tagmentation, which was performed with the Nextera XT kit (Illumina, 15032354). The preamplified cDNA was mixed with 5 µl of Tagment DNA buffer and 2.5 µl of Amplicon Tagment Mix and incubated at 55 °C for 5 min. The tagmentation reaction was stopped with 2.5 µl NT buffer, and samples were incubated at RT for 5 min. Tagmented DNA was then amplified for 12 cycles using the two standard i5 and i7 Nextera Unique Double Indexes together with a tailed i7 index containing an overhang enabling the capture of the 5’ of the transcripts. The libraries were verified using the 2100 Bioanalyzer with the High Sensitivity DNA kit (Agilent). 150 bp paired-end sequencing protocol was used on Illumina NovaSeq 6000 platform.

### SMART-seq + 5’ analysis

Smart-seq+5’ libraries were processed as previously described (Oomen et al, 2025; Hermant et al, 2025) as indicated in https://github.com/meoomen/Smartseq5. Sequence quality was verified using FASTQC, and adaptor and low-quality sequences were removed using Trimmomatic (Bolger et al, 2014) configured in paired-end (PE) mode. In brief, a custom Python script was used to sort between 5’ transcript ends and internal transcript fragments according to their adaptor sequence. All analyses were performed with the 5’ reads. The reference genome was modified to contain the construct “pGEMHE-mCherry-mTrim21” fasta file, and the endogenous Trim-21 locus was masked using bedtools (version 2.31.0). The modified reference fasta file was used to prepare STAR index files. The reads were mapped to GRCm38 using STAR (v2.7.11a). A custom Perl script and the samtools package (version 1.20) (Li et al, 2009) were used to modify BAM files and keep only the read2. TEcount and TElocal from TEtranscript (Jin et al, 2015) were used to count TE and gene reads. L1MdTf_II elements were removed from the RepeatMasker annotation file (mm10_rmsk.gtf) to avoid double counting of reads after addition of the L1MdTf_II monomer coordinates to the annotation file. The modified annotation used was the same as the one described in (Hermant et al, 2025). TEcount was also used to count reads coming from genes, using the gencode M20 gene annotation. Subfamily information in the annotation file from previous phylogenetic studies were not taken into consideration for this analysis. For TElocal, we created an index file based on our modified TE annotation with the TElocal_indexer script available from https://github.com/mhammell-laboratory/TElocal (Jin et al, 2015). Reads from individual insertions were counted using this index file. Expression analysis across development stages was performed directly using the BAM files containing the 5’ reads from (Oomen et al, 2025). TEcount was used to count gene and TE reads using the same annotation files as described above. Plots for both genes and TEs were made using ggplot2 (version 3.5.1) in R.

### Differential expression analysis

The sample count table generated by TEcount from the 5’ reads were merged into two tables (TBP LOF and CONTROL; FOXJ3 LOF and CONTROL) using a bash script and then loaded in R. Reads per million (rpm) were calculated for each sample. Quality control thresholds were used as follows: a minimum of 5 × 10^5^ reads, a minimum of 1000 detected genes, the maximum percentage of reads assigned to mitochondrial DNA was set to 10 percent, and the maximum percentage of reads assigned to ERCC spike-ins was set to 15 percent. From these criteria, two embryos were removed from further analysis. Only genes and TEs with at least one read in at least 25% of the total samples were considered expressed and included in the differential expression analysis. Differential expression analysis was performed independently for FOXJ3 and TBP LOF using DESeq2 (v1.38.3) (Love et al, 2014) with read counts per gene and TEs calculated by TEcount. For the FOXJ3 embryos, batch was added as co-variate to the DESeq2 model and batch effect correction was applied. In the case of TBP, as the effect of TBP removal on the transcriptome was extensive, ERCCs were used as size vector for normalization in DESeq2. MA plots were used to present the differential expression analysis, showing the log2 fold change between the LOF experiment and its respective control. Scattermore package (v1.2) was used for plotting gene MA plots. Adjusted *p* value (padj) threshold was set to 0.05 for significance. Genes were assigned to major or minor ZGA using the Database of Transcriptome in Mouse Early Embryo (DBTMEE) (Park et al, 2015). For comparison with RNA-seq datasets from embryos upon CDK9 inhibition or, SPT5, OBOX and DUX loss-of-function, publicly available datasets were used (Abe et al, 2022; Ji et al, 2023; Hermant et al, 2025). Data were visualized on scatterplots using the scattermore package (v1.2) or with Venn diagrams using the VennDiagram package in R (v1.7.3). For scatterplots comparing log2 fold change values, DESeq2 result tables were merged by gene identifiers, and only genes present in both tables were included. For embryonic PCA analysis, embryos from the different embryonic stage from Oomen et al, TBP LOF embryos and CONTROL embryos counts were log2-transformed to generate the PCA. For single insertion differential expression analysis, the sample count table from TElocal for TBP LOF and CONTROL embryos were merged into a single table using the same bash script as described above and loaded in R. Representation, filtering and significance were performed as described above.

### CUT&Tag

50–70 and 85–100 late 2-cell stage embryos, were collected from the females between 46–48 h post-hCG for TBP and IgG control replicates, respectively. The embryos were washed in M2, in PBS, in ice-cold NE1 buffer (HEPES-KOH (pH 7.9) 20 mM, KCl 10 mM, Spermidine 0.5 mM (Sigma, S2501-1g), Triton 0.1%, PIC 1x (Sigma, 11873580001)) and transferred to another ice-cold NE1 buffer drop for 10 min incubation on ice. Embryos were subsequently washed in PBS, and in 150-Wash buffer (HEPES-NaOH (pH 7.5) 20 mM, NaCl 150 mM, Spermidine 0.5 mM (Sigma, S2501-1g), 1X PIC (Sigma, 11873580001)), followed by overnight incubation in antibody buffer (0.002 M EDTA, 1% BSA in Wash buffer 150) with anti-TBP antibody (Abcam ab28175, 1/100 dilution) or rabbit IgG Isotype Control (Invitrogen 10500C, 1/100 dilution from 1 mg/ml). We validated this antibody and our low input CUT&Tag protocol on 500 mouse ESCs against TBP CUT&Tag profiles published by Kwan et al (2023), which revealed highly similar profiles. Embryos were transferred to a secondary antibody solution with guinea pig anti-rabbit antibody (Antibodies-Online ABIN101961, 1/100 dilution) in 150-Wash buffer for 30 min at RT. Embryos were further washed in 150-Wash buffer, and transferred to pA-Tn5 adaptor complex (Diagenode C01090001-30, 1/200 dilution) solution in 300-Wash buffer (HEPES-NaOH (pH 7.5) 20 mM, NaCl 300 mM, Spermidine 0.5 mM (Sigma, S2501-1g), 1X PIC (Sigma, 11873580001)) for 1H at RT. After washes in 300-Wash buffer, tagmentation was performed in freshly made tagmentation buffer (10 mM MgCl_2_ in 300-Wash buffer) for 1H in 37 °C incubator. Embryos were subsequently washed in freshly made TAPS buffer (10 mM TAPS (pH 8.5), 0.0002 M EDTA in H_2_O), and transferred to a PCR strip containing 5 µL SDS release buffer (10 mM TAPS (pH 8.5), 0.1% SDS in H_2_O). Release was performed for 1H at 58 °C, and quenched with addition of 15 µL of Triton-X quench buffer (0.67% Triton in H_2_O). 2 µL of 10 µM barcoded i5 and i7 primer solutions were added to each sample as well as 25 µL NEBNext HiFi 2x PCR master mix (NEB, M0541) and amplified for 18 cycles or 22 cycles, for TBP and IgG embryos, respectively. The libraries were purified using Agencourt Ampure XP beads (Beckman Coulter, A63881) and verified using 5200 Fragment Analyser System (Agilent). 150 bp paired-end sequencing protocol was used on Illumina NovaSeq 6000 platform.

### CUT&Tag analysis

#### Mapping

CUT&Tag sequence reads were trimmed for sequence adaptors and low quality ends using Trimmomatic (Bolger et al, 2014). Paired-end reads were then mapped to mm10 using bowtie2 (Langmead and Salzberg, 2012) maintaining a maximum insert length of 2000bp (-x 2000). Using samtools, only paired, unique mapping alignments with a minimum alignment length of at least 30 bp were kept for downstream analysis. Optical and PCR duplicates were removed using picard (http://broadinstitute.github.io/picard). Mitochondrial reads were removed. Libraries were normalized to rpm values and visualized as bigwigs using bedtools genomecov and bedGraphToBigWig. Replicates were merged on a bigwig level using mean values. CUT&Tag libraries were not selected for insert length due to low sequence depth/complexity, however, this was taken into account in our QC assessment of all libraries.

#### Signal visualization

IgG or TBP signal was visualized as a pile-up on genes or TEs using deeptools (Ramírez et al, 2016). For signal visualization on genes, the TSS annotation for mm10 (gencode M20 gene annotation) was used using deeptools “reference-point”. For TEs, the modified RepeatMasker annotation used for the RNA-seq analysis was used with deeptools “scale-regions” where the average TE length was used as region length. Both genes and TEs were categorized as significant up/down or non-significant based on the differential expression analysis detailed previously.

### Log fold change analysis of neighbouring genes of TBP-bound TEs

TBP-bound insertions for both ORR1A0 and ORR1A1 were categorized based on deeptools matrices sorted by CUT&Tag TBP signal, taking the top 25% rows only. Genes were identified based on TSS positioning within 10 kb of any insertion (“all inserts”) or within 10 kb of a top-25% TBP bound TE insertion (“TBP bound inserts”) using bedtools windows. Log2 fold change of these genes was plotted based on differential expression analysis as described above. Statistical significance was computed using a two-sided, unpaired t-test.

### Analysis of TE-initiated chimeric transcripts

Mapped quality-passed 5’ fragments read pairs were converted back to fastq format using samtools fastq. Converted fastq files were used as input for ChimeraTE (Oliveira et al, 2023) mode-1 with parameters –strand rf-stranded and --window 150000. Only TE-initiated chimeric transcript present in 2 or more replicates per experimental condition were used in downstream analysis and visualization. Taking only chimeric transcripts present in our control data, the combined relative log2 counts of all chimeric reads for ORR1A0 and ORR1A1 for each condition were plotted as boxplots using ggplot2 in R. Statistical significance was computed using a two-sided, unpaired t-test.

## Supplementary information

Table EV1

Table EV2

Table EV3

Peer Review File

Dataset EV1

Dataset EV2

Dataset EV3

Dataset EV4

Source data Fig. 2

Source data Fig. 3

Source data Fig. 4

Figure EV2 Source Data

Figure EV3 Source Data

Figure EV4 Source Data

Expanded View Figures

## Data Availability

The RNA-seq and CUT&Tag data from this study are available from the Gene Expression Omnibus (GEO) database, accession number GSE288110 (RNA-seq) and GSE288111 (CUT&Tag). All other data supporting the findings of this study are available from the corresponding author upon reasonable request. The source data of this paper are collected in the following database record: biostudies:S-SCDT-10_1038-S44318-026-00736-w.
