# Supplementary figures and images for: TBP regulates transposable element expression in early mouse embryos
Source: EMBO J. 2026 Mar 20;45(9):3073–101. doi: 10.1038/s44318-026-00736-w (PMC13144441; doi:10.1038/s44318-026-00736-w)

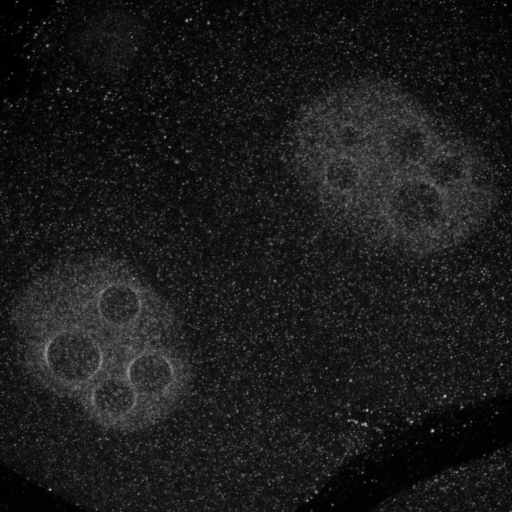

Supplement: Supplementary file 9 — Source data Fig. 2 [file 44318_2026_736_MOESM9_ESM.zip › Figure2/Panel_G/Early 2-cell.tif]

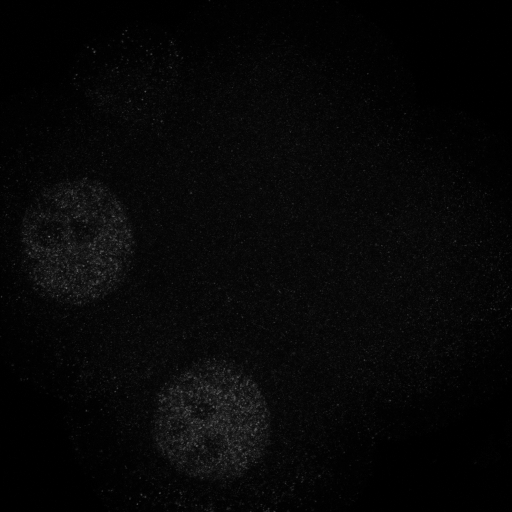

Supplement: Supplementary file 9 — Source data Fig. 2 [file 44318_2026_736_MOESM9_ESM.zip › Figure2/Panel_G/4-cell.tif]

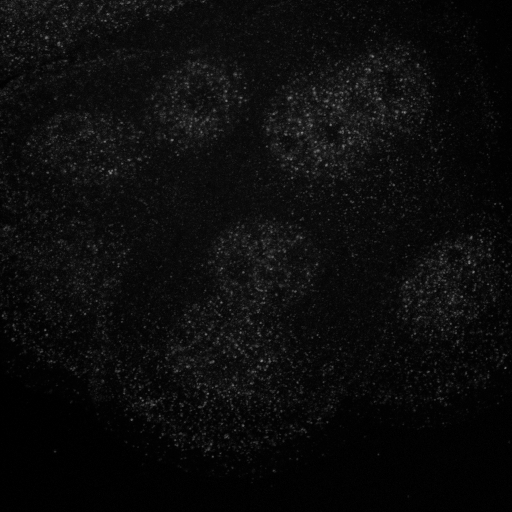

Supplement: Supplementary file 9 — Source data Fig. 2 [file 44318_2026_736_MOESM9_ESM.zip › Figure2/Panel_G/8-cell.tif]

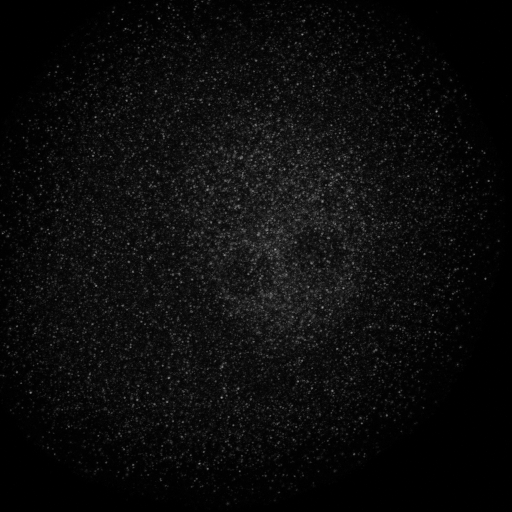

Supplement: Supplementary file 9 — Source data Fig. 2 [file 44318_2026_736_MOESM9_ESM.zip › Figure2/Panel_G/Zygote.tif]

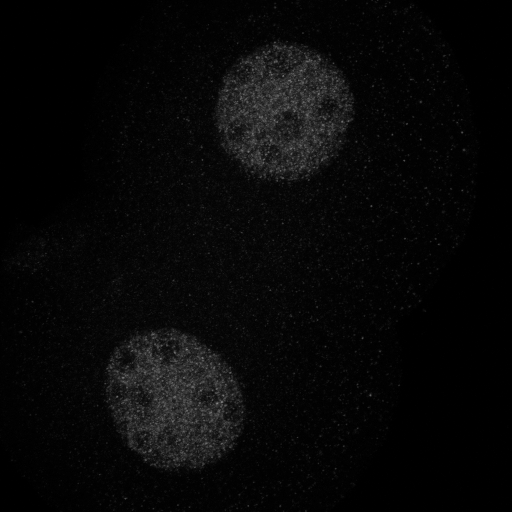

Supplement: Supplementary file 9 — Source data Fig. 2 [file 44318_2026_736_MOESM9_ESM.zip › Figure2/Panel_G/Late 2-cell.tif]

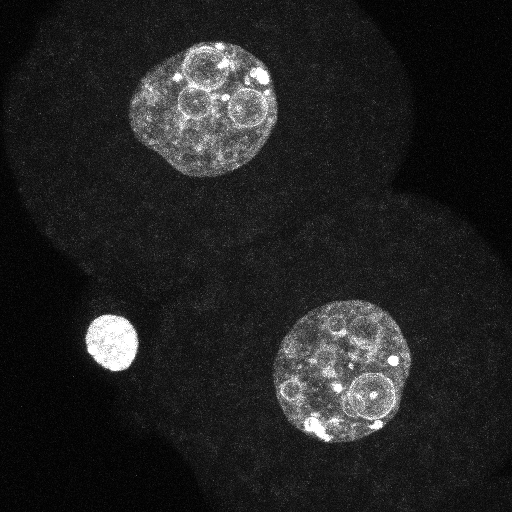

Supplement: Supplementary file 9 — Source data Fig. 2 [file 44318_2026_736_MOESM9_ESM.zip › Figure2/Panel_H/Early 2-cell_DAPI.tif]

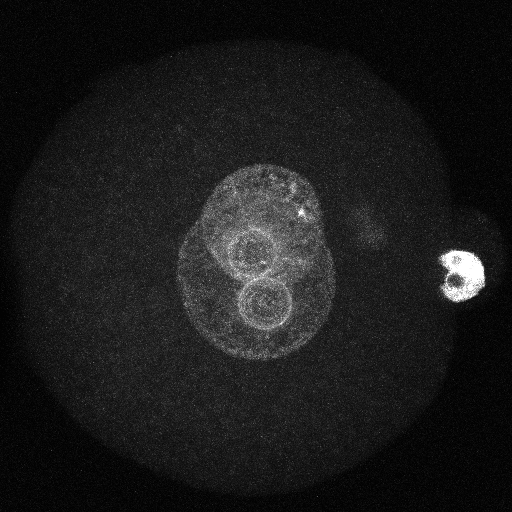

Supplement: Supplementary file 9 — Source data Fig. 2 [file 44318_2026_736_MOESM9_ESM.zip › Figure2/Panel_H/Zygote_DAPI.tif]

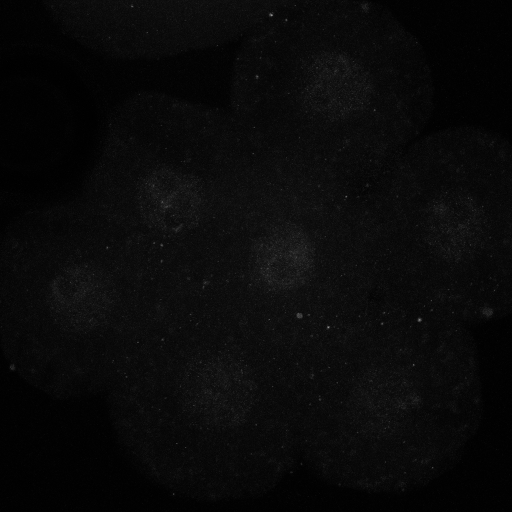

Supplement: Supplementary file 9 — Source data Fig. 2 [file 44318_2026_736_MOESM9_ESM.zip › Figure2/Panel_H/8-cell_TBP.tif]

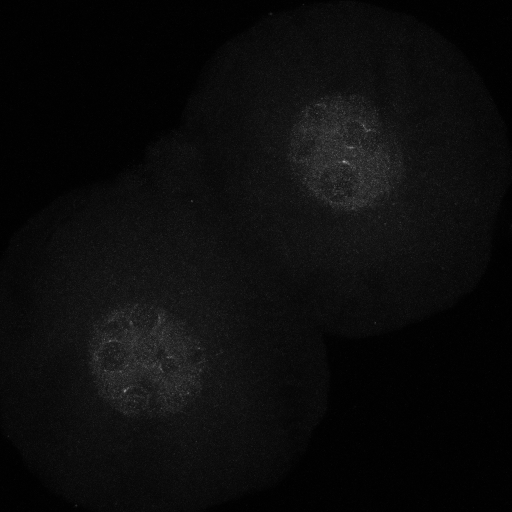

Supplement: Supplementary file 9 — Source data Fig. 2 [file 44318_2026_736_MOESM9_ESM.zip › Figure2/Panel_H/Late 2-cell_TBP.tif]

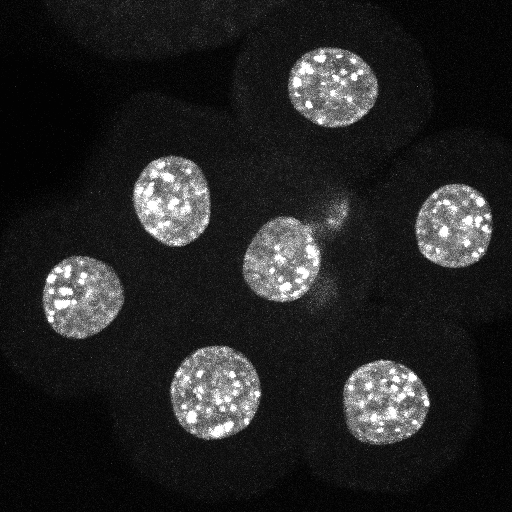

Supplement: Supplementary file 9 — Source data Fig. 2 [file 44318_2026_736_MOESM9_ESM.zip › Figure2/Panel_H/8-cell_DAPI.tif]

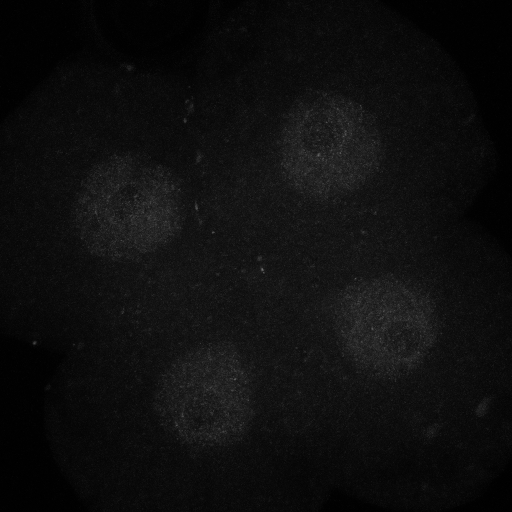

Supplement: Supplementary file 9 — Source data Fig. 2 [file 44318_2026_736_MOESM9_ESM.zip › Figure2/Panel_H/4-cell_TBP.tif]

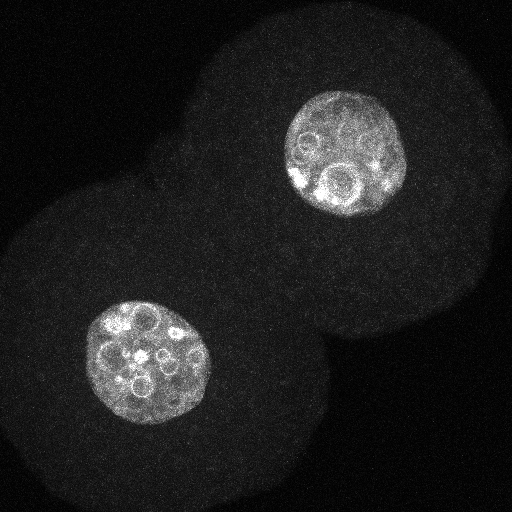

Supplement: Supplementary file 9 — Source data Fig. 2 [file 44318_2026_736_MOESM9_ESM.zip › Figure2/Panel_H/Late 2-cell_DAPI.tif]

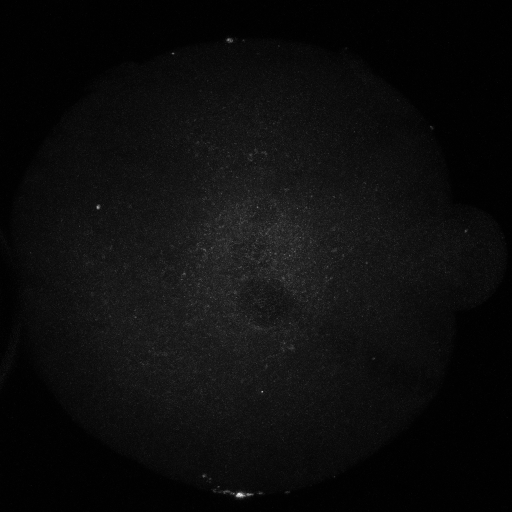

Supplement: Supplementary file 9 — Source data Fig. 2 [file 44318_2026_736_MOESM9_ESM.zip › Figure2/Panel_H/Zygote_TBP.tif]

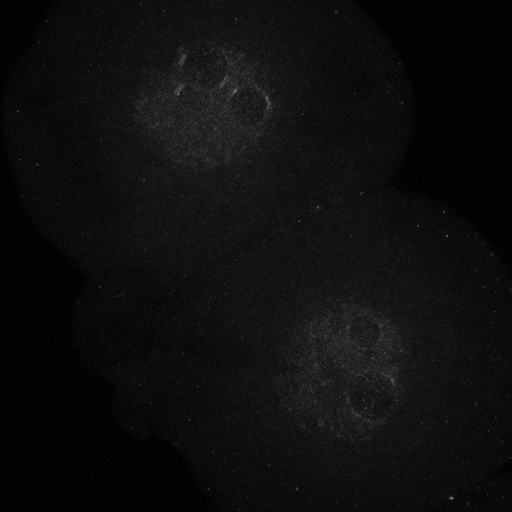

Supplement: Supplementary file 9 — Source data Fig. 2 [file 44318_2026_736_MOESM9_ESM.zip › Figure2/Panel_H/Early 2-cell_TBP.tif]

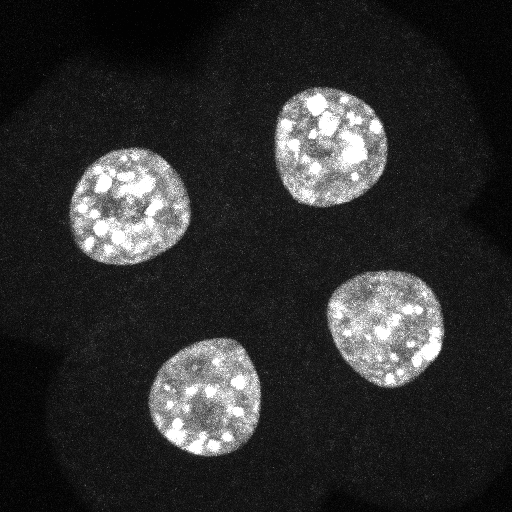

Supplement: Supplementary file 9 — Source data Fig. 2 [file 44318_2026_736_MOESM9_ESM.zip › Figure2/Panel_H/4-cell_DAPI.tif]

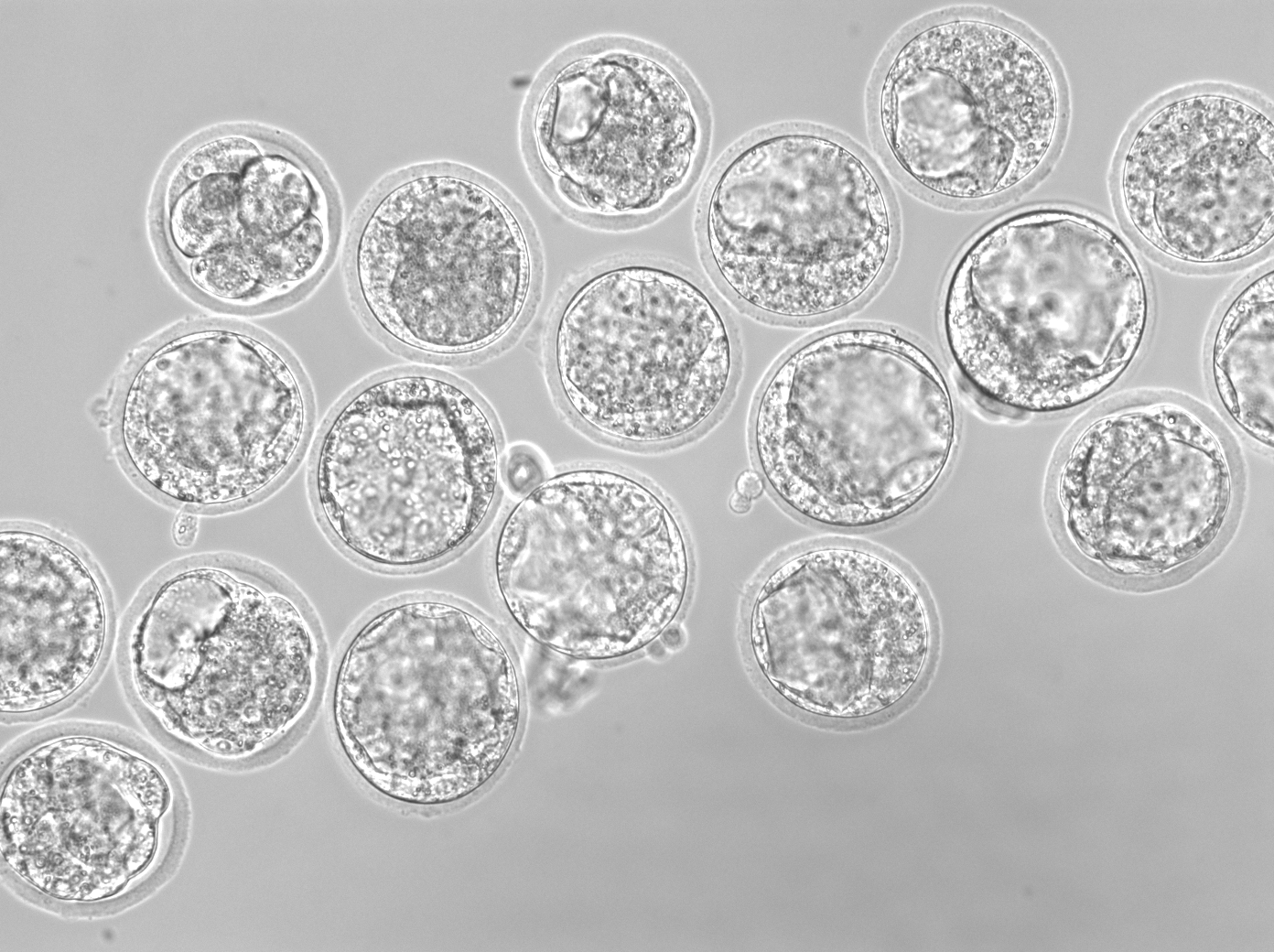

Supplement: Supplementary file 10 — Source data Fig. 3 [file 44318_2026_736_MOESM10_ESM.zip › Figure3/Panel H/images/CONTROL.tif]

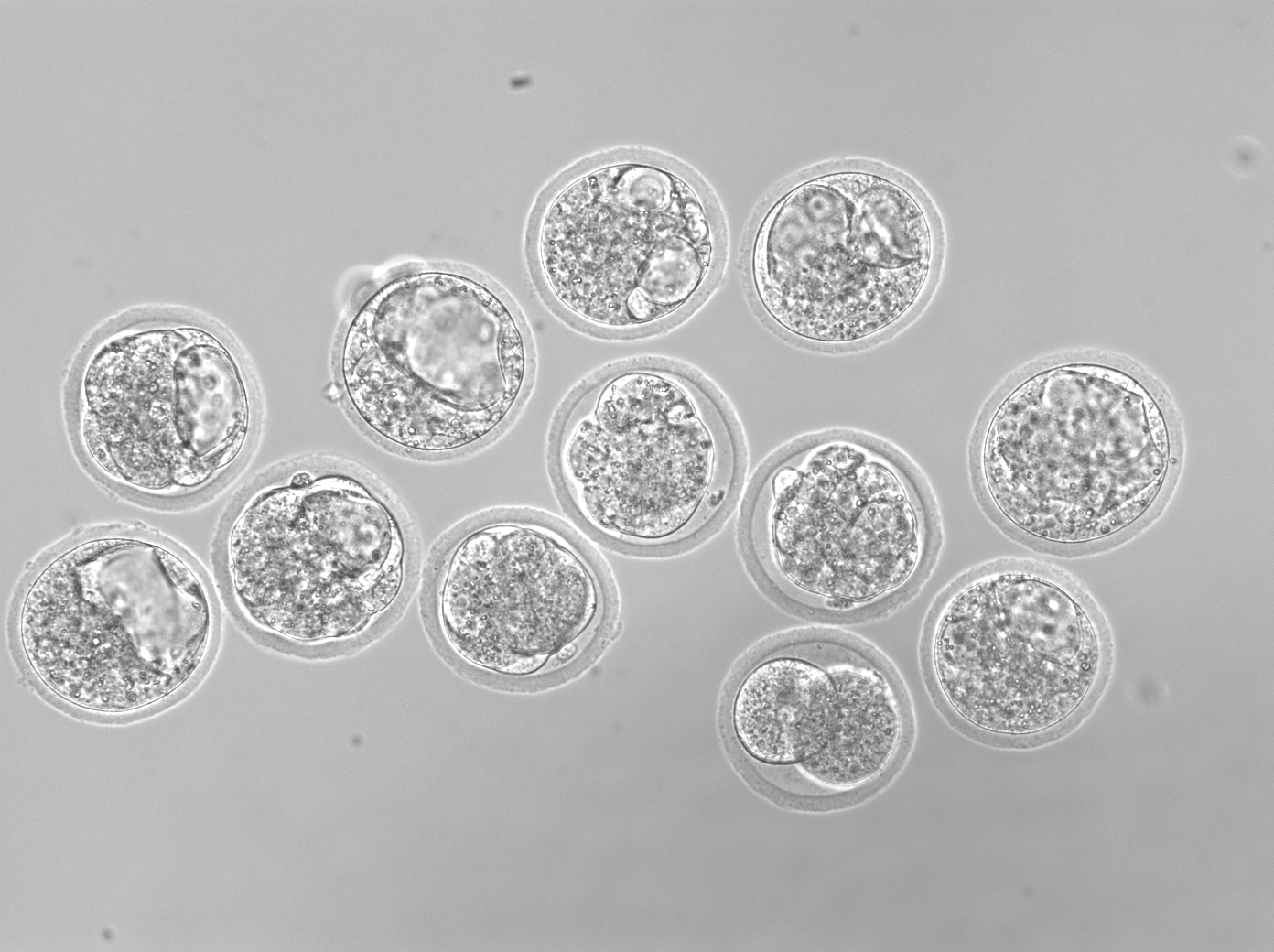

Supplement: Supplementary file 10 — Source data Fig. 3 [file 44318_2026_736_MOESM10_ESM.zip › Figure3/Panel H/images/TBP LOF.tif]

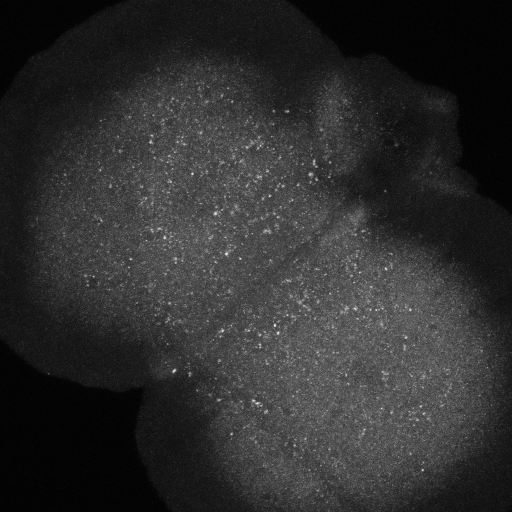

Supplement: Supplementary file 13 — Figure EV3 Source Data [file 44318_2026_736_MOESM13_ESM.zip › Figure EV3/panel L/Early 2-cell.tif]

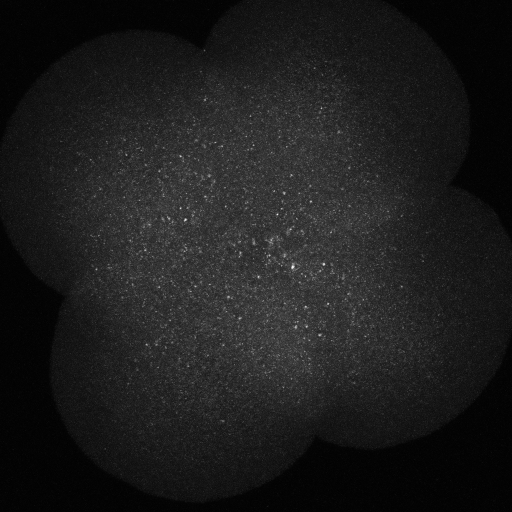

Supplement: Supplementary file 13 — Figure EV3 Source Data [file 44318_2026_736_MOESM13_ESM.zip › Figure EV3/panel L/4-cell.tif]

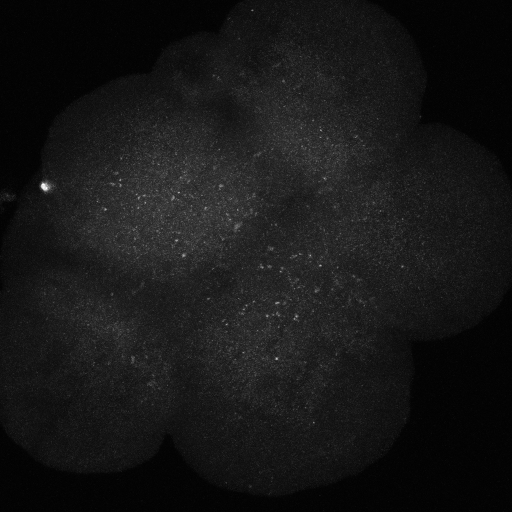

Supplement: Supplementary file 13 — Figure EV3 Source Data [file 44318_2026_736_MOESM13_ESM.zip › Figure EV3/panel L/8-cell.tif]

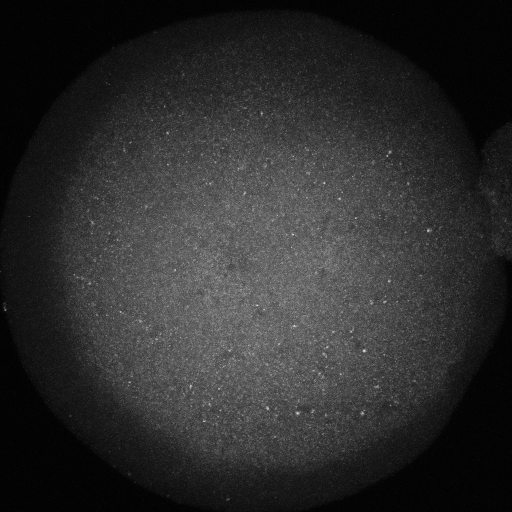

Supplement: Supplementary file 13 — Figure EV3 Source Data [file 44318_2026_736_MOESM13_ESM.zip › Figure EV3/panel L/Zygote.tif]

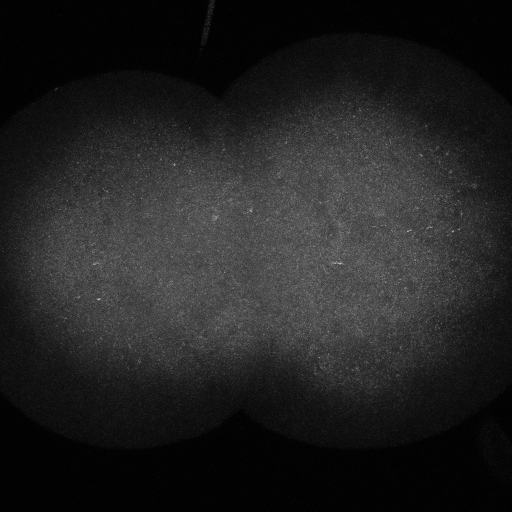

Supplement: Supplementary file 13 — Figure EV3 Source Data [file 44318_2026_736_MOESM13_ESM.zip › Figure EV3/panel L/Late 2-cell.tif]

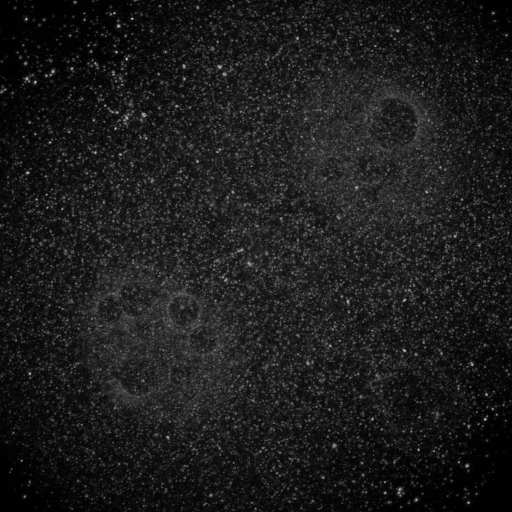

Supplement: Supplementary file 13 — Figure EV3 Source Data [file 44318_2026_736_MOESM13_ESM.zip › Figure EV3/panel K/Early 2-cell.tif]

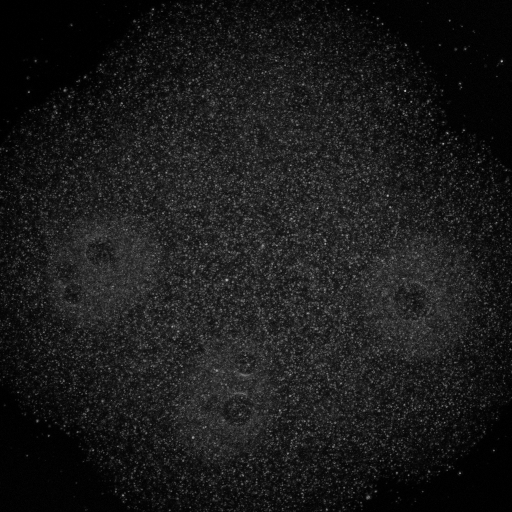

Supplement: Supplementary file 13 — Figure EV3 Source Data [file 44318_2026_736_MOESM13_ESM.zip › Figure EV3/panel K/4-cell.tif]

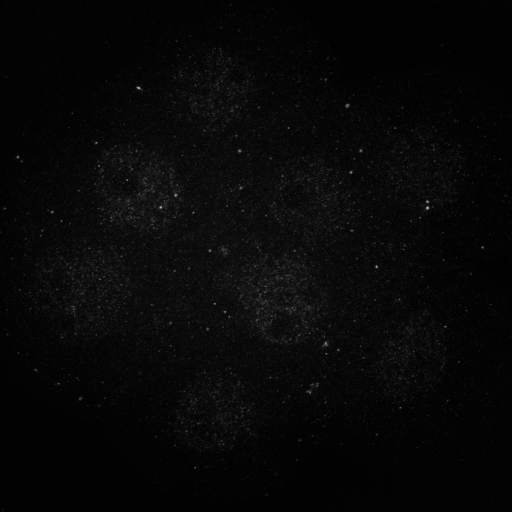

Supplement: Supplementary file 13 — Figure EV3 Source Data [file 44318_2026_736_MOESM13_ESM.zip › Figure EV3/panel K/8-cell.tif]

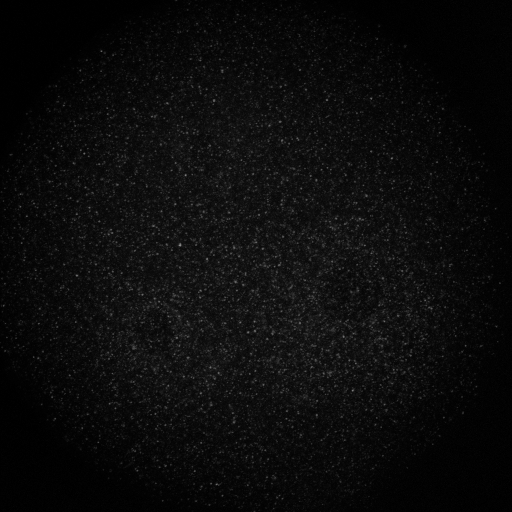

Supplement: Supplementary file 13 — Figure EV3 Source Data [file 44318_2026_736_MOESM13_ESM.zip › Figure EV3/panel K/Zygote.tif]

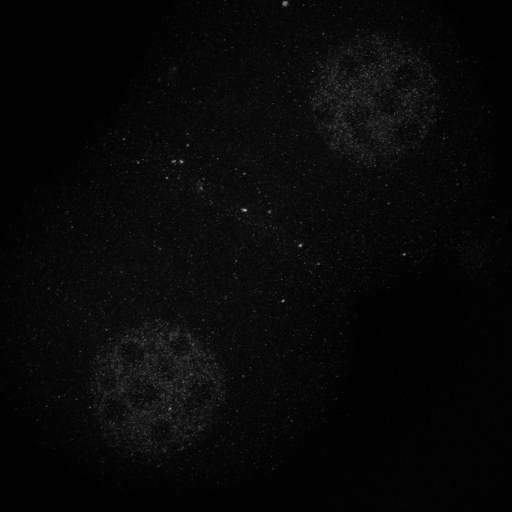

Supplement: Supplementary file 13 — Figure EV3 Source Data [file 44318_2026_736_MOESM13_ESM.zip › Figure EV3/panel K/Late 2-cell.tif]

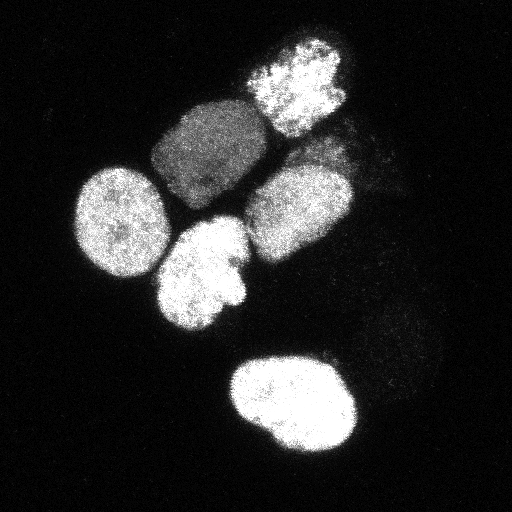

Supplement: Supplementary file 13 — Figure EV3 Source Data [file 44318_2026_736_MOESM13_ESM.zip › Figure EV3/panel M/ESC_Controls_CRX.tif]

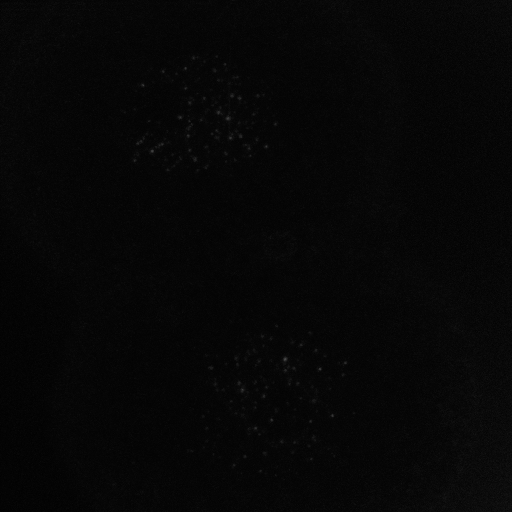

Supplement: Supplementary file 13 — Figure EV3 Source Data [file 44318_2026_736_MOESM13_ESM.zip › Figure EV3/panel I/Early 2-cell.tif]

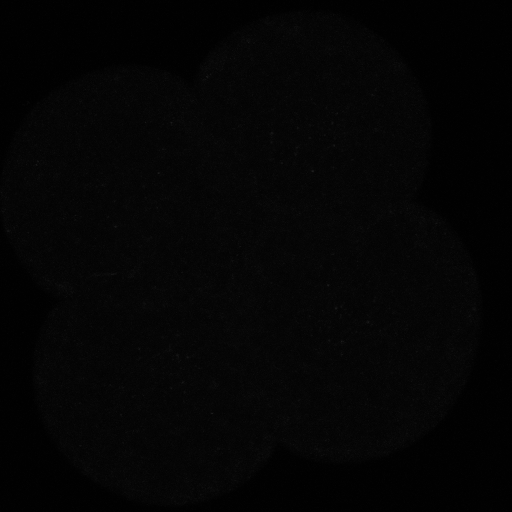

Supplement: Supplementary file 13 — Figure EV3 Source Data [file 44318_2026_736_MOESM13_ESM.zip › Figure EV3/panel I/4-cell.tif]

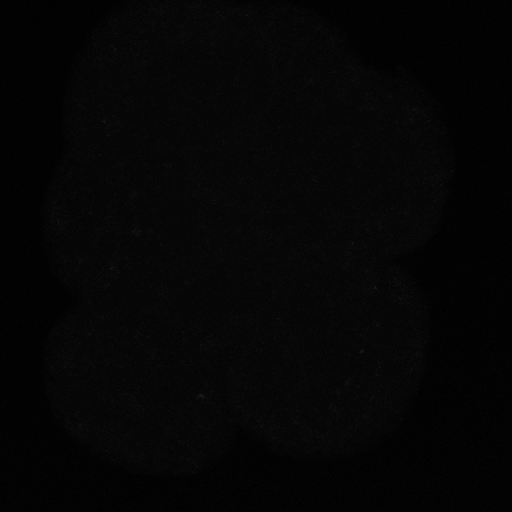

Supplement: Supplementary file 13 — Figure EV3 Source Data [file 44318_2026_736_MOESM13_ESM.zip › Figure EV3/panel I/8-cell.tif]

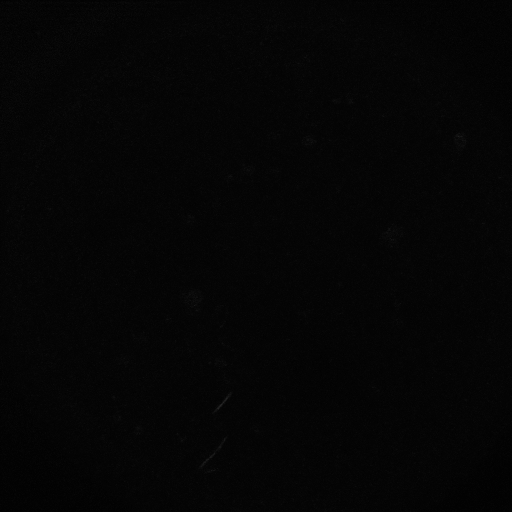

Supplement: Supplementary file 13 — Figure EV3 Source Data [file 44318_2026_736_MOESM13_ESM.zip › Figure EV3/panel I/Zygote.tif]

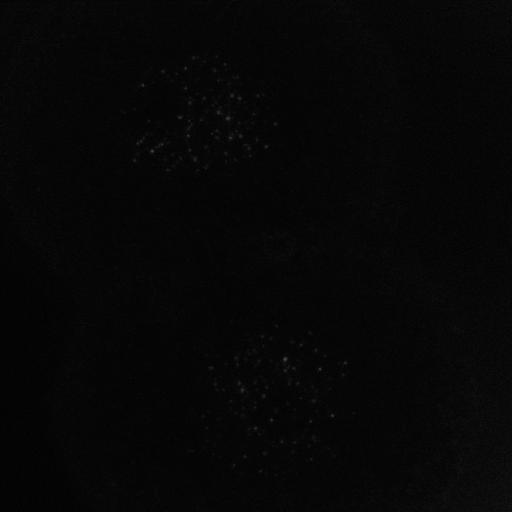

Supplement: Supplementary file 13 — Figure EV3 Source Data [file 44318_2026_736_MOESM13_ESM.zip › Figure EV3/panel I/Late 2-cell.tif]

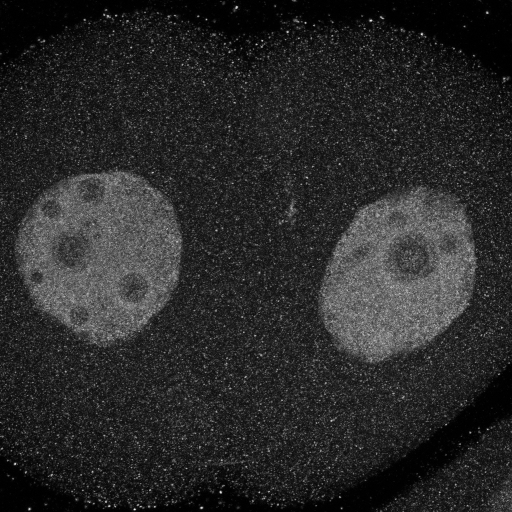

Supplement: Supplementary file 13 — Figure EV3 Source Data [file 44318_2026_736_MOESM13_ESM.zip › Figure EV3/panel J/LMX1A/Early 2-cell_LMX1A.tif]

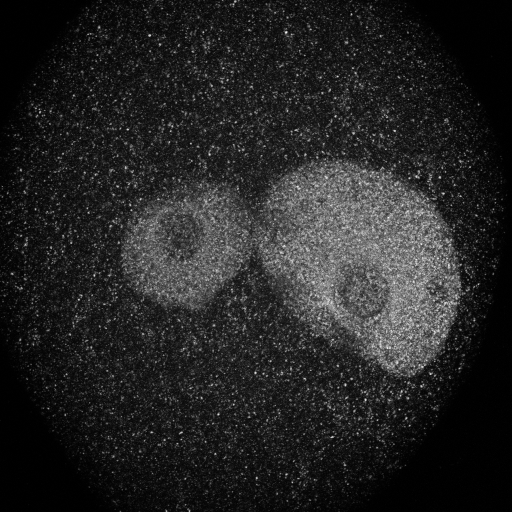

Supplement: Supplementary file 13 — Figure EV3 Source Data [file 44318_2026_736_MOESM13_ESM.zip › Figure EV3/panel J/LMX1A/zygote_LMX1A.tif]

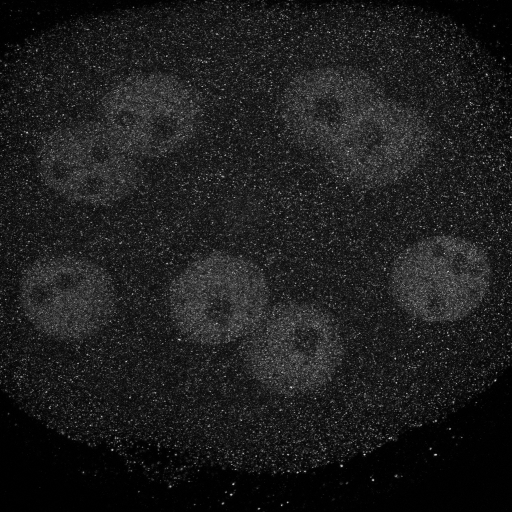

Supplement: Supplementary file 13 — Figure EV3 Source Data [file 44318_2026_736_MOESM13_ESM.zip › Figure EV3/panel J/LMX1A/8-cell_LMX1A.tif]

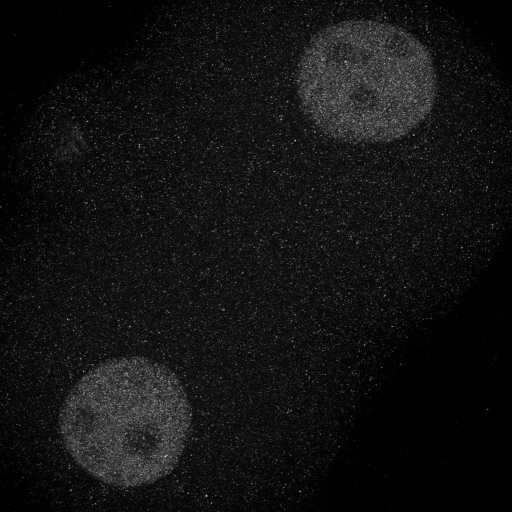

Supplement: Supplementary file 13 — Figure EV3 Source Data [file 44318_2026_736_MOESM13_ESM.zip › Figure EV3/panel J/LMX1A/Late 2-cell_LMX1A.tif]

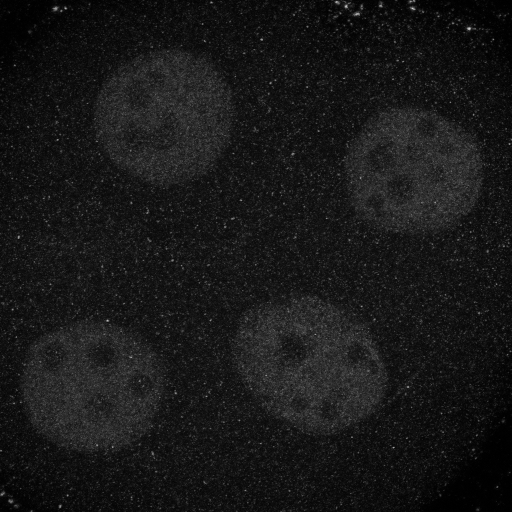

Supplement: Supplementary file 13 — Figure EV3 Source Data [file 44318_2026_736_MOESM13_ESM.zip › Figure EV3/panel J/LMX1A/4-cell_LMX1A.tif]

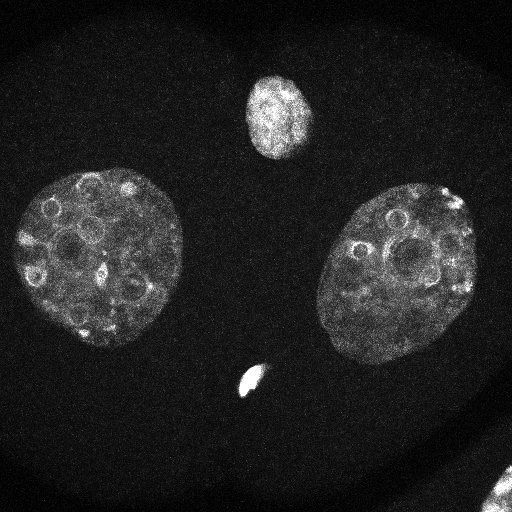

Supplement: Supplementary file 13 — Figure EV3 Source Data [file 44318_2026_736_MOESM13_ESM.zip › Figure EV3/panel J/DAPI/Early 2-cell_DAPI.tif]

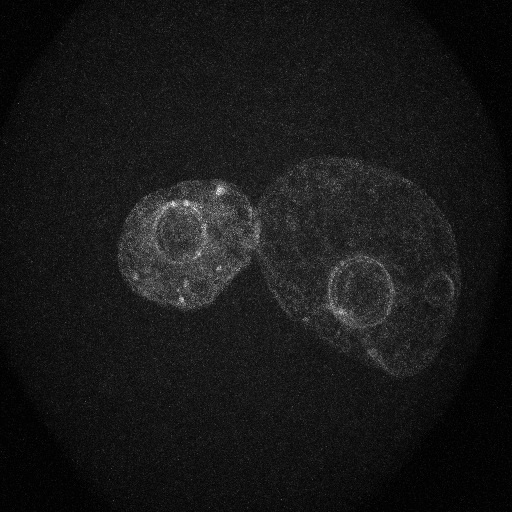

Supplement: Supplementary file 13 — Figure EV3 Source Data [file 44318_2026_736_MOESM13_ESM.zip › Figure EV3/panel J/DAPI/zygote_DAPI.tif]

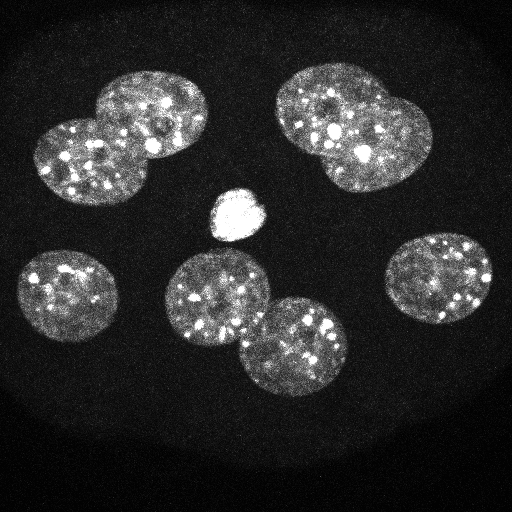

Supplement: Supplementary file 13 — Figure EV3 Source Data [file 44318_2026_736_MOESM13_ESM.zip › Figure EV3/panel J/DAPI/8-cell_DAPI.tif]

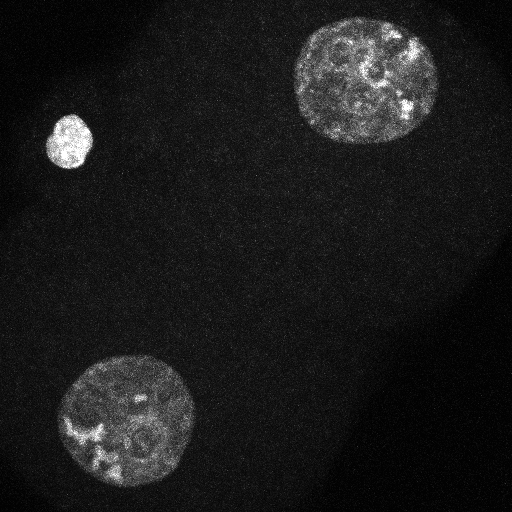

Supplement: Supplementary file 13 — Figure EV3 Source Data [file 44318_2026_736_MOESM13_ESM.zip › Figure EV3/panel J/DAPI/Late 2-cell_DAPI.tif]

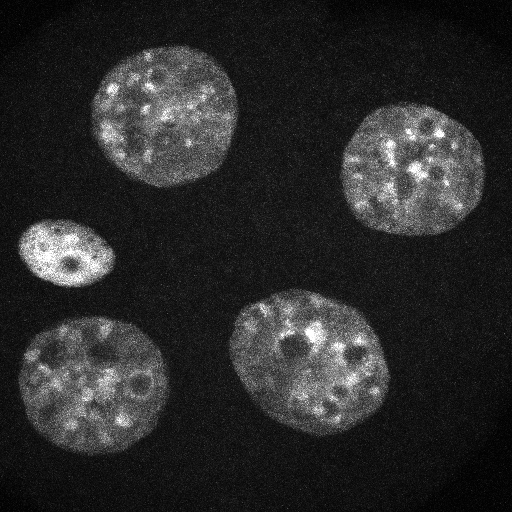

Supplement: Supplementary file 13 — Figure EV3 Source Data [file 44318_2026_736_MOESM13_ESM.zip › Figure EV3/panel J/DAPI/4-cell_DAPI.tif]

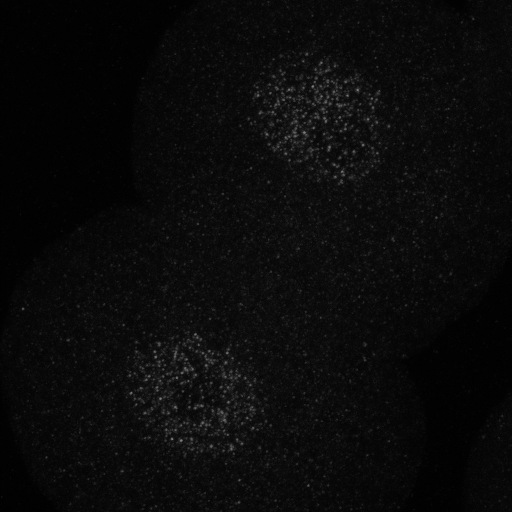

Supplement: Supplementary file 13 — Figure EV3 Source Data [file 44318_2026_736_MOESM13_ESM.zip › Figure EV3/panel H/RFX7/Early 2C_RFX7.tif]

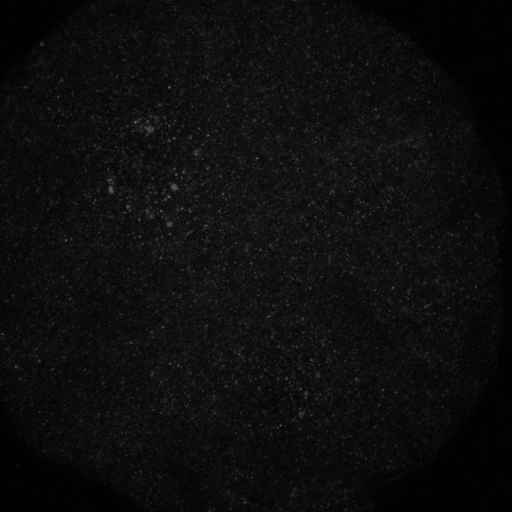

Supplement: Supplementary file 13 — Figure EV3 Source Data [file 44318_2026_736_MOESM13_ESM.zip › Figure EV3/panel H/RFX7/Zygote_RFX7.tif]

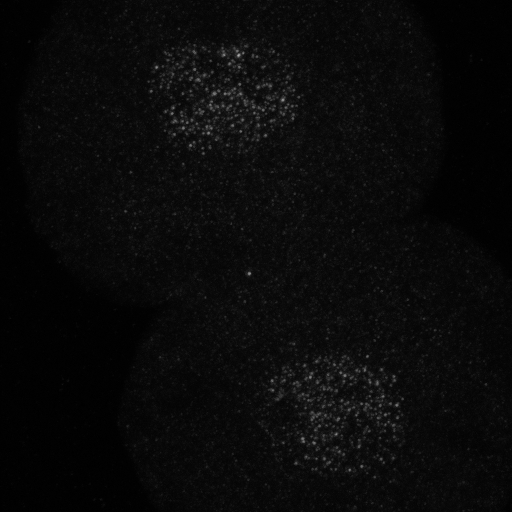

Supplement: Supplementary file 13 — Figure EV3 Source Data [file 44318_2026_736_MOESM13_ESM.zip › Figure EV3/panel H/RFX7/Late 2C_RFX7.tif]

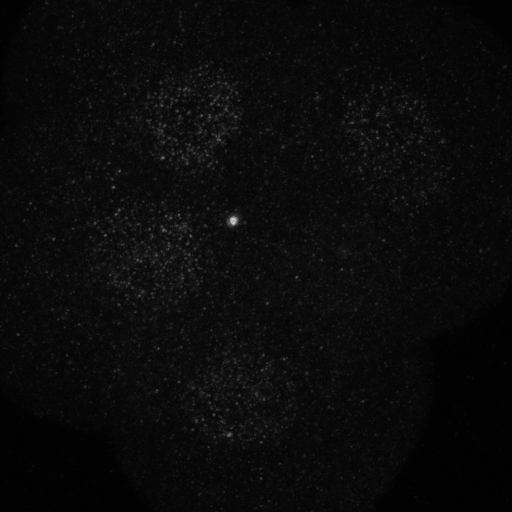

Supplement: Supplementary file 13 — Figure EV3 Source Data [file 44318_2026_736_MOESM13_ESM.zip › Figure EV3/panel H/RFX7/4C_RFX7.tif]

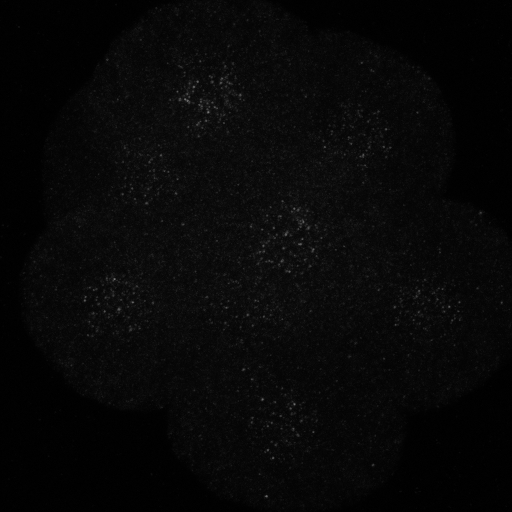

Supplement: Supplementary file 13 — Figure EV3 Source Data [file 44318_2026_736_MOESM13_ESM.zip › Figure EV3/panel H/RFX7/8C_RFX7.tif]

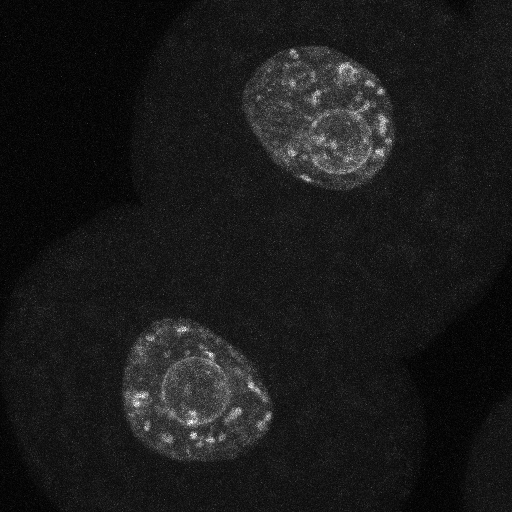

Supplement: Supplementary file 13 — Figure EV3 Source Data [file 44318_2026_736_MOESM13_ESM.zip › Figure EV3/panel H/DAPI/Early 2C_DAPI.tif]

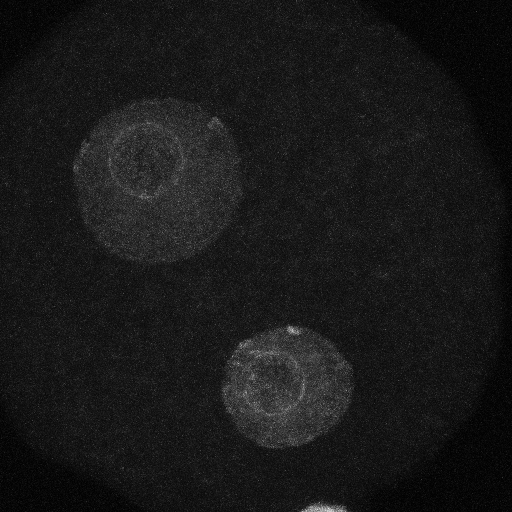

Supplement: Supplementary file 13 — Figure EV3 Source Data [file 44318_2026_736_MOESM13_ESM.zip › Figure EV3/panel H/DAPI/Zygote_DAPI.tif]

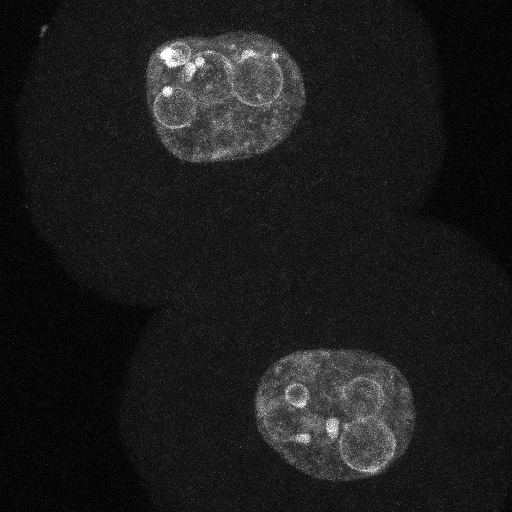

Supplement: Supplementary file 13 — Figure EV3 Source Data [file 44318_2026_736_MOESM13_ESM.zip › Figure EV3/panel H/DAPI/Late 2C_DAPI.tif]

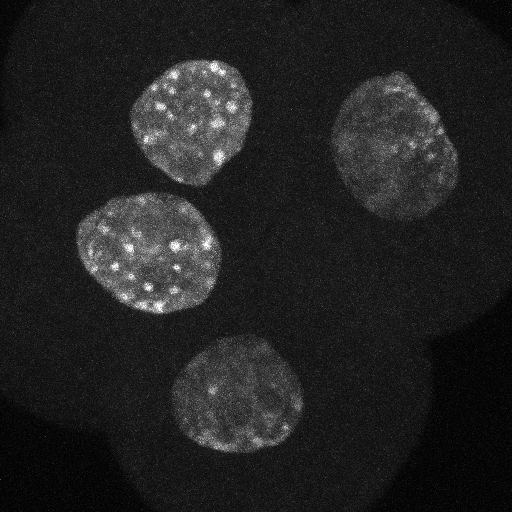

Supplement: Supplementary file 13 — Figure EV3 Source Data [file 44318_2026_736_MOESM13_ESM.zip › Figure EV3/panel H/DAPI/4C_DAPI.tif]

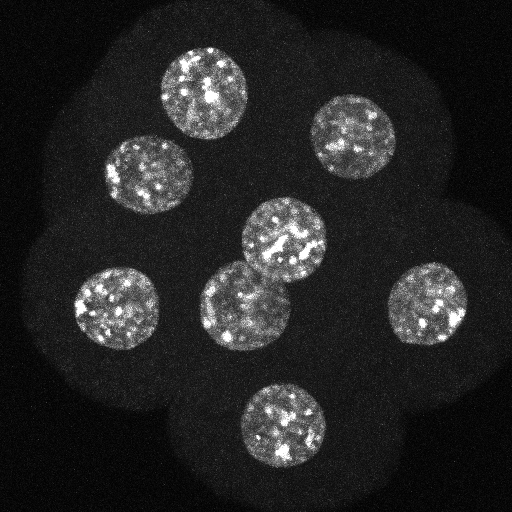

Supplement: Supplementary file 13 — Figure EV3 Source Data [file 44318_2026_736_MOESM13_ESM.zip › Figure EV3/panel H/DAPI/8C_DAPI.tif]

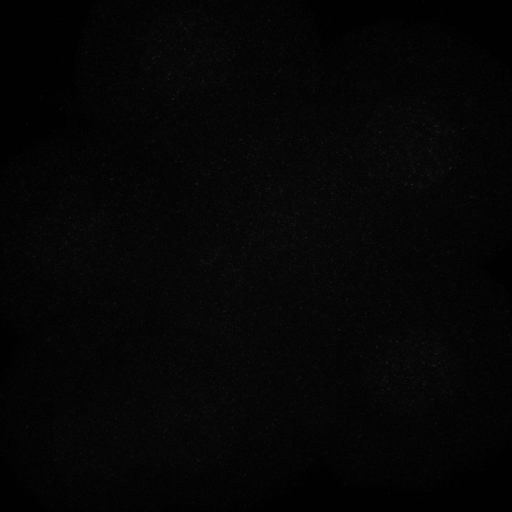

Supplement: Supplementary file 13 — Figure EV3 Source Data [file 44318_2026_736_MOESM13_ESM.zip › Figure EV3/panel G/SMAD3/8-cell_SMAD3.tif]

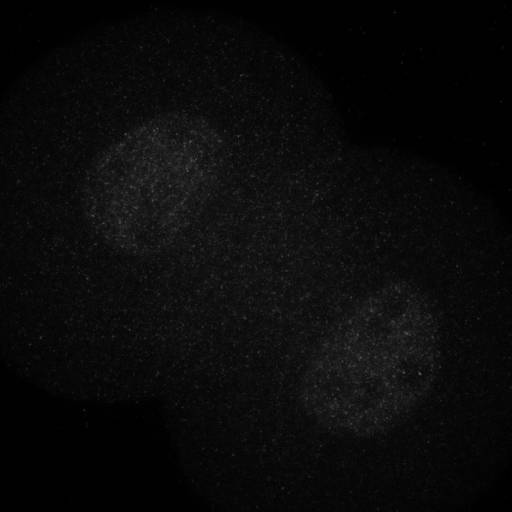

Supplement: Supplementary file 13 — Figure EV3 Source Data [file 44318_2026_736_MOESM13_ESM.zip › Figure EV3/panel G/SMAD3/Late 2-cell_SMAD3.tif]

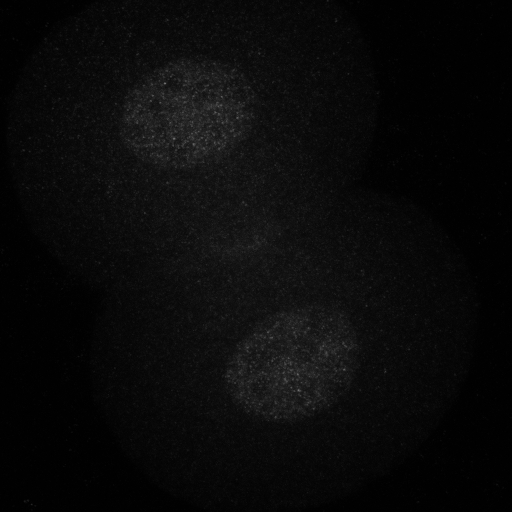

Supplement: Supplementary file 13 — Figure EV3 Source Data [file 44318_2026_736_MOESM13_ESM.zip › Figure EV3/panel G/SMAD3/Early 2-cell_SMAD3.tif]

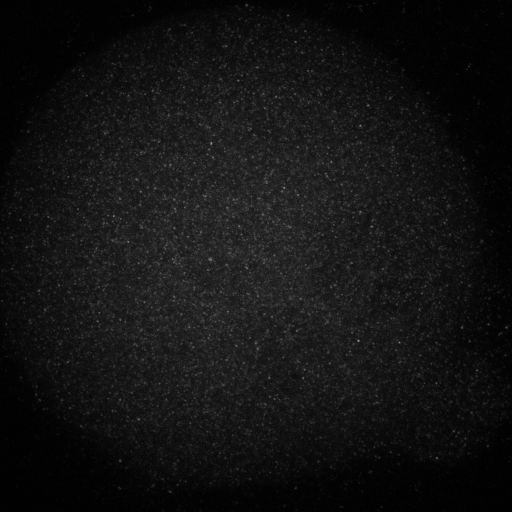

Supplement: Supplementary file 13 — Figure EV3 Source Data [file 44318_2026_736_MOESM13_ESM.zip › Figure EV3/panel G/SMAD3/Zygote_SMAD3.tif]

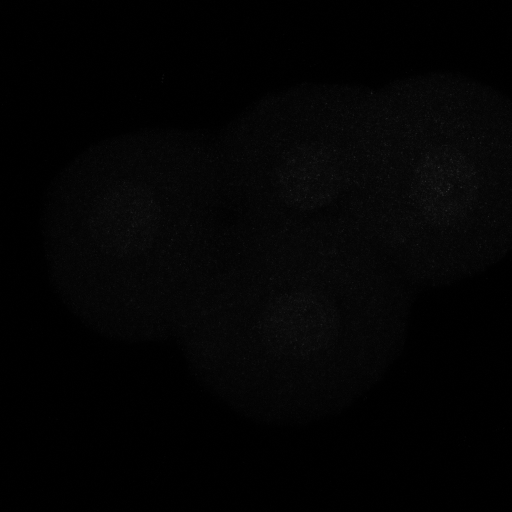

Supplement: Supplementary file 13 — Figure EV3 Source Data [file 44318_2026_736_MOESM13_ESM.zip › Figure EV3/panel G/SMAD3/4-cell_SMAD3.tif]

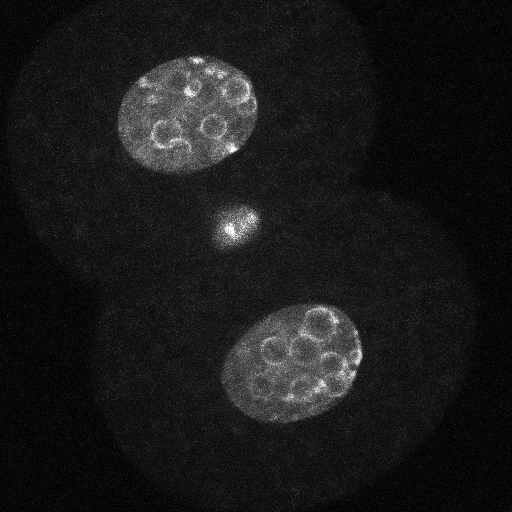

Supplement: Supplementary file 13 — Figure EV3 Source Data [file 44318_2026_736_MOESM13_ESM.zip › Figure EV3/panel G/DAPI/Early 2-cell_DAPI.tif]

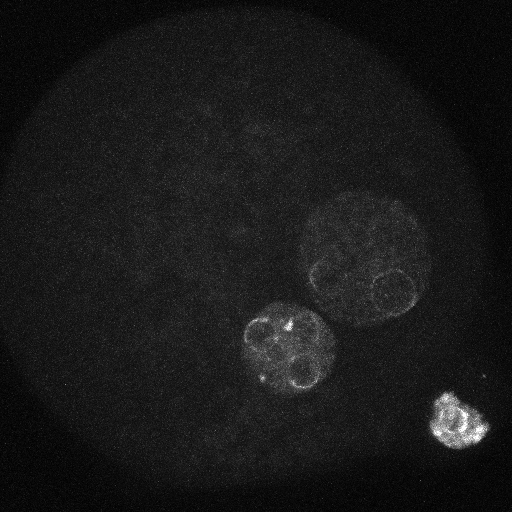

Supplement: Supplementary file 13 — Figure EV3 Source Data [file 44318_2026_736_MOESM13_ESM.zip › Figure EV3/panel G/DAPI/zygote_DAPI.tif]

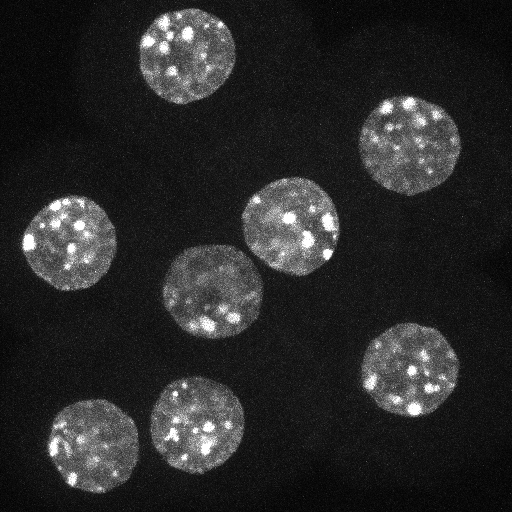

Supplement: Supplementary file 13 — Figure EV3 Source Data [file 44318_2026_736_MOESM13_ESM.zip › Figure EV3/panel G/DAPI/8-cell_DAPI.tif]

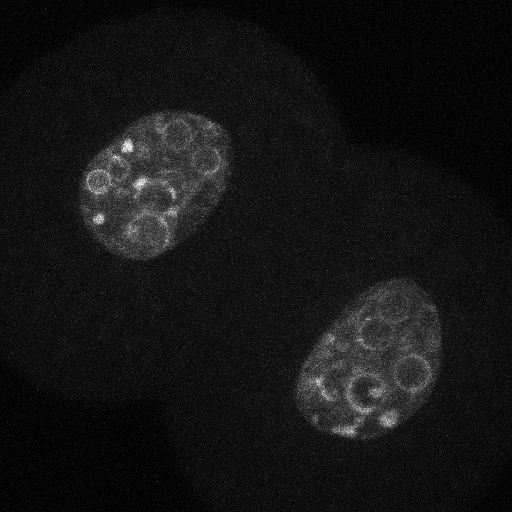

Supplement: Supplementary file 13 — Figure EV3 Source Data [file 44318_2026_736_MOESM13_ESM.zip › Figure EV3/panel G/DAPI/Late 2-cell_DAPI.tif]

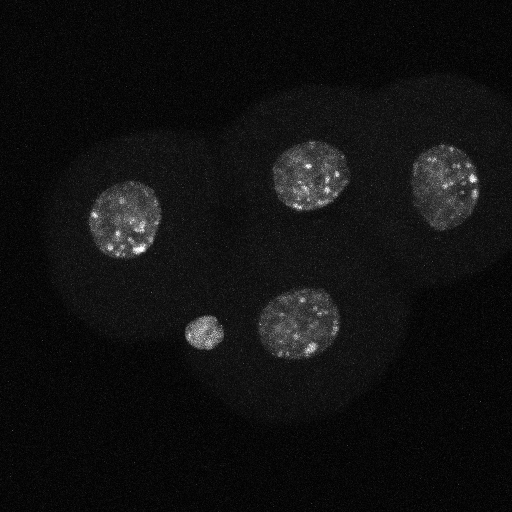

Supplement: Supplementary file 13 — Figure EV3 Source Data [file 44318_2026_736_MOESM13_ESM.zip › Figure EV3/panel G/DAPI/4-cell_DAPI.tif]

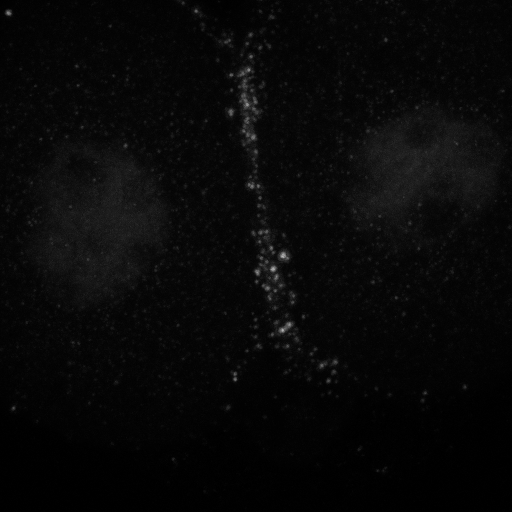

Supplement: Supplementary file 14 — Figure EV4 Source Data [file 44318_2026_736_MOESM14_ESM.zip › Figure EV4/Panel B/CONTROL.tif]

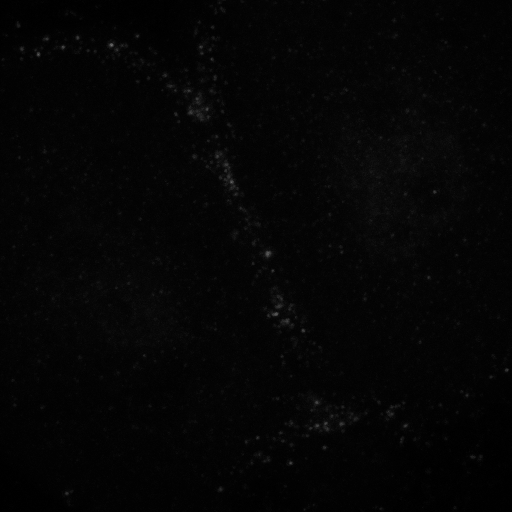

Supplement: Supplementary file 14 — Figure EV4 Source Data [file 44318_2026_736_MOESM14_ESM.zip › Figure EV4/Panel B/TBP LOF.tif]

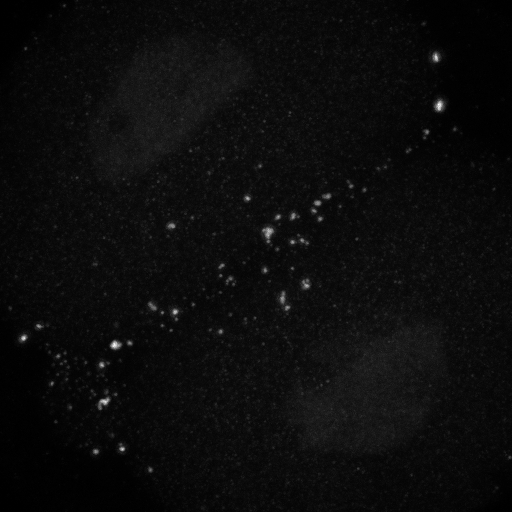

Supplement: Supplementary file 14 — Figure EV4 Source Data [file 44318_2026_736_MOESM14_ESM.zip › Figure EV4/Panel C/CONTROL.tif]

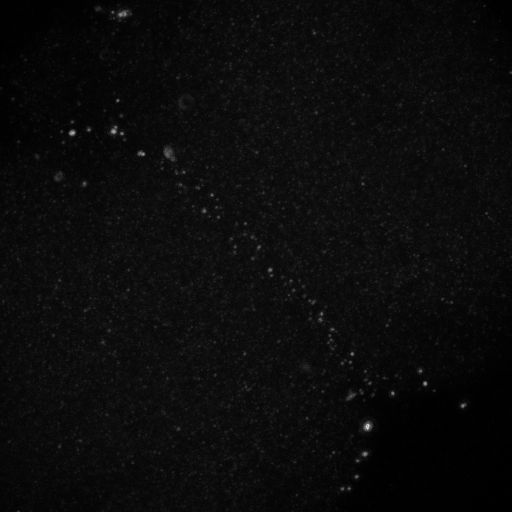

Supplement: Supplementary file 14 — Figure EV4 Source Data [file 44318_2026_736_MOESM14_ESM.zip › Figure EV4/Panel C/FOXJ3 LOF.tif]

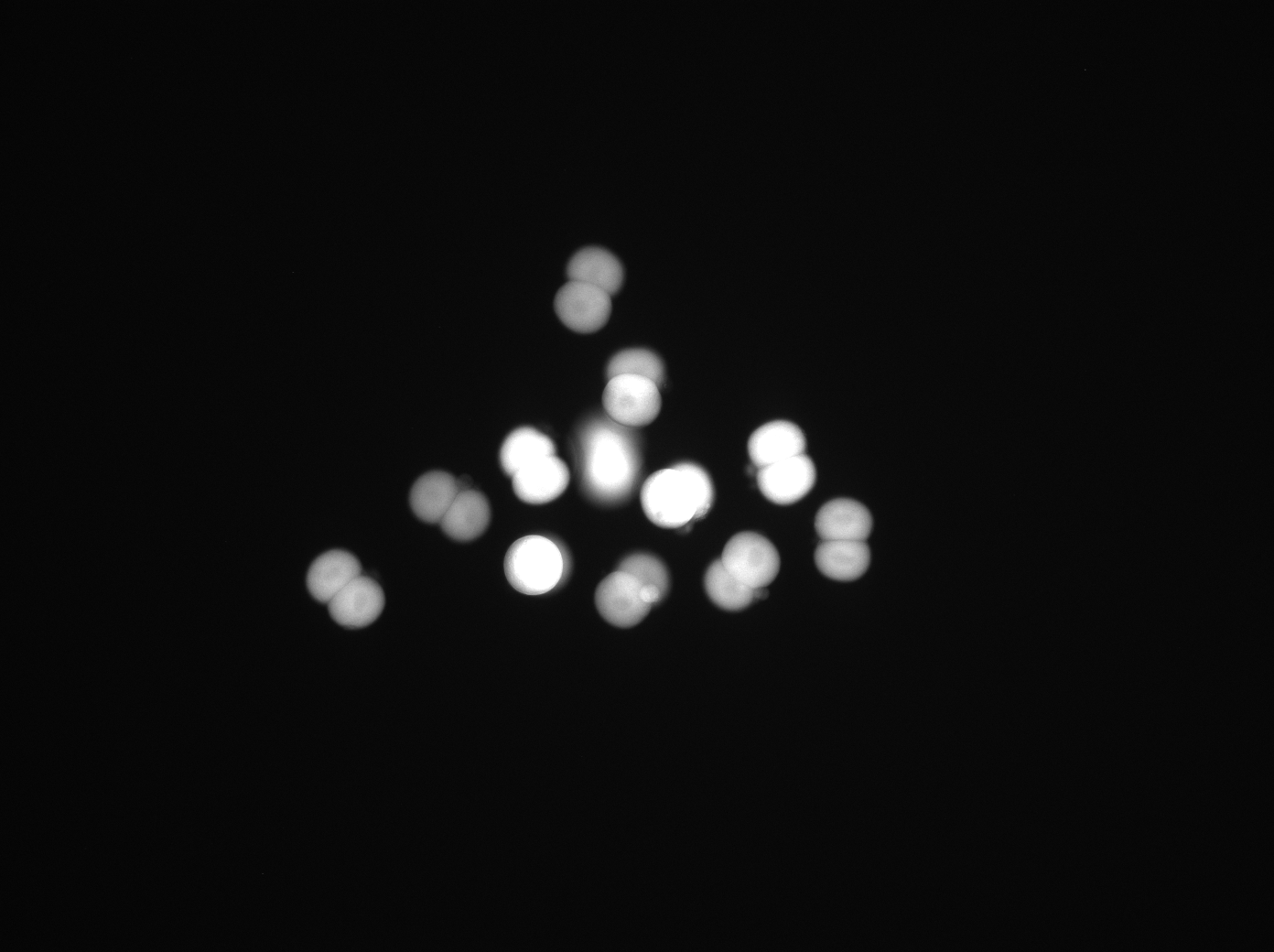

Supplement: Supplementary file 14 — Figure EV4 Source Data [file 44318_2026_736_MOESM14_ESM.zip › Figure EV4/Panel A/TBP LOF/mCherry-Trim21.tif]

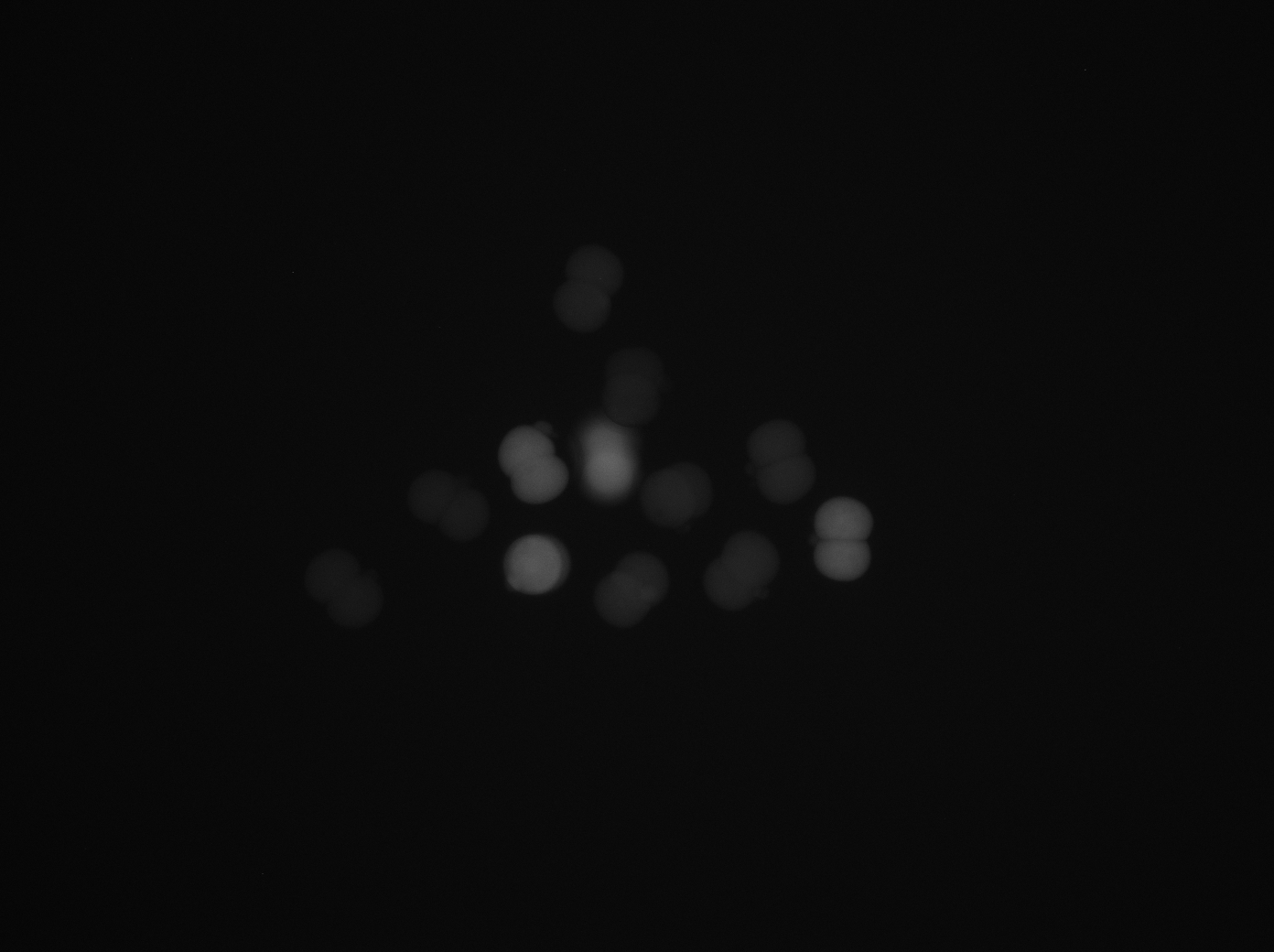

Supplement: Supplementary file 14 — Figure EV4 Source Data [file 44318_2026_736_MOESM14_ESM.zip › Figure EV4/Panel A/TBP LOF/Cascade Blue Dextran.tif]

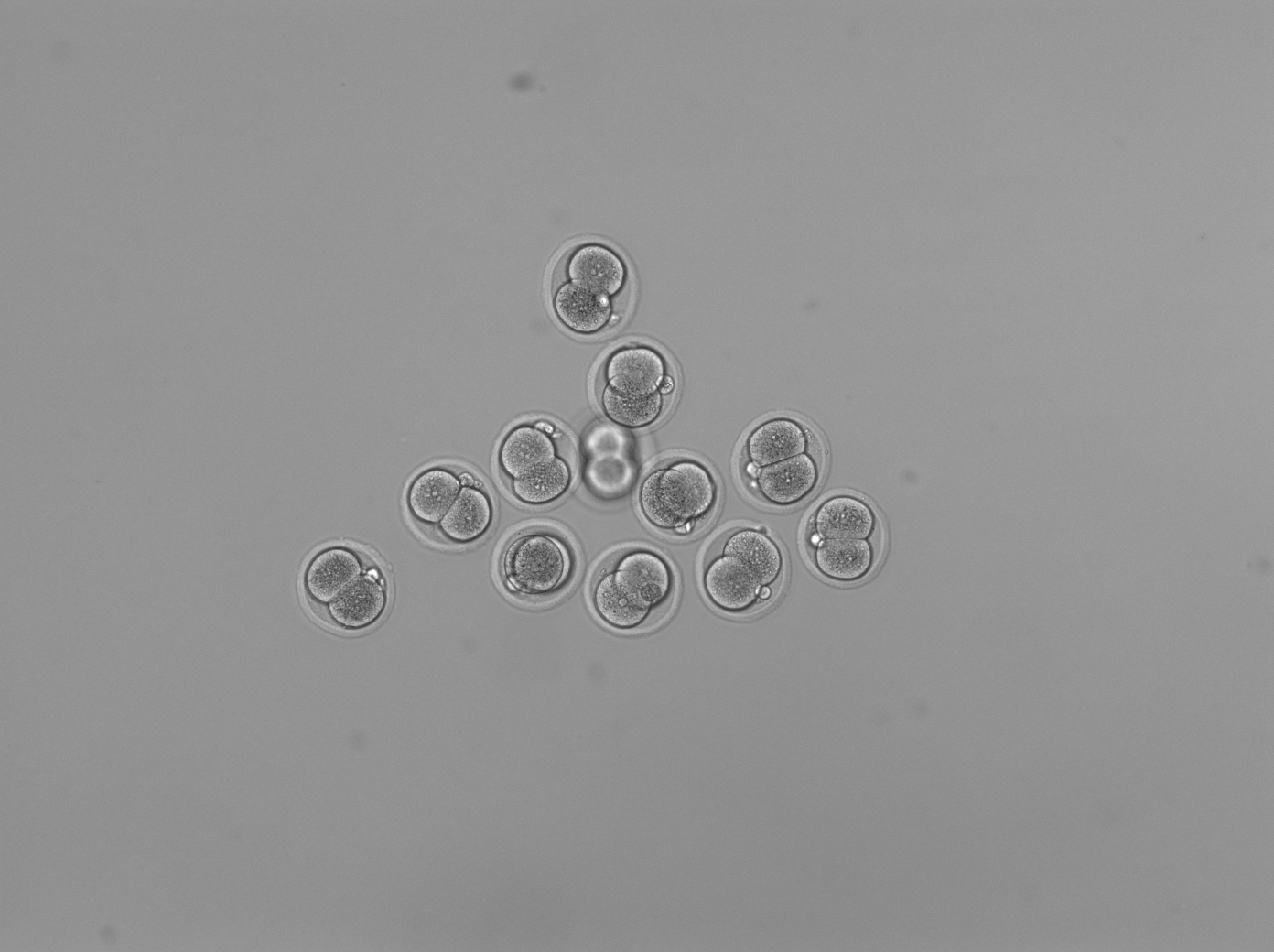

Supplement: Supplementary file 14 — Figure EV4 Source Data [file 44318_2026_736_MOESM14_ESM.zip › Figure EV4/Panel A/TBP LOF/Bright Field.tif]

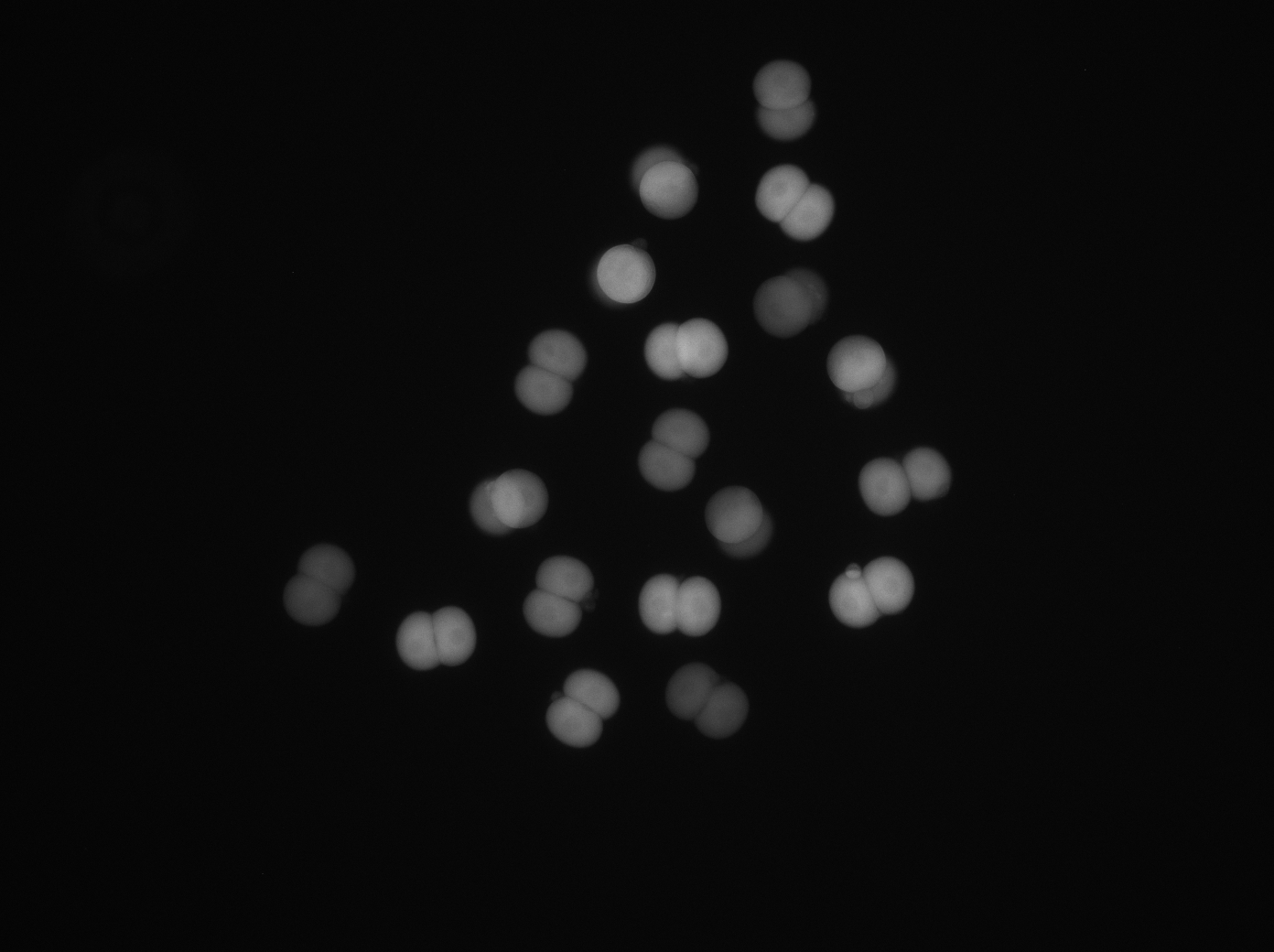

Supplement: Supplementary file 14 — Figure EV4 Source Data [file 44318_2026_736_MOESM14_ESM.zip › Figure EV4/Panel A/CONTROL/mCherry-Trim21.tif]

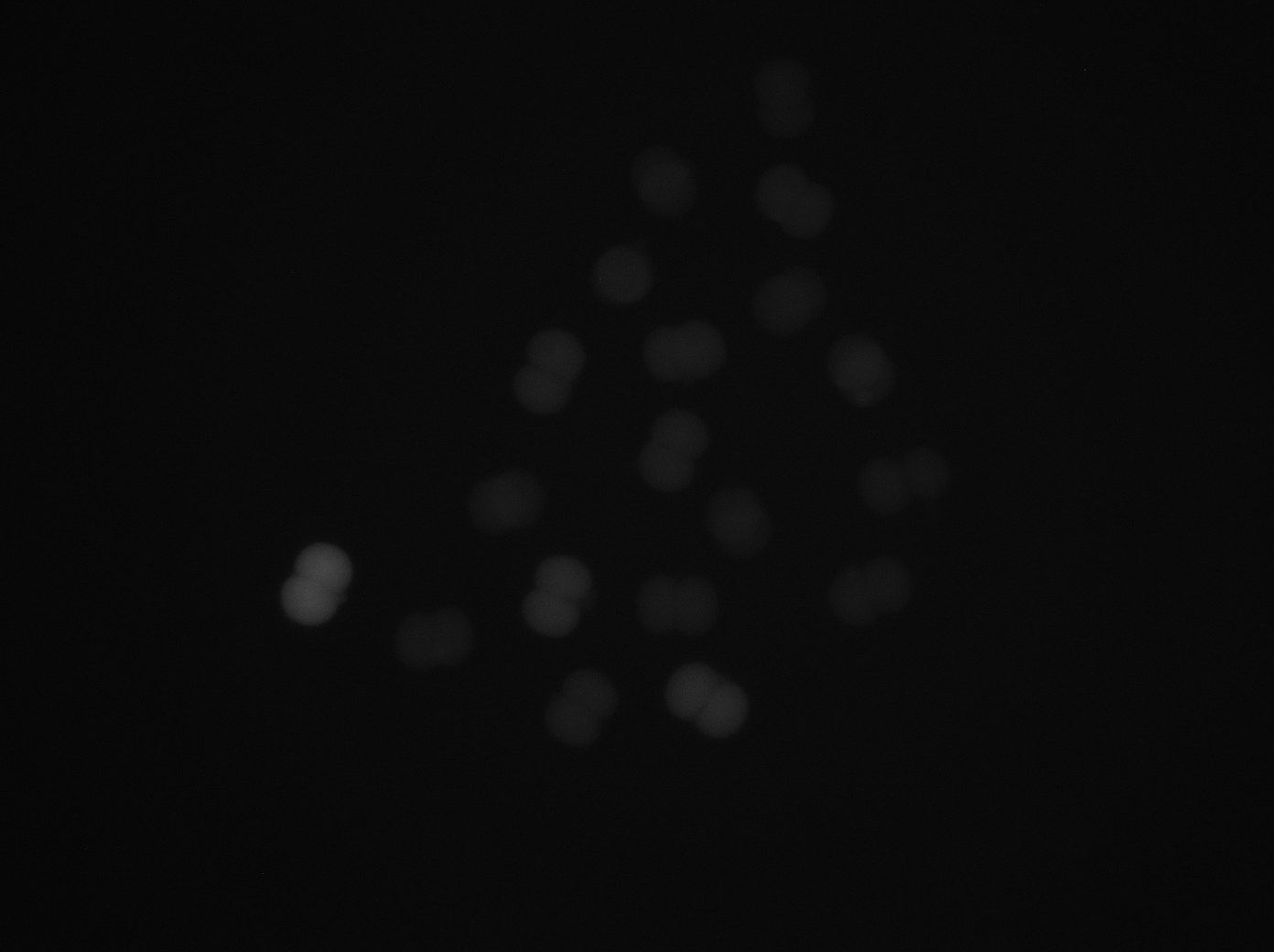

Supplement: Supplementary file 14 — Figure EV4 Source Data [file 44318_2026_736_MOESM14_ESM.zip › Figure EV4/Panel A/CONTROL/Cascade Blue Dextran.tif]

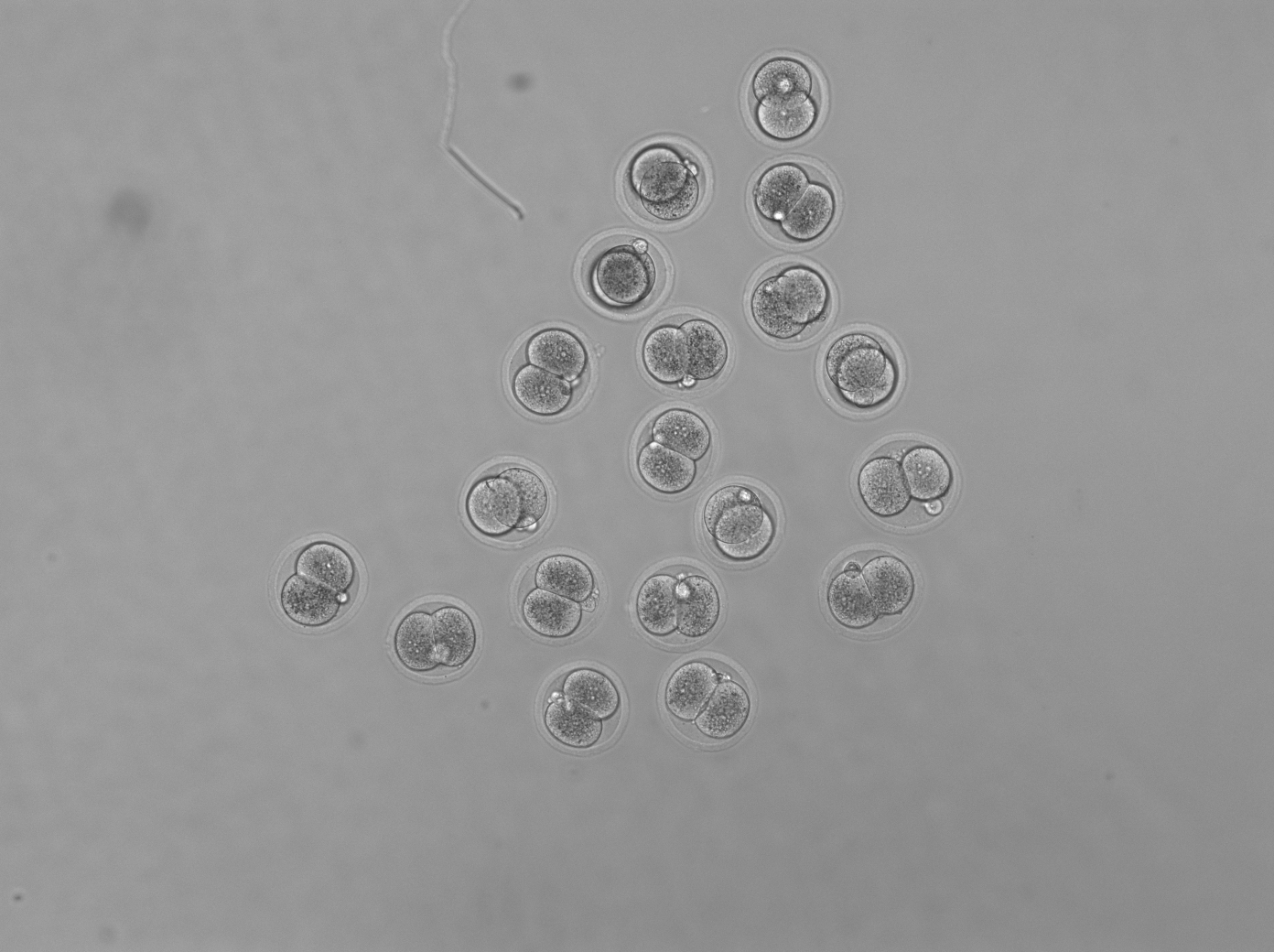

Supplement: Supplementary file 14 — Figure EV4 Source Data [file 44318_2026_736_MOESM14_ESM.zip › Figure EV4/Panel A/CONTROL/Bright Field.tif]
