# Supplementary material for: TBP regulates transposable element expression in early mouse embryos
Source: EMBO J. 2026 Mar 20;45(9):3073–101. doi: 10.1038/s44318-026-00736-w (PMC13144441; doi:10.1038/s44318-026-00736-w)
Supplement: Supplementary file 15 — Expanded View Figures [file 44318_2026_736_MOESM15_ESM.pdf]

Expanded View Figures

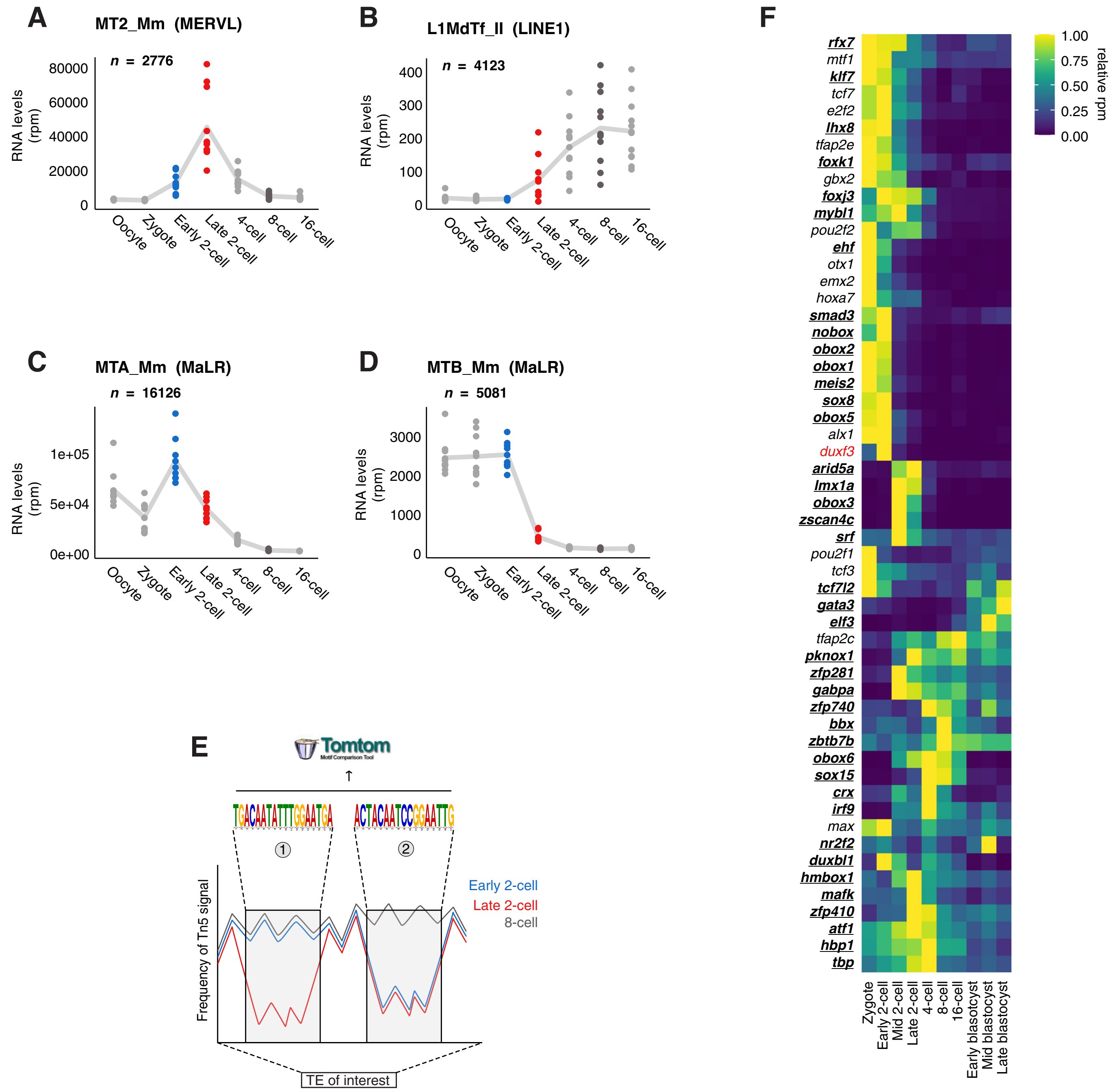

Figure EV1. Footprinting analysis identifies 54 putative regulators of TE expression.

(**A–D**) Expression levels of all MT2_Mm (**A**), L1MdTf_II monomers (**B**), MTA_Mm (**C**) and MTB_Mm (**D**) across preimplantation development. Each dot represents the sum rpm of all insertions belonging to each family of TE per single embryo at the indicated stage. *n* is the number of insertions per group. The trend line connects the mean values across embryos for each stage. (**E**) Schematic representation of the footprinting analysis. The coloured lines over the TE of interest represent the aggregated Tn5 insertion signal at the different stages. When a footprint is found by visual inspection to differ between stages, for example, the red line (late 2-cell stage) compared to the blue (early 2-cell stage) and grey (8-cell stage) lines at the same position of the TE, the sequence corresponding to those regions was extracted and subjected to motif search using the Tomtom tool of the MEME suite. (**F**) Heatmap showing the expression of the 54 candidate TFs (and *Duxf3*) across all the stages of preimplantation development. The values are the normalized counts centred on the row mean reanalysed from (Deng et al, 2014). TFs were ordered by hierarchical clustering. The TFs highlighted in bold and underlined correspond to the 40 TFs that were selected for functional studies.

 © The Author(s)

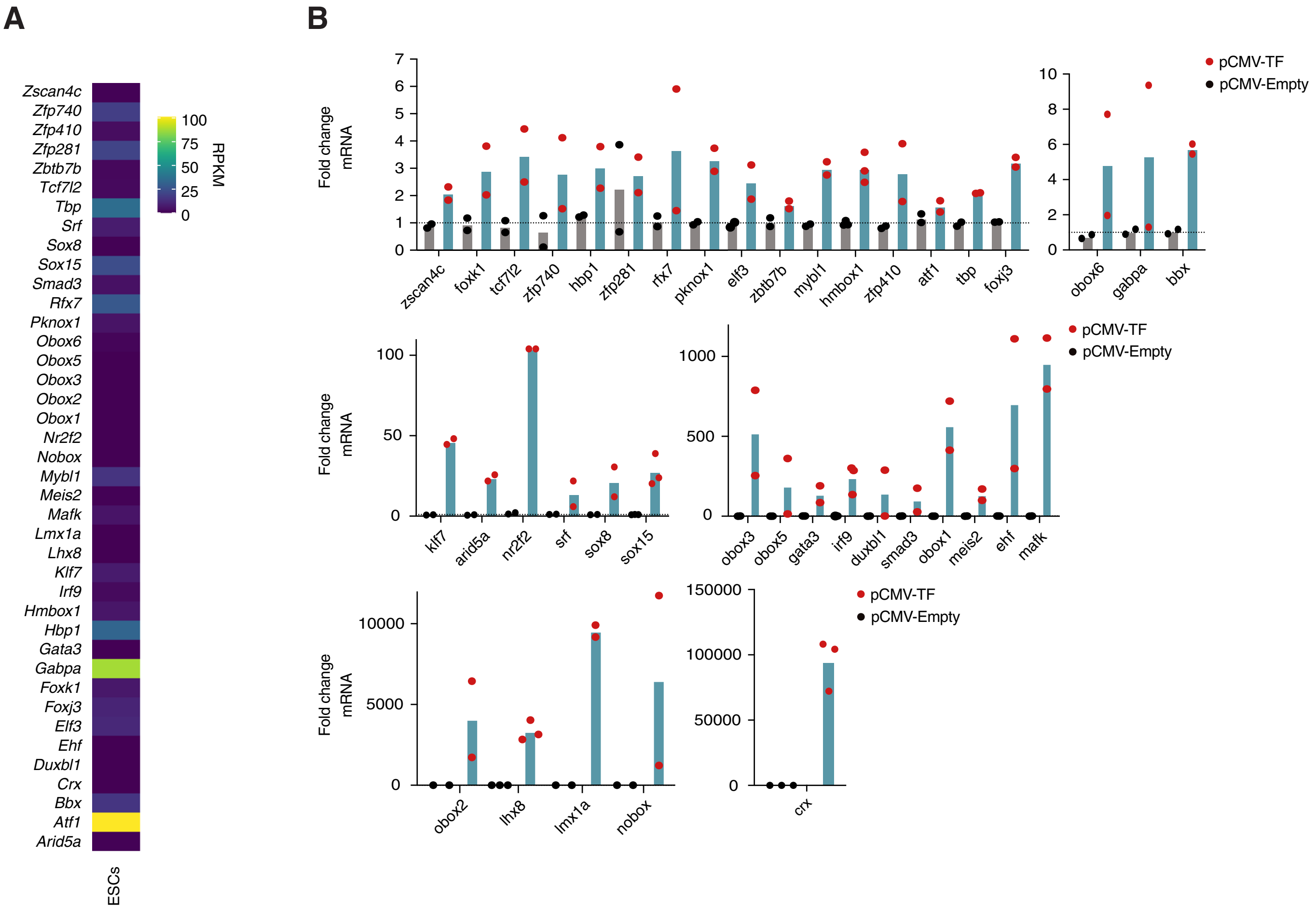

Figure EV2. A selection of TFs activates specific TE families upon overexpression in ESCs.

(**A**) Heatmap showing the expression of the 40 candidate TFs chosen for overexpression experiments in ESCs. The values are rpkm and derive from (Ishiuchi et al, 2015). (**B**) Fold change of the mRNA levels of the 40 TFs upon transfection of each of them over the corresponding empty-vector control by RT-qPCR. The bar represents the mean from two or three biological replicates, as indicated by the number of black and red dots, which represent the mean of three technical replicates for each biological replicate of cells transfected with pCMV-Empty and pCMV-TF, respectively. Source data are available online for this figure.

© The Author(s)

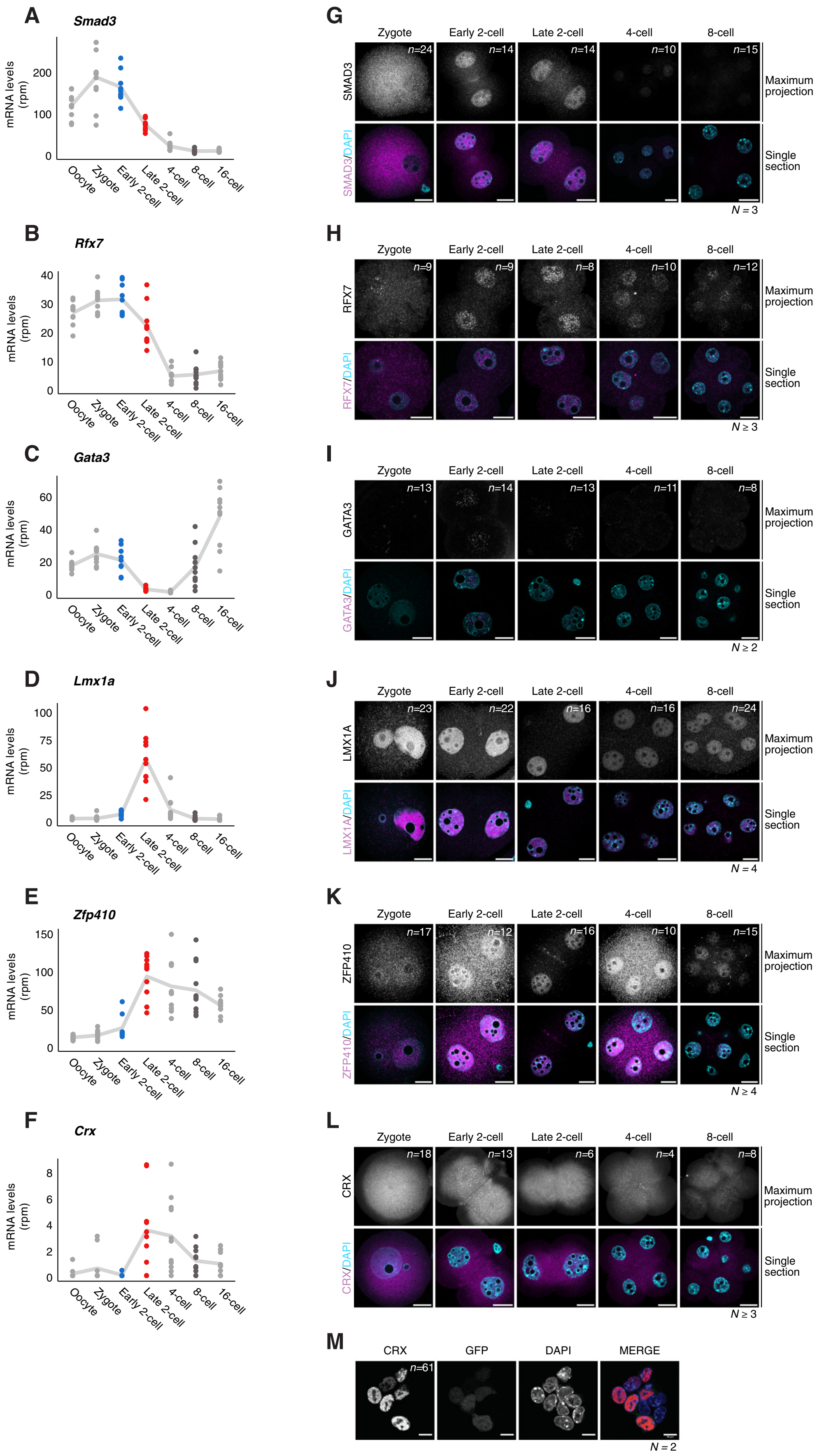
A
Smad3
mRNA levels (rpm)
200
100
0
Oocyte
Zygote
Early 2-cell
Late 2-cell
4-cell
8-cell
16-cell
B
Rfx7
40
30
20
10
0
C
Gata3
60
40
20
0
D
Lmx1a
100
75
50
25
0
E
Zfp410
150
100
50
0
F
Crx
8
6
4
2
0
G
Zygote
Early 2-cell
Late 2-cell
4-cell
8-cell
SMAD3
SMAD3/DAPI
n=24
n=14
n=14
n=10
n=15
Maximum projection
Single section
N = 3
H
RFX7
RFX7/DAPI
n=9
n=9
n=8
n=10
n=12
N ≥ 3
I
GATA3
GATA3/DAPI
n=13
n=14
n=13
n=11
n=8
N ≥ 2
J
LMX1A
LMX1A/DAPI
n=23
n=22
n=16
n=16
n=24
N = 4
K
ZFP410
ZFP410/DAPI
n=17
n=12
n=16
n=10
n=15
N ≥ 4
L
CRX
CRX/DAPI
n=18
n=13
n=6
n=4
n=8
N ≥ 3
M
CRX
GFP
DAPI
MERGE
n=61
N = 2

Figure EV3. A selection of TFs is expressed during preimplantation development.

(**A–F**) Expression of *Smad3* (**A**), *Rfx7* (**B**), *Gata3* (**C**), *Lmx1a* (**D**), *Zfp410* (**E**) and *Crx* (**F**) during mouse preimplantation development. Each dot represents the TF mRNA levels (rpm) in individual embryos at the indicated stages analysed from (Oomen et al, 2025). The trend line connects the mean values across embryos for each stage. (**G–L**) Representative images of SMAD3 (**G**), RFX7 (**H**), GATA3 (**I**), LMX1A (**J**), ZFP410 (**K**) and CRX (**L**) immunostainings at the indicated developmental stage. All embryos within each replicate were processed and acquired using the same conditions, hence the intensity of the fluorescent signal is comparable between all embryos. Top images are maximum intensity projection, bottom are merged images with DAPI staining shown as single confocal sections. *n* is the total number of embryos analysed per stage. *N*, the number of independent replicates. Scale bars, 20 µm. (**M**) Representative image of CRX immunostaining and GFP fluorescence (as a positive control for successful transfection) upon CRX overexpression in ESCs. *n* is the number of cells that were displaying both GFP and CRX signal. *N*, the number of independent replicates. Scale bars, 10 µm. Source data are available online for this figure.

© The Author(s)

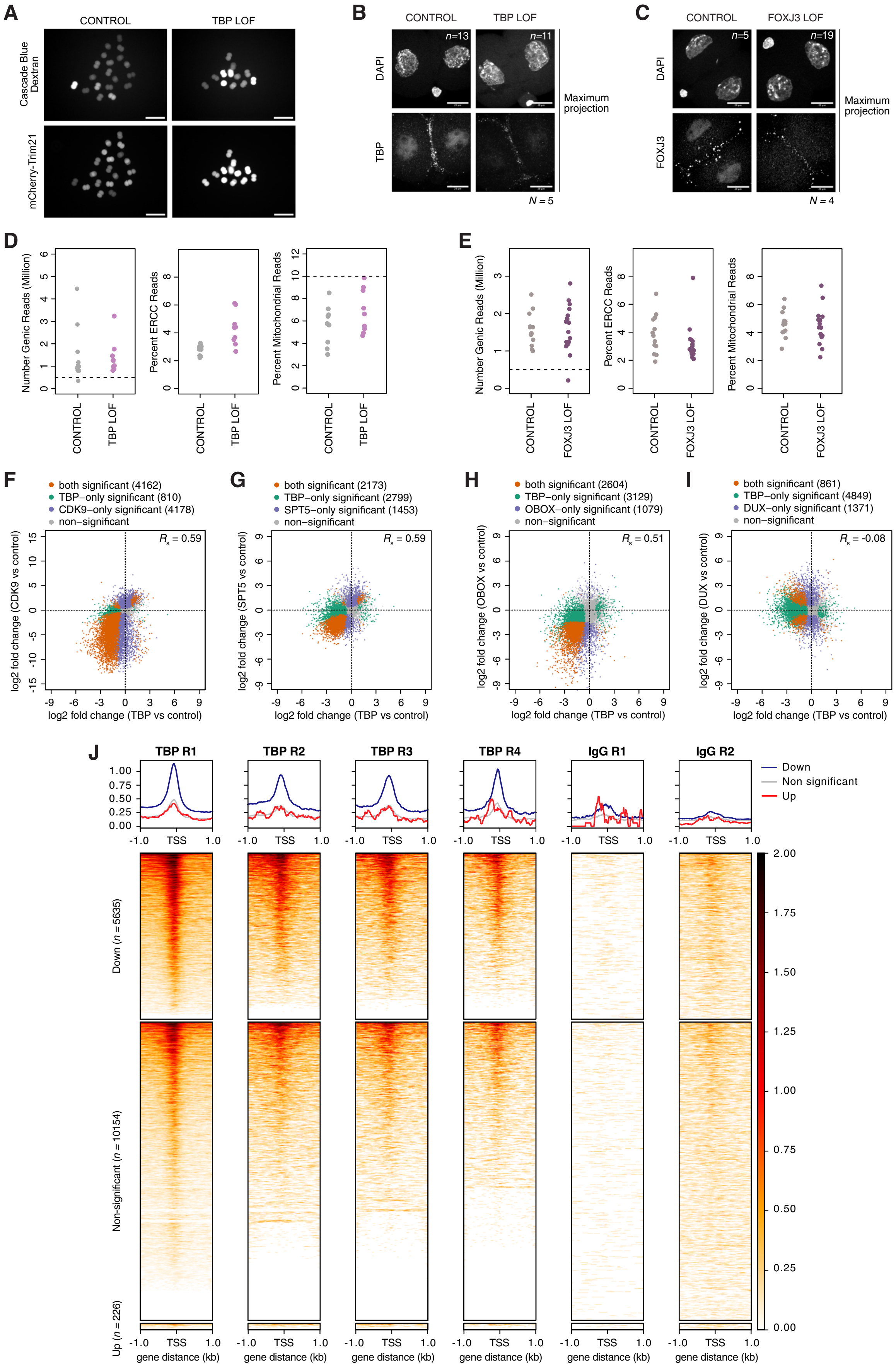
A
CONTROL
TBP LOF
Cascade Blue Dextran
mCherry-Trim21
B
CONTROL
TBP LOF
DAPI
TBP
n=13
n=11
Maximum projection
N = 5
C
CONTROL
FOXJ3 LOF
DAPI
FOXJ3
n=5
n=19
Maximum projection
N = 4
D
Number Genic Reads (Million)
Percent ERCC Reads
Percent Mitochondrial Reads
CONTROL
TBP LOF
E
Number Genic Reads (Million)
Percent ERCC Reads
Percent Mitochondrial Reads
CONTROL
FOXJ3 LOF
F
both significant (4162)
TBP–only significant (810)
CDK9–only significant (4178)
non–significant
R_s = 0.59
log2 fold change (CDK9 vs control)
log2 fold change (TBP vs control)
G
both significant (2173)
TBP–only significant (2799)
SPT5–only significant (1453)
non–significant
R_s = 0.59
log2 fold change (SPT5 vs control)
log2 fold change (TBP vs control)
H
both significant (2604)
TBP–only significant (3129)
OBOX–only significant (1079)
non–significant
R_s = 0.51
log2 fold change (OBOX vs control)
log2 fold change (TBP vs control)
I
both significant (861)
TBP–only significant (4849)
DUX–only significant (1371)
non–significant
R_s = -0.08
log2 fold change (DUX vs control)
log2 fold change (TBP vs control)
J
TBP R1
TBP R2
TBP R3
TBP R4
IgG R1
IgG R2
Down
Non significant
Up
-1.0 TSS 1.0
Down (n = 5635)
Non-significant (n = 10154)
Up (n = 226)
gene distance (kb)
2.00
1.75
1.50
1.25
1.00
0.75
0.50
0.25
0.00

 © The Author(s)

Figure EV4. Loss of TBP impairs ZGA and delays developmental progression.

(**A**) Representative images of late 2-cell stage embryos after sequential microinjection with TBP antibody or IgG antibody together with cascade blue-coupled dextran and mCherry-Trim21. All embryos were examined for cascade blue and mCherry fluorescence in all the experiments performed throughout the manuscript. Scale bar, 200 µm. (**B**, **C**) Representative images of TBP (**B**) and FOXJ3 (**C**) immunostainings following acute protein depletion by Trim-Away. Left images are from either TBP or FOXJ3 LOF, right images correspond to the respective CONTROL embryos (see Fig. 3A). All images shown are maximum intensity projections. Top is DAPI signal, bottom is either TBP or FOXJ3 staining. *n* is the total number of embryos analysed per condition. *N*, the number of independent replicates. Scale bars, 20 µm. (**D**, **E**) Dot plots showing single embryo RNA-sequencing quality controls of TBP LOF and CONTROL embryos (**D**), and FOXJ3 LOF and CONTROL embryos (**E**). The number of genic reads, the percentage of ERCC reads and the percentage of reads mapping to mitochondrial genes in embryos are shown. Each dot is a single embryo, the dashed lines represent the thresholds applied. (**F–I**) Scatterplots of RNA-seq log2 fold change differences between CDK9 inhibition versus control (**F**) (Abe et al, 2022), SPT5 Trim-Away versus control (**G**) (Abe et al, 2022), OBOX knock-out versus control (**H**) (Ji et al, 2023) and DUX knock-down versus control (**I**) (Hermant et al, 2025) against TBP LOF relative to CONTROL. Genes significantly differentially expressed in TBP LOF only are depicted in green, those in CDK9, SPT5 or DUX LOF only are displayed in purple, and differentially expressed genes in both conditions are displayed in orange. Spearman's correlations (R_s) are indicated. (**J**) Signal aggregate plots and heatmaps of TBP enrichment at the late 2-cell stage from the four individual CUT&Tag replicates or IgG control from two individual replicates over the TSS of down-regulated (padj < 0.05), non-significant and up-regulated (padj < 0.05) genes upon TBP LOF relative to CONTROL embryos. *n* is the number of genes per category indicated. Source data are available online for this figure.

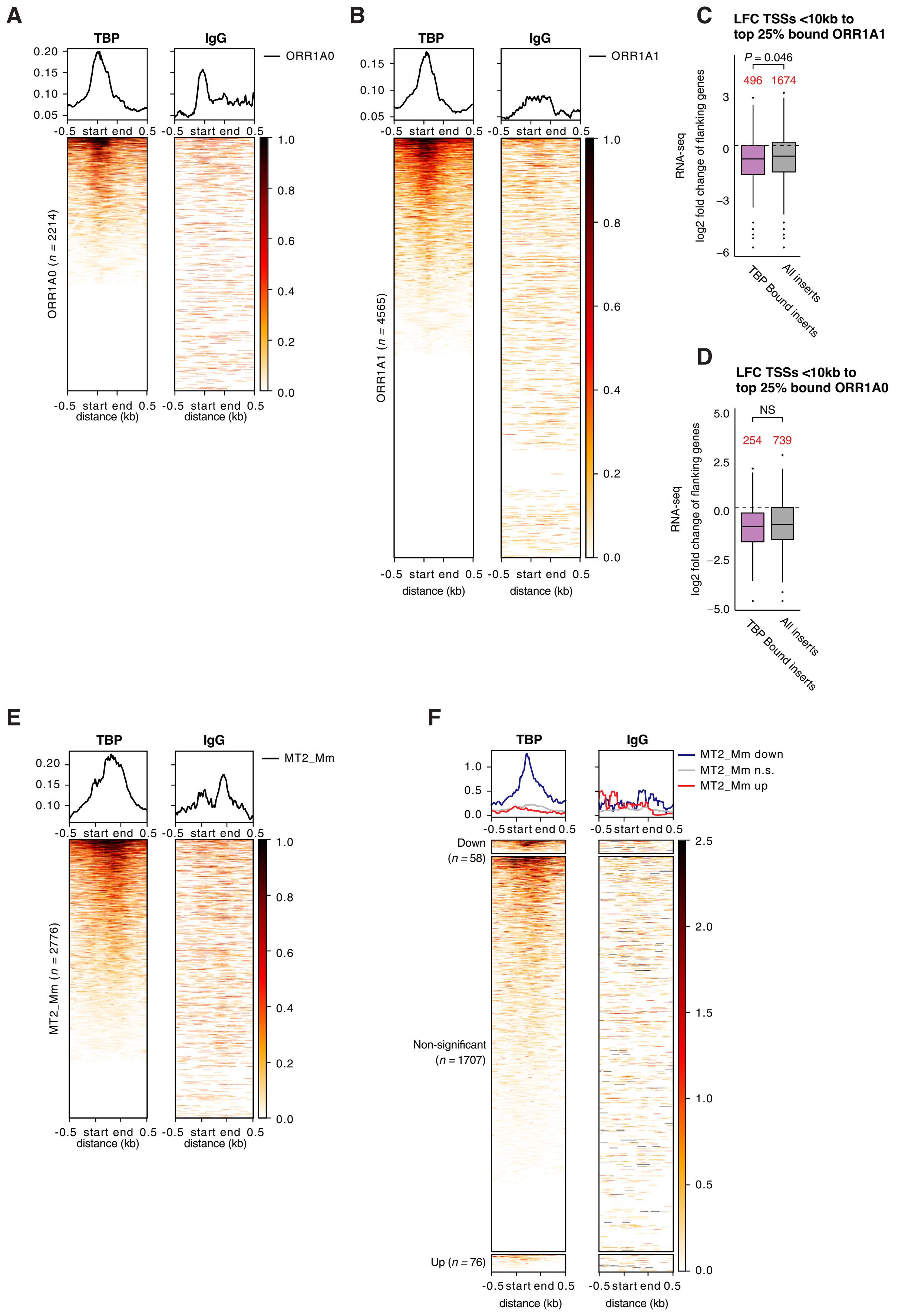
A
TBP
IgG
ORR1A0
ORR1A0 (n = 2214)
-0.5 start end 0.5
distance (kb)
B
TBP
IgG
ORR1A1
ORR1A1 (n = 4565)
-0.5 start end 0.5
distance (kb)
C
LFC TSSs <10kb to top 25% bound ORR1A1
P = 0.046
496 1674
RNA-seq
log2 fold change of flanking genes
TBP Bound inserts
All inserts
D
LFC TSSs <10kb to top 25% bound ORR1A0
NS
254 739
RNA-seq
log2 fold change of flanking genes
TBP Bound inserts
All inserts
E
TBP
IgG
MT2_Mm
MT2_Mm (n = 2776)
-0.5 start end 0.5
distance (kb)
F
TBP
IgG
MT2_Mm down
MT2_Mm n.s.
MT2_Mm up
Down (n = 58)
Non-significant (n = 1707)
Up (n = 76)
-0.5 start end 0.5
distance (kb)

 © The Author(s)

Figure EV5. TBP binds to and regulate MaLR during preimplantation development.

(**A**, **B**) Signal aggregate plots and heatmaps of TBP enrichment (left) or IgG control (right) from pooled CUT&Tag replicates (at the late 2-cell stage) over all ORR1A0 (**A**), ORR1A1 (**B**) insertions. Start and end refer to the position of the LTR (ORR1A0, ORR1A1). *n* is the number of insertions per TE family indicated. (**C**, **D**) Boxplots showing the median and interquartile ranges of the log2 fold change (LFC) of genes with their TSS positioned within 10 kb of bound inserts of ORR1A1 (**C**) and ORR1A0 (**D**). Inserts are qualified as "bound" inserts when they belong to the top 25% inserts based on CUT&Tag heatmap signal shown in (**A**) and (**B**). Whiskers are the highest and lowest value within 1.5 times the IQR. The numbers in red correspond to the number of genes per category as indicated. Statistical significance was computed using t-test. NS, non-significant. (**E**) Signal aggregate plots and heatmap of TBP enrichment (left) or IgG control (right) from pooled CUT&Tag replicates (at the late 2-cell stage) over all MT2_Mm insertions. Start and end refer to the position of the LTR. *n* is the number of insertions per TE family indicated. (**F**) Signal aggregate plot and heatmap of TBP enrichment (left) or IgG control (right) from pooled CUT&Tag replicates (at the late 2-cell stage) over the downregulated (padj < 0.05), non-significant and upregulated (padj < 0.05) MT2_Mm LTRs upon TBP LOF relative to CONTROL embryos. Start and end refer to the position of the LTR. *n* is the number of insertions per category indicated.
