# Supplementary material for: TBP regulates transposable element expression in early mouse embryos
Source: EMBO J. 2026 Mar 20;45(9):3073–101. doi: 10.1038/s44318-026-00736-w (PMC13144441; doi:10.1038/s44318-026-00736-w)
Supplement: Supplementary file 4 — Peer Review File [file 44318_2026_736_MOESM4_ESM.pdf]

TBP regulates transposable element expression in early mouse embryos

Clara Hermant, Carlos Michel Mourra-Diaz, Marlies Oomen, Natasha Jansz, Camille Noll, Antoine Canat, Mrinmoy Pal, Tsunetoshi Nakatani, Tamas Schauer, and Maria Elena Torres-Padilla

Corresponding author: Maria Elena Torres-Padilla (torres-padilla@helmholtz-muenchen.de)

Review Timeline:

Submission Date:	26th Feb 25
Editorial Decision:	13th May 25
Revision Received:	7th Sep 25
Editorial Decision:	25th Nov 25
Revision Received:	17th Dec 25
Accepted:	23rd Jan 26

Editor: Cornelius Schneider

Transaction Report:

(Note: No Peer Review Process File is available with this article, as the authors have chosen not to make the review process public.)
